# Neural Variability and Sampling-Based Probabilistic Representations in the Visual Cortex

**DOI:** 10.1016/j.neuron.2016.09.038

**Published:** 2016-10-19

**Authors:** Gergő Orbán, Pietro Berkes, József Fiser, Máté Lengyel

**Affiliations:** 1 Computational and Biological Learning Lab, Department of Engineering, University of Cambridge, Cambridge CB2 1PZ, UK; 2 MTA Wigner Research Center for Physics, Budapest 1121, Hungary; 3 Volen National Center for Complex Systems, Brandeis University, Waltham, MA 02454, USA; 4 Department of Cognitive Science, Central European University, Budapest 1051, Hungary; 5 Brain & Cognitive Sciences, University of Rochester, Rochester, NY 14627, USA

**Keywords:** vision, V1, variability, noise correlations, natural images, spontaneous activity, normative model, theory, stochastic sampling, Bayesian computations

## Abstract

Neural responses in the visual cortex are variable, and there is now an abundance of data characterizing how the magnitude and structure of this variability depends on the stimulus. Current theories of cortical computation fail to account for these data; they either ignore variability altogether or only model its unstructured Poisson-like aspects. We develop a theory in which the cortex performs probabilistic inference such that population activity patterns represent statistical samples from the inferred probability distribution. Our main prediction is that perceptual uncertainty is directly encoded by the variability, rather than the average, of cortical responses. Through direct comparisons to previously published data as well as original data analyses, we show that a sampling-based probabilistic representation accounts for the structure of noise, signal, and spontaneous response variability and correlations in the primary visual cortex. These results suggest a novel role for neural variability in cortical dynamics and computations.

## Highlights

• Stochastic sampling links perceptual uncertainty to neural response variability
• Model accounts for independent changes in strength and variability of responses
• Model predicts relationship between noise, signal, and spontaneous correlations
• Stimulus statistics dependence of response statistics is explained

## Teaser

Orbán et al. show that linking perceptual uncertainty to neuronal variability accounts for systematic changes in variability and covariability in simple cells of the primary visual cortex. The theory also establishes a formal relationship between signal, noise, and spontaneous correlations.

## Introduction

Neural responses in sensory cortices are notoriously variable: the same stimulus can evoke a different response on each presentation (Henry et al., 1973, Tomko and Crapper, 1974). While there have been great advances in characterizing the detailed patterns and statistical structure of cortical variability (Ecker et al., 2014, Goris et al., 2014, Kohn and Smith, 2005, Lin et al., 2015), its computational relevance has received far less attention. Indeed, the consequences of cortical variability have almost exclusively been studied from the perspective of neural coding, where variability is considered as pure noise or nuisance (Carandini, 2004, Moreno-Bote et al., 2014, Shadlen and Newsome, 1998, Tolhurst et al., 1983). Conversely, computational theories of cortical representations (Adelson and Bergen, 1985, Karklin and Lewicki, 2009, Olshausen and Field, 1996, Schwartz and Simoncelli, 2001) and dynamics (Churchland et al., 2012, Hennequin et al., 2014b, Mante et al., 2013, Rigotti et al., 2013, Rubin et al., 2015) focused only on trial-average responses, either ignoring variability altogether or considering only a simple scaling of variability with average responses (Ma et al., 2006).

Here, we argue that the rich structure of neural variability in sensory cortices reveals a key aspect of cortical computations: the representation of perceptual uncertainty. The need to represent uncertainty is the logical consequence of formalizing perception as unconscious inference (Helmholtz, 1962). For example, our retinal activations can have several different interpretations in terms of the composition and arrangement of objects in the environment, each being valid with a different probability. Thus, the uncertainty inherent in perceptual inference can be formalized as a probability distribution over possible perceptual interpretations of our input (Knill and Richards, 1996). The question is, then, how do neural activities represent probability distributions (Fiser et al., 2010)? We propose that probability distributions are directly represented by the variability of cortical responses.

To study the implications of representing uncertainty through neural variability, we developed a model of population responses in the primary visual cortex (V1) with three main assumptions. First, we posit that neural activity patterns represent statistical samples from a probability distribution over visual features of a scene (Fiser et al., 2010, Hoyer and Hyvarinen, 2003, Lee and Mumford, 2003). Second, we specifically propose that individual samples in the model are represented by the membrane potentials (or, equivalently, the instantaneous firing rates) of neurons. Third, as the autocorrelations of membrane potentials for any static stimulus typically decay on a relatively short (∼20 ms) timescale (Azouz and Gray, 1999), membrane-potential values (and consequently firing rates) separated on this timescale are considered statistically independent and therefore are modeled as independent stochastic samples from the underlying probability distribution. This naturally gives rise to within- as well as across-trial variability in the model.

This proposed representational scheme has two main implications. First, the set of responses (i.e., membrane-potential values) at any time in a population of neurons in V1 represents a combination of visual features as a possible interpretation of the input. Second, the within-trial variability of responses is such that the relative frequency with which any population pattern is visited is equal to the probability that the corresponding combination of features is a valid interpretation of the visual scene. Thus, neural response variability is directly linked to uncertainty about the stimulus: the wider the inferred range of possible feature combinations is, the wider the distribution of responses will become. In contrast to earlier proposals for how uncertainty may be represented in cortical activities (Deneve, 2008, Ma et al., 2006, Rao, 2004, Zemel et al., 1998), this establishes the mean and variability of responses as independent information channels, respectively encoding the mean and the associated uncertainty of the probability distribution over visual features. Importantly, these predictions about within-trial variability can also be tested in variability measured across trials that use the same stimulus and thus elicit the same probability distribution from which responses are sampled.

To test our model, we systematically compared the neural variability that our model predicted in response to various visual stimuli with the across-trial variability recorded in V1 in response to the same set of stimuli. As the parameters of our model were fundamentally determined by the statistical properties of visual scenes, rather than the properties of V1 circuits, this approach allowed a strong test of the model. Specifically, we show that the sampling-based representation of our model accounts for several key properties of response variability in V1. First, response variability not directly related to the stimulus can be so high that it dominates evoked responses (Arieli et al., 1996, Fiser et al., 2004, Vogels et al., 1989). Second, just as mean responses show systematic changes with particular attributes of the stimulus (as characterized by tuning curves), so does the variability of responses. In particular, experimental manipulations of image contrast or aperture (known to control perceptual uncertainty; Weiss et al., 2002) modulate the magnitude of variability largely independently from changes in mean responses (Churchland et al., 2010); conversely, changes in the orientation of the stimulus (which do not influence uncertainty) mainly affect the trial average of responses, and affect their relative variability much less. Third, response variability exhibits systematic patterns not only in its overall magnitude but also in its fine structure: signal correlations bear a specific relationship to noise (Ecker et al., 2010) and spontaneous correlations. Fourth, more generally, the structure of response variability during evoked activity closely resembles variability during spontaneous activity (Arieli et al., 1996, Berkes et al., 2011a, El Boustani et al., 2009, Fiser et al., 2004). In order to test and evaluate these implications of the model quantitatively, we compared model results directly to previously published experimental results whenever possible. To confirm the specific new predictions of the model about the structure and stimulus-dependent modulation of spike-count variability, we further performed novel analyses of a published dataset of V1 recordings from awake macaques (Ecker et al., 2010). These results suggest a new perspective on the functional role of variability in cortical dynamics and distinguish between previous conflicting proposals about how uncertainty is represented in the cortex.

## Results

### From Natural Image Statistics to Neural Representations

**Figure 1:**
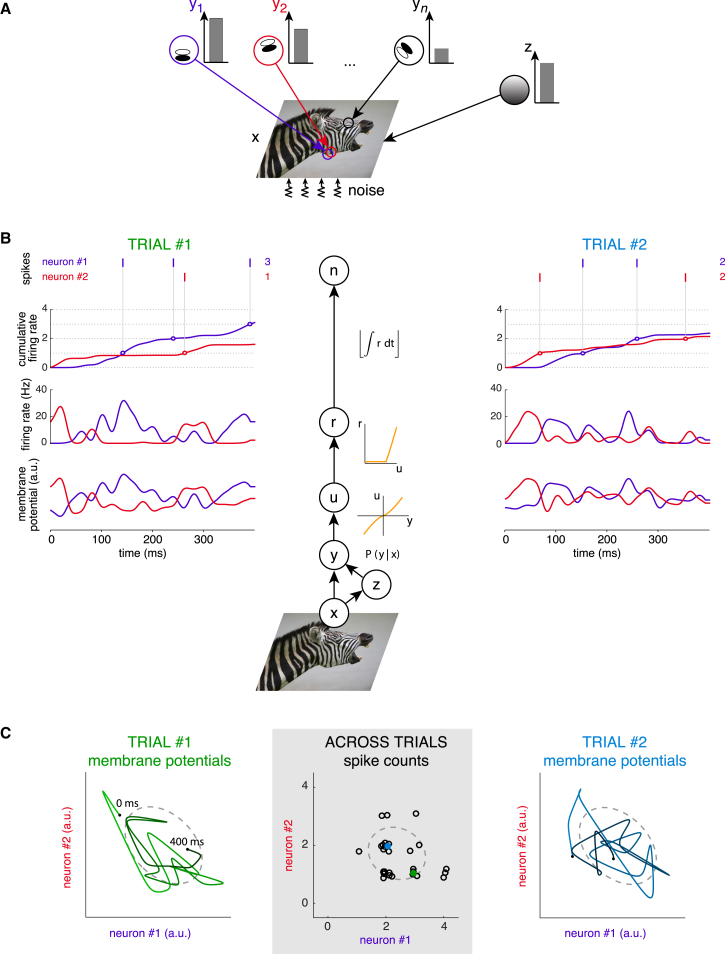
Schematic of the Model (A) The generative model describing the statistical structure of image patches (**x**). Images arise as a linear combination of Gabor-filter basis functions with intensities **y**$=\left\{y_{1},\ldots ,y_{n}\right\},$ whose contribution to the image is jointly scaled by a “contrast” variable, z, plus Gaussian white noise (see Experimental Procedures for details). (B) Probabilistic inference and the generation of membrane potentials and spike counts. The progression of four steps in the model is shown in the middle of the panel, advancing from the bottom toward the top. The activations of two example cells in red and purple (see the corresponding basis functions in A) are illustrated in two different trials using the same stimulus, x (left and right sides in B). Basis function activations, y, are inferred by inverting the generative process shown in (A). Due to noise and ambiguity in the model, y cannot be inferred from the image with certainty; hence, the result of Bayesian inference is a posterior probability distribution, P(y|x). Membrane-potential values, u, represent stochastic samples from P(y|x) through a weak non-linear transformation (inset), with independent samples drawn every ∼20 ms, corresponding to typical autocorrelation timescales of V1 neurons (Azouz and Gray, 1999) (For illustration, membrane potentials are plotted after smoothing with a 7-ms Gaussian kernel here. See also Experimental Procedures). Instantaneous firing rates, r, are obtained from membrane potentials by a rectifying non-linearity (Carandini, 2004; inset). Spike counts are obtained by deterministically integrating firing rates across time over the duration of a trial: a spike is fired whenever the cumulative firing rate reaches an integer value (open circles on cumulative firing-rate traces and ticks in spike rasters, with the final spike counts shown at the right end of each raster). Note that while the distribution of neural responses (mean, variance, and covariance) remains unchanged across trials using the same stimulus, the actual time course of membrane potentials and the spike counts can vary substantially across trials due to stochastic sampling from the same underlying distribution. (C) Statistics of the joint activity of a pair of neurons. The two sides show the membrane-potential trajectories of the pair of neurons in the two trials presented in (B) plotted against each other, revealing the higher-order statistics of the joint distribution (e.g., non-zero correlations). Colored lines correspond to the membrane-potential trajectories shown in (B) (color shade indicates elapsed time), and dashed gray ellipses show the covariance underlying the stochastic trajectories (identical for the two trials). The center shows joint spike-count distribution of the same two cells across a large set of trials (circles) for the same stimulus. The two colored circles correspond to the spike counts obtained from the two trials shown at the two sides and presented in (B). Small jitter was added to integer spike counts for illustration purposes. Photo is from Istock.com/CliffParnell.

We extended a well-known family of representational models of V1, in which the visual cortex maintains an internal model of how images are generated by underlying visual features (Figure 1A; see also Figure S1, Experimental Procedures, and Supplemental Experimental Procedures). According to this internal model, an image patch is generated by a multiplicative interaction between two terms (plus noise):

(Equation 1) $$\text{image}=z\times \left(\sum_{i}{\text{activation}}_{i}\times {\text{basis}}_{i}\right)+\text{noise}\text{.}$$

The first term, z, which we assumed for simplicity to be a single scalar, determines the global contrast level of the image patch. The second term is a linear combination of basis functions, and simple cell activations represent the coefficients with which each of these basis functions contribute to the image (Olshausen and Field, 1996, Schwartz and Simoncelli, 2001). In addition, the internal model also defines the prior probability distribution of basis function activations, P(activations), which expresses the frequency with which any combination of activations is expected to occur across different images. The role of V1 is then to invert this generative process and infer the level of activation for each feature in an image (Karklin and Lewicki, 2009, Olshausen and Field, 1996, Rao and Ballard, 1999, Schwartz and Simoncelli, 2001; Figure 1B, bottom; Experimental Procedures). The result of inference is a posterior distribution, P(activations|image), expressing the probability that any particular combination of features may underlie the current input.

Despite behavioral evidence for the representation of uncertainty (Ernst and Banks, 2002, Weiss et al., 2002), most previous representational models assumed that neural activities represent a single combination of features for each input (Karklin and Lewicki, 2009, Olshausen and Field, 1996, Rao and Ballard, 1999, Schwartz and Simoncelli, 2001), such as the one with the maximum posterior probability. These models were thus unable to capture the uncertainty expressed by the extent of the posterior. In contrast, our model maintained a representation of uncertainty by neural activities encoding randomly sampled feature combinations under the posterior. That is, the relative occurrence frequency of any neural activity pattern was equal to the inferred probability that the feature combination represented by it may have generated the input image. More specifically, we assumed that samples from the posterior were represented by the fluctuating membrane potentials of V1 cells through a weak compressing non-linearity, and we derived the instantaneous firing rate of a cell as a rectified-nonlinear function of its membrane potential (Carandini, 2004; Figure 1B, top; Supplemental Experimental Procedures). Thus, we took the membrane-potential values in a population of cells at any moment in time to represent a single sample from the multidimensional posterior, so that subsequent membrane potential values represented a sequence of samples (Figure 1C). This allowed us to make predictions about the form of the resulting distribution of neural activities in V1 without assuming a specific form for the underlying neural circuit dynamics.

### Key Features of Neural Response Variability in the Model

**Figure 2:**
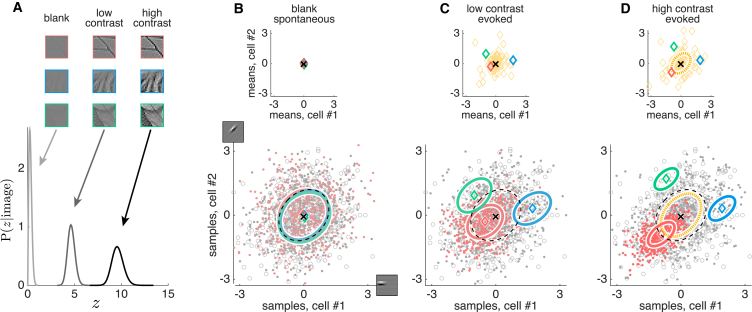
Key Features of Response Variability in Model Membrane Potentials (A) Three example images (identified by the color of their frame) are shown at increasing contrast levels from left to right. Increasing the contrast shifts the posterior over the inferred contrast level, z, away from zero (gray distribution curves, from light to dark). (B) Joint membrane-potential distributions of two example neurons (images at the end of axes show corresponding basis functions) for the three sample images in (A) at low contrast (colored diamonds, means; colored ellipses, covariances for the three images). Colors correspond to image frames in (A) compared to the prior distribution (black cross, mean; dashed black ellipse, covariance). The prior and the three posteriors strongly overlap; therefore, samples drawn from these distributions (gray circles, prior; red dots, posterior for image with red frame; gray dots, average posterior across 100 different images) and their means (crosses and diamonds) are indistinguishable. Inset on top shows the prior mean (black cross) and posterior means for the three natural image patches presented in (A) (colored diamonds). (C and D) Shown as in (B), but for two higher contrast levels. The posteriors for the three images increasingly deviate from the prior and each other: their mean moves further away from zero while their covariances (noise covariances) shrink and remain similar. Signal covariance (yellow dotted ellipse in D) is aligned with the covariance of the prior (black dashed ellipse). Individual posteriors tile the subspace covered by the spontaneous covariance, such that samples drawn from the average posterior (gray dots), but not those drawn from any individual posterior (red dots), still overlap with those from the prior (gray circles). Insets on top show prior mean (black cross) and posterior means for the three images in (A) (red, green, and blue diamonds) as well as for 100 other natural image patches (yellow diamonds). In contrast to the decrease in noise covariances, signal covariances (covariances of posterior means across stimuli) increase with increasing contrast levels.

Interpreting neural population activity patterns as samples from the posterior distribution of the internal model determined by Equation 1 establishes a direct link between the parameters of the posterior and the statistics of population responses. For example, the mean and the covariance of the posterior given a particular input image respectively correspond to the average and covariance of the neural responses evoked by that image. Thus, understanding the basic properties of the posterior distribution, and their dependence on the stimulus, provides key insights about the stimulus-dependent changes of cortical variability predicted by our model, which can be most directly demonstrated in the membrane-potential responses of a pair of model neurons (Figure 2).

The variability of the average response of each cell across different stimuli is predicted by the dependence of the posterior mean on the image. As the basis functions in our model are oriented Gabor filters that are assumed to combine linearly in the image (Equation 1), the posterior mean of the activation of each basis function is largely determined by its linear overlap with the stimulus (Experimental Procedures; Equation 5). Thus, as in earlier models (Olshausen and Field, 1996), the trial-average response for simple oriented stimuli (such as commonly used full-field gratings) depends monotonically on the similarity of the “preferred orientation” of a cell (the orientation of its basis function) and the orientation of the stimulus, resulting in orientation-dependent tuning curves (Figure S2).

Changes in image contrast lead to corresponding changes in the inferred level of contrast, z (Figure 2A). A low-contrast image provides less evidence about the exact content of the image, so inferences rely predominantly on prior expectations, P(activations). In the extreme case of a blank stimulus, z approaches zero (Figure 2A, light gray), so inferences about the basis function activations that neurons represent are unconstrained by the image (Equation 1 is constant with respect to the activations), and thus the posterior becomes entirely determined by the prior (Berkes et al., 2011a, Fiser et al., 2010). In other words, spontaneous activity, as a special case of evoked activity recorded in response to a blank stimulus, represents samples from the prior (Figure 2B).

**Figure 3:**
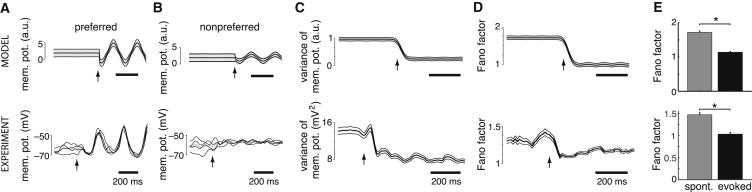
Stimulus Onset Quenches Neural Variability (A and B) Periodic membrane-potential oscillations induced in an example neuron by a drifting sinusoid grating stimulus with preferred (A) and non-preferred (orthogonal to preferred) orientation (B) appearing after a blank image. Variability of responses is shown by their standard deviation (flanking gray area) for the model (top), and by individual trajectories in example trials (thin black lines) for the experimental data (bottom). Thick black (top) and gray (bottom) lines show across-trial average. Arrows mark stimulus onset. (C and D) Population analysis of the effect of stimulus onset on the variance of membrane potentials (C) and the Fano factor of spike counts (D). Arrows mark stimulus onset; thick black lines and flanking thin lines show population average and SE. (E) Direct comparison of spike-count Fano factors during spontaneous activity in response to a blank stimulus and evoked activity in response to high-contrast stimuli. Bars show population average, error bars indicate 95% bootstrap confidence intervals, ^∗^p < 0.05. In each panel, the top plot shows the model results, and the bottom plot presents experimental data. Experimental data in (A)–(D) were reprinted by permission from Macmillan Publishers Ltd: Nature Neuroscience (Churchland et al., 2010, intracellular recordings in anesthetized cat). (E) presents an analysis of data from Ecker et al. (2010) (extracellular unit recordings in awake macaque). Fano factors in (D) and (E) were computed after mean matching (see Supplemental Experimental Procedures).

**Figure 4:**
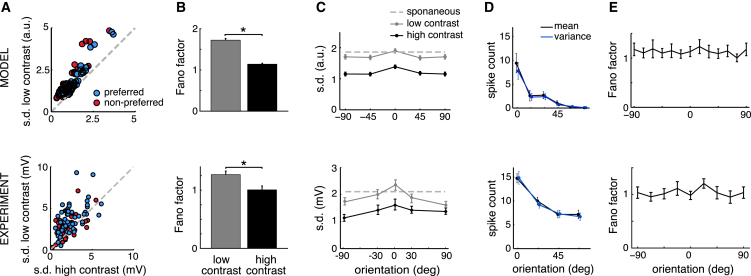
Stimulus Dependence of Neural Response Variability (A) Across-trial SD of peak response amplitudes of a population of cells (circles) for low-contrast gratings plotted against the SD for high-contrast gratings at the preferred (blue) and non-preferred (red) stimulus orientation. (B) Spike-count Fano factors (mean matched) for low- and high-contrast stimuli. (C) Dependence of membrane potential SD on grating orientation at high (solid black line) and low (solid gray line) contrast. For reference, membrane potential SD during spontaneous activity recorded in response to a blank stimulus is also shown (dashed gray line). (D and E) Mean and variance (black and blue lines in D) and Fano factor (E) of spike counts as a function of stimulus orientation relative to the preferred orientation of the cell. (B)–(E) show population averages (bars or lines), with error bars showing 95% bootstrap confidence intervals (B) and SE (C)–(E), ^∗^p < 0.05. Experimental data in (A) and (C) were reproduced from Finn et al. (2007) with permission from Cell Press (intracellular recordings in anesthetized cat), and (B), (D), and (E) present analyses of data from Ecker et al. (2010) (extracellular unit recordings in awake macaque).

**Figure 5:**
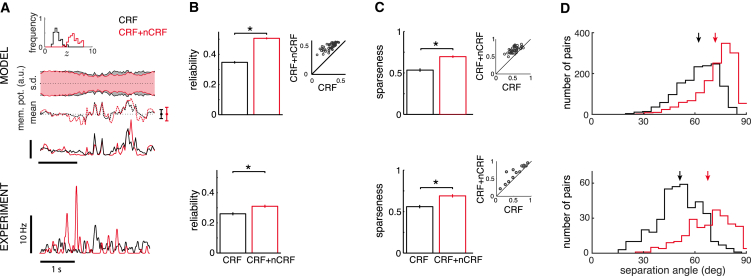
The Effect of Aperture on Response Reliability, Sparseness, and Signal Correlations (A) The response of a representative neuron to repeated presentation of an image sequence constrained to the classical receptive field (CRF, black) or combined non-classical receptive field (nCRF) and CRF stimulation (CRF + nCRF, red). Model plots from top to bottom show distribution of inferred contrast levels, z, across frames of the stimulus movie (histograms); the SD (shaded area) and mean of the membrane potential (dotted lines, error bars to the right show signal variability); and the trial-average firing rate (solid lines) of a representative neuron across time. Experimental data show trial-average firing rate. (B) Reliability of membrane-potential responses with CRF-only and combined nCRF + CRF stimulation. Inset (top) shows changes in the reliability for individual neurons. (C) Lifetime sparseness of firing rates with CRF-only and combined nCRF + CRF stimulation. Insets show changes in sparseness for individual neurons. (D) Distribution of separation angles between the mean response vectors of cell pairs with overlapping CRFs for CRF-only and combined nCRF + CRF stimulation. Arrows mark average separation angles. A higher separation angle means lower signal correlation. (B) and (C) show population averages (bars or lines) with error bars showing SE, ^∗^p < 0.05. Experimental data in (A)–(C) were reproduced from Haider et al. (2010) with permission from Cell Press (intracellular recordings in anesthetized cat), and those in (D) were reprinted from Vinje and Gallant (2000) with permission from AAAS (extracellular recording from awake macaque).

For higher contrast levels, the inferred level of z also grows (Figure 2A, dark gray and black), so that the input image increasingly constrains the posterior of basis-function activations, which thus increasingly deviates from the prior (Figures 2C and 2D). This deviation has two major aspects. First, the mean of the posterior becomes different from the prior mean, and will be specific to the particular image that gave rise to it. This implies that signal variability, the variability of the mean response across different stimuli, grows with contrast (Figures 2B–2D, insets on top). Second, the observation of a high-contrast image reduces uncertainty (on average) about basis function activations relative to the prior. Thus, the (co)variance of individual posteriors will be smaller than that of the prior, implying that noise (co)variances, the across-trial variability of neural responses to the same stimulus, must decrease with increasing contrast (e.g., red covariance ellipses across Figures 2B–2D; see also Figures 3, 4A, 4B, and 5C). As opposed to the mean of the posterior (cf. Figure S2), its covariance does not show a strong dependence on the detailed content of the stimulus beyond its overall contrast (red versus green versus blue covariance ellipses within Figures 2B–2D; see also Figures 4C–4E). This is intuitive; for example, changing the orientation of a grating, as opposed to its contrast, does not influence our uncertainty about it.

**Figure 6:**
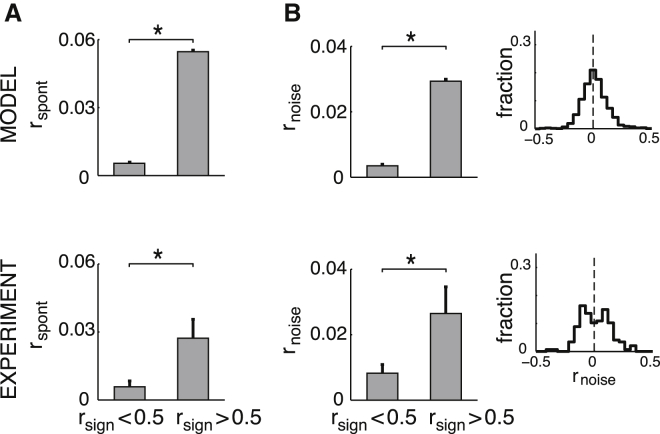
Relationship between Signal, Noise, and Spontaneous Correlations (A) Dependence of correlations during spontaneous activity, r_spont_, on spike-count signal correlations, r_sign_. (B) Dependence of noise correlations during evoked activity, r_noise_, on signal correlations. Bars show averages across cell pairs with signal correlations below or above the r_sign_ = 0.5 threshold, as shown on the x axis; error bars show SE, ^∗^p < 0.05. Insets show the distribution of noise correlations; dashed line shows the mean of the distribution. Bottom panels present analyses of data from Ecker et al. (2010) (extracellular unit recordings in awake macaque).

**Figure 7:**
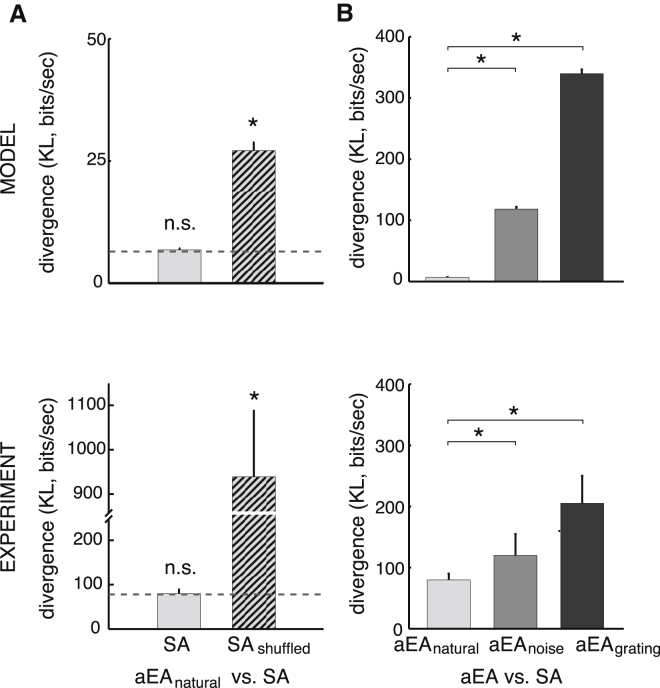
Match between Spontaneous and Average Evoked Activity Multi-Unit Distributions Depends on Correlations and the Stimulus Ensemble Used (A) Kullback-Leibler (KL) divergence between aEA for natural image patches (aEA_natural_) and SA (light gray bar), and between aEA_natural_ and a shuffled version of SA, preserving individual firing rates but destroying all correlations across electrodes (SA_shuffled,_ hatched bar). For reference, baseline KL divergence between two halves of SA data is also shown (dashed line). (B) KL divergence between aEA and SA under three different stimulus conditions: natural image patches (aEA_natural_, light gray bar, same as in (A); random block noise images (aEA_noise_, dark gray bar); and grating stimuli with various phases, orientations, and frequencies (aEA_grating_, black bar). In all panels, bars show averages across animals and error bars show SE, ^∗^p < 0.05. Bottom panels present analyses of experimental data from Berkes et al. (2011a) with permission from AAAS (extracellular multi-unit recordings in awake ferrets).

As long as the internal model is well-adapted to the statistics of stimuli, it can be shown that its prior, P(activations) (Figures 2B–2D, gray circles), must match the average posterior, ${\langle \text{P}\left(\text{activations}|\text{image}\right)\rangle }_{\text{P}\left(\text{image}\right)}$, averaged across the distribution of stimuli, P(image), to which it has been adapted (Gelman et al., 2013; Figures 2B–2D, gray dots). As for high-contrast images, noise variability in responses is low, but signal variability is high (see above; compare the size of the yellow covariance ellipse to that of the red-green-blue covariance ellipses in Figure 2D); most of the response variability is due to signal variability; and thus, spontaneous correlations (see above; reflecting the prior) are predicted to largely follow signal correlations (compare black dashed and yellow dotted covariance ellipses in Figure 2D; see also Figure 6A). As a consequence, we were also able to show in our model (Supplemental Experimental Procedures) that noise correlations will also be similar to signal correlations (compare the shape of the yellow covariance ellipse to red-green-blue covariance ellipses in Figure 2D; see also Figure 6B). More generally, the matching of the average posterior to the prior predicts a match between the distribution of spontaneous activities and the average distribution of evoked activities (compare the scatter of empty and filled circles in Figures 2C and 2D; see also Figure 7) (Berkes et al., 2011a).

In the following, we test each of these key features of our model in neural data. For this, most parameters of the model were set according to the statistics of natural image patches, without regard to neural data, leaving only four free parameters to determine how sampled feature values under the posterior were mapped to membrane potentials and firing rates in V1 neurons (Experimental Procedures). Out of these four parameters, we determined one based on previous literature and tuned only three to fit specific experimental data recorded in V1. The experimental data to be reproduced were selected by a set of predetermined criteria regarding both the type of neural data recorded and the stimulus manipulations used in the experiments (Supplemental Experimental Procedures). Importantly, although these data included multiple species and conditions, we took a conservative approach and used a single setting of parameters across all our simulations (Table S1). For a fair comparison, in each case model responses were analyzed using the same statistical methods as those used for the analysis of the corresponding experimental dataset (Supplemental Experimental Procedures).

### Mean Responses, Tuning Curves, and Contrast Invariance

In order to establish the validity of our model at a basic level, we first validated the model by reproducing some fundamental aspects of the mean responses of V1 simple cells. For this, we followed the method by which tuning curves are measured experimentally and computed average responses in the model for full-field grating stimuli with different orientations. As expected, our model neurons possessed clear orientation tuning for both membrane potentials and firing rates as found experimentally (Figures S2A and S2B). Importantly, despite the failure of previous attempts to reconcile sampling-based probabilistic representations with contrast invariant tuning curves (Pouget et al., 2013), firing-rate tuning curves in the model also showed contrast invariance (Skottun et al., 1987); i.e., only their amplitude scaled with contrast, but their width remained roughly constant (Supplemental Experimental Procedures; Figures S2B–S2E). This meant that, unlike models in which neuronal activity is proportional to probabilities (Pouget et al., 2013), our model did not suffer from the unrealistic property of tuning curves becoming exceedingly narrow at high-contrast levels, as high certainty was encoded by small noise variability instead (Figure 2). Moreover, our model also reproduced various characteristic non-classical receptive field (nCRF) effects, such as cross-orientation suppression and surround suppression (Bonds, 1989, Cavanaugh, 2001, Schwartz and Simoncelli, 2001; Supplemental Experimental Procedures; Figures S2F–S2H).

### Response Variability and Stimulus Onset

The decrease in noise variability with contrast (Figure 2) in our model predicts that a high-contrast image following a blank period should lead to decreasing variability in V1 membrane-potential responses, and that this effect should hold regardless of whether or not the stimulus is aligned with the preferred orientation of a cell (Figures 3A–3C, top). Moreover, these changes in membrane-potential variability should carry over to changes in spike-count Fano factors even with the effects of changes in mean firing rates being factored out (Churchland et al., 2010; Figures 3D and 3E, top, two-sample t test, n = 90, p < 10^−4^, t[178] = −5.4; Figure S3D; see also Supplemental Experimental Procedures). Such quenching of variability at stimulus onset is a general feature of cortical responses reported under a wide variety of experimental conditions (Churchland et al., 2010); in particular, it has been observed in recordings from V1 simple cells of anesthetized cats (Figures 3A–3C, bottom) and monkeys (Figure 3D, bottom). Furthermore, our analysis of recordings from awake macaques (Ecker et al., 2010) shows that this effect is also present in the awake V1 (Figure 3E, bottom, two-sample t test, n = 800, p < 10^−4^, t[1,598] = 37.3).

### Contrast and Orientation Dependence of Noise Variability

Behavioral studies indicate that stimulus contrast directly affects subjective uncertainty (Weiss et al., 2002). This is consistent with the inverse scaling of posterior (co)variances with contrast in the model, which in turn predicts a similar scaling of noise (co)variances in V1 responses (Figures 2B–2D). Indeed, our model generated systematically higher membrane-potential variances for low- versus high-contrast stimuli (Figure 4A, top; paired t test, n = 61, t[60] = −6.02, p < 10^−4^, and t[60] = −6.28, p < 10^−4^ for stimuli with preferred and non-preferred orientations, respectively). Once again, this difference between the variances at high and low contrast was present for preferred as well as non-preferred stimuli (Figure 4A, top). The same pattern of results had been obtained experimentally from V1-simple cells of anesthetized cats (Finn et al., 2007; Figure 4A, bottom). The decrease in model membrane-potential variability was also reflected in a decrease in spike-count Fano factors (mean matched, see Supplemental Experimental Procedures; Figure 4B, top; two-sample t test, n = 102, t[200] = −4.32, p < 10^−4^). Our analysis of data recorded in awake-monkey V1 also showed a similar decrease in (mean matched) Fano factors with increasing contrast (Figure 4B; bottom; two-sample t test, n = 800, t[1,598] = 37.3, p < 10^−4^), confirming that it could not be attributed to the confounding effects of anesthesia, in which slow, synchronized activity fluctuation can have a major impact on measures of variability (Ecker et al., 2014, Goris et al., 2014; see also Supplemental Experimental Procedures and Figure S4A). Moreover, at the same time that noise variability decreased with contrast in the model, signal variability increased (Figures 2 and S2)—in agreement with experimental data showing a general scaling of average membrane-potential and firing-rate responses with contrast (Finn et al., 2007, Skottun et al., 1987), and in disagreement with a potentially simpler linear mechanism according to which both signal and noise variability would originate from the same form of contrast-dependent variability in the input (Moreno-Bote et al., 2014).

As opposed to contrast, the orientation of a stimulus primarily affects the mean estimate of how much the feature represented by a neuron contributes to the stimulus (reflected in the tuning curves of mean responses, Figure S2), and only much more moderately affects the uncertainty associated with this estimate (Figure 2, see also Supplemental Experimental Procedures). Confirming this observation, the membrane-potential variances in our model showed only mild modulation by stimulus orientation (Figure 4C, top). These results agreed with intracellular measurements showing a similar pattern of change in V1 simple cells of cats, with a small peak in the membrane-potential variance at the preferred stimulus orientations of neurons (Finn et al., 2007; Figure 4C, bottom).

The rectifying non-linearity that maps membrane potentials to firing rates in our model converted orientation-dependent changes in the mean membrane potential to changes in both the mean and the variance of spike counts (Figure 4D). However, as sampling resulted in the variance of membrane potentials remaining constant this time (as opposed to when contrast was changed, Figure 3), changes in spike-count variance were only as large as those in mean spike counts, such that the Fano factor of the spike-count distribution remained constant over the whole range of orientations (Figure 4E, top, one-way ANOVA p = 0.98, F[11,108] = 0.30). These predictions of the model have been confirmed by our analysis of awake-monkey recordings in V1 (Figures 4D and 4E, bottom, one-way ANOVA p = 0.47, F[71,012] = 0.55).

### The Effect of Aperture on Response Variability, Sparseness, and Correlations

Although the generative process underlying our model specifies a relatively simple, largely linear mechanism for how natural image patches are generated as a combination of basic visual features (Equation 1; Figure 1A), inverting this process to infer the features from an image typically results in a complex posterior distribution that depends non-linearly on the image pixels. This complexity arises due to the so-called “explaining away” effect (Pearl, 1988), a common consequence of probabilistic inference, by which even distant pixel values that are unaffected by a visual feature under the generative process can indirectly influence the inferred value of that feature under the posterior. For example, in our model, all pixels in the image contribute to the inferred value of global contrast, z, which in turn influences the activity of all neurons (Figures 1 and 2), so even those portions of the image which are not part of the visual feature (basis function) represented by a neuron can change its activity.

As a result of explaining away, just as trial-average responses (tuning curves) were modified by suitable extra-classical receptive field (eCRF) stimuli (see above and Figure S2), so too were the higher-order statistical moments of responses subject to such eCRF effects in our model. In particular, presenting the same natural movie sequence stimulus under a larger aperture that included both the classical receptive field (CRF) and the surround nCRF of a cell increased the effective contrast content of the input image (total variation in pixel values over the image), and thus led to a higher inferred value of z (Figure 5A, histograms). In other words, changes in aperture had effects on model inferences which were fundamentally analogous to changes in contrast (cf. Figure 2). In particular, just as when increasing contrast, an increase in inferred z resulted in higher signal variance and lower noise variance in membrane potentials (Figure 5A, dotted lines and shaded areas; cf. Figure 2) and thus more reliable membrane-potential responses (Figure 5B, top, one-sample t test, n = 54, t[53], 9.18, p < 10^−4^). In turn, these opposite changes in signal and noise variability of membrane potentials meant that a larger fraction of the membrane-potential distribution of a cell lay respectively above or below the threshold for its preferred and non-preferred stimuli (frames of the movie). This increased the number of stimuli that evoked no firing in a cell while also increasing the firing rate for those stimuli that did evoke firing in it, and hence led to sparser spiking responses (Figures 5A, top solid line, and 5C, top, one-sample t test, n = 54, t[53] = −20.1, p < 10^−4^). As the response of each neuron became sparser, these responses also became more de-correlated from each other, as reflected by the higher separation angles between the response vectors of neuron pairs with overlapping CRFs (Figure 5D, top, one-sample t test, n = 1,431, t[1,430] = −43.4, p < 10^−4^). These results reproduced experimental data recorded in the anesthetized cat (Figures 5A–5C, bottom; Haider et al., 2010) and the awake monkey (Figure 5D, bottom; Vinje and Gallant, 2000) under similar stimulus manipulations. We found that the same mechanism also accounted for why phase scrambling of natural images, which decreased the overall local-contrast content of an image, led to less sparse responses in V1 in other experiments (Froudarakis et al., 2014; data not shown).

Next, we wanted to test whether the stimulus dependence (i.e., contrast and aperture dependence) of the variability of neural responses reproduced by our model (Figure 3, Figure 4, Figure 5) conveyed significant information about the stimulus beyond that information conveyed by mean responses. For this, we measured how well the stimulus could be decoded by taking into account or ignoring these stimulus-dependent variability modulations. We found that the decoding performance of an optimal decoder (which took all aspects of response distributions into account) was often substantially higher than that of a linear decoder (which assumed no changes in spike-count Fano factors; Figure S5; Supplemental Experimental Procedures). Thus, in contrast to other proposed population coding schemes for uncertainty (Ma et al., 2006), the sampling-based population code of our model was not linearly decodable in general.

### Relationship between Signal, Spontaneous, and Noise Correlations

In the foregoing sections, we have demonstrated that the characteristics of the mean and the variance of individual model neuron responses in a sampling-based representation closely matched those found experimentally. In order to characterize the joint variability in the response distribution more completely, we also investigated the fine structure of correlations.

Our theory provided a principled link between various forms of response covariances and correlations during stimulus-evoked and spontaneous activity. In particular, it predicted a match between signal and spontaneous correlations as well as between signal and noise correlations (Figures 2 and S4C; see also Supplemental Experimental Procedures). Although these similarities were most cleanly predicted for membrane potentials, directly representing samples from the posterior distribution, they also carried over to firing rates and consequently to spike counts. In particular, we found a positive relationship between signal and spontaneous correlations of spike counts in the model (Figure 6A, top, two-sample t test, n = [27,232; 1,209], t[28,439] = −19.5, p < 10^−4^), which was confirmed by our analysis of awake-monkey V1 recordings (Ecker et al., 2010; Figure 6A, bottom, two-sample t test, n = [1,474; 189], t[1,661] = −2.73, p = 0.0063). A similar relationship between spontaneous and signal correlations has also been noted in the anesthetized-cat V1, but it could not be captured by previous models (Lin et al., 2015). Spike-count noise correlations also had a positive relationship with signal correlations in the model (Figure 6B, top, two-sample t test n = [27,457; 1,223], t[28,678] = −12.0, p < 10^−4^), in line with the general finding that noise and signal correlations tend to be positively related in a variety of cortical areas (Cohen and Maunsell, 2009, Gu et al., 2011) including the awake-macaque V1 (Ecker et al., 2010; Figure 6B, bottom, two-sample t test, n = [1,486; 172], t[1,656] = −2.20, p = 0.028). As our model neurons had a diverse set of receptive fields without a strong overrepresentation of any particular feature, the distribution of signal correlations was centered very close to zero (mean 0.015). As a corollary of the similarity of signal and noise correlations, the distribution of noise correlations also had a mean close to zero (Figure 6B, top inset mean 0.0074), in line with experimental findings in awake animals (Ecker et al., 2010; Figure 6B, bottom inset, mean 0.011).

### Spontaneous and Evoked Response Distributions

In the previous sections, we have shown how a sampling-based representation accounted for differences in both neural variability and correlations between spontaneous and stimulus-evoked activities as responses recorded at zero and full contrast. However, sampling also implied specific similarities between spontaneous and stimulus-evoked activities (Figure 2D, bottom). In particular, it implied that the distribution of spontaneous activity (SA) must match the average distribution of evoked activities (aEAs). Importantly, this match was only expected to hold for stimuli to which the model has been adapted, i.e., for natural images but not for artificial images. Indeed, computing the dissimilarity between SA and the respective aEAs for natural images (aEA_natural_), block noise patterns (aEA_noise_), and drifting gratings (aEA_grating_) confirmed these relationships in our model (Figures 7 and S6). More specifically, the divergence between aEA_natural_ and SA was not different from a baseline divergence computed between the two halves of SA representing the minimal divergence one could expect to see in the data (which was greater than zero due to finite sample size effects) (Figure 7A, top).

To test for the role of correlations for this match between aEA_natural_ and SA, we independently shuffled the spike trains recorded on each electrode during spontaneous activity, thus preserving individual firing rates but destroying all correlations across electrodes (Berkes et al., 2011a, Fiser et al., 2013; SA_shuffled_). This resulted in a divergence between aEA_natural_ and SA_shuffled_ that was significantly greater than baseline (Figure 7A, top, m-test, see also Berkes et al., 2011a, n = 20, m = 1.95e21, p < 10^−4^) suggesting that the correlational structure of these activities, which we analyzed in the previous section, was crucial for the match between them. Extracellular recordings of multi-unit firing patterns in the V1 of awake adult ferrets (Berkes et al., 2011a) showed the same effect but with a greater magnitude (Figure 7A, bottom), possibly due to coordinated fluctuations in overall population activity during both SA and aEA (Fiser et al., 2013) that our model did not capture. Furthermore, the divergence between aEA_natural_ and SA in the model was significantly smaller than the divergence between aEA_noise_ or aEA_grating_ and SA (Figure 7B, top, m-test, see also Berkes et al., 2011a, n = 20, m = 9.15e42, p < 10^−4^, and m = 2.97e55, p < 10^−4^, respectively). This pattern of results was also observed in our ferret dataset: responses evoked by a natural movie ensemble showed less dissimilarity in distribution from spontaneous firing patterns than those evoked by grating stimuli or block noise (Berkes et al., 2011a; Figure 7B, bottom).

## Discussion

We presented a theory of the neural representation of uncertainty in the visual cortex that provides an account of a broad range of findings regarding neural variability in V1 which had previously lacked a unifying interpretation. Importantly, the model presented here is normative—it not only aims to capture the phenomenology of V1 activity but also proposes a rational, computational principle to explain why V1 should behave the way it does. In particular, the key principle of our model is that membrane-potential values (and hence firing rates) across a population of V1 neurons at subsequent moments in time are interpreted as samples drawn from a posterior distribution over visual features. This means that the variability of neural responses directly represents uncertainty about the visual image, such that higher uncertainty is reflected in increased noise variability of neural activity. This theory provided an intuitive explanation for why increasing contrast or aperture quenches variability in V1, and why stimulus orientation has little effect on it. The model also predicted the similarity of spontaneous and evoked activities and thus accounted for the finding that spontaneous, signal, and noise correlations tend to be correlated across cell pairs. To support these predictions, we presented analytical derivations and numerical simulations of the model as well as evidence from experimental recordings, including novel data analyses.

### Distinguishing Different Probabilistic Representations

Our results provide a way to distinguish between previous conflicting proposals about the neural underpinning of probabilistic representations in the cortex (Fiser et al., 2010). These proposals broadly fall into two classes. In one class, both the mean of a probability distribution and the associated uncertainty are represented by time-average neural responses. In this class of models, changes in response variability are directly linked to changes in average responses and thus do not serve as an independent information channel (Deneve, 2008, Ma et al., 2006, Rao, 2004, Zemel et al., 1998). In the second class, which is based on sampling, the average and variability of responses encode different and complementary aspects of a probability distribution: average responses encode the mean, while variability and co-variability encode higher-order moments, such as variances and covariances, of the distribution (Fiser et al., 2010, Hoyer and Hyvarinen, 2003, Lee and Mumford, 2003). Therefore, the main empirically testable difference between sampling-based and most other types of probabilistic representations, such as probabilistic population codes (Ma et al., 2006), is that variability is controlled independently of mean responses in the former, while in the latter the mean and variance are coupled by a fixed constant of proportionality. Nevertheless, despite the fundamental differences in, e.g., how the mean and variability of responses are coupled in these two classes of models, no prior work attempted to link either of them directly to the rich structure of neural variability in sensory cortices.

We have shown here that a sampling-based representation correctly predicted that particular stimulus manipulations result in systematic, mean-independent modulations of variability in V1. Further analysis also revealed that these modulations of variability in the model, though they sometimes appeared to be subtle, in fact conveyed substantial amounts of information about the stimulus and thus could be expected to be functionally relevant for downstream computations (Supplemental Experimental Procedures; Figure S5). Crucially, models that couple response means and variances cannot capture these effects (Ma et al., 2006). Moreover, sampling also provided a parsimonious account of the similarity of noise, signal, and spontaneous correlations, as well as the similarity between evoked and spontaneous activities, which do not naturally emerge without additional assumptions in alternative models of probabilistic representations (Deneve, 2008, Ma et al., 2006, Rao, 2004, Zemel et al., 1998).

### Key Model Assumptions

Our results were obtained by representing the result of inference over variables encoding basis function activations (Equation 1), and not those that encode contrast (z in Equation 1). This choice can be justified in two ways, both of which have precedents in previous representational models of V1 (Berkes et al., 2009, Karklin and Lewicki, 2009, Schwartz and Simoncelli, 2001). First, although such contrast variables are part of the generative model of natural images we considered, this does not imply that they also need to be explicitly included in the “recognition” model that the cortex uses to invert the generative model. Instead, they may be implicitly integrated out during inference. Note that even the posterior over basis function activations shows strong contrast dependence (both in its mean and covariance); therefore, without an explicit representation of the contrast variable, contrast can be decoded from population activity should this decoding be necessary. Second, statistical arguments suggest that the number of contrast-like variables needs to be far lower than the number of those representing basis function activations, and so the experimental recordings which we use to test the theory are likely to be largely probing the latter. Nevertheless, were contrast-like variables represented explicitly in V1 and identifiable in experimental recordings (perhaps in inhibitory interneurons), we predict that their activity during spontaneous activity should not reflect the prior and, consequently, also should not match their average evoked-activity distribution.

In line with previous approaches (Karklin and Lewicki, 2009, Olshausen and Field, 1996, Schwartz and Simoncelli, 2001), our model took the posterior to be static compared to the timescale of inference, although under natural conditions, the posterior distribution itself may be changing due to both bottom-up and top-down effects. Bottom-up-driven changes in the posterior occur because the visual stimulus is changing, while top-down factors include changes in attention, cortical state (Goris et al., 2014, Harris and Thiele, 2011), and interactions with other sensory modalities (Driver and Noesselt, 2008). Thus, our results apply to standard visual electrophysiological experiments in which these factors are either well-controlled, by using the same stimulus and ensuring a homogeneous attentional state across multiple trials (Ecker et al., 2010), or averaged out, by pooling data over long time windows (Berkes et al., 2011a, Fiser et al., 2004). Furthermore, because the synchronized cortical state is characterized by large-amplitude fluctuations in membrane potentials and overall activity of cortical neurons, which are generally hard to control, our predictions are most directly testable in the desynchronized state characteristic of cortical populations processing the attended stimulus (Harris and Thiele, 2011; see also Supplemental Experimental Procedures and Figure S4).

### Sampling and Neural Circuit Mechanisms

While our theory defines a neural representational scheme, it remains agnostic as to the neural circuit dynamics that give rise to such representations. As such, it accounts for the stationary distribution of neural network dynamics (as the posterior distribution that needs to be sampled) which is most readily testable in variability at slow timescales, e.g., across trials. However, anchoring the representation computationally in this way also provides useful constraints for mechanistic models that explicitly examine the underlying cellular- and network-level dynamics and thus make predictions about correlations at shorter timescales.

In particular, our model requires that the dynamically evolving membrane-potential or firing-rate traces of neurons represent sequences of stochastic samples from a posterior distribution. There have indeed been several neural circuit models proposed recently in which single neuron properties together with feedforward and recurrent connections shape either intrinsic or extrinsic noise in a network, such that for any particular input its dynamics produce samples from a computationally appropriate posterior distribution of activities (Buesing et al., 2011, Hennequin et al., 2014a, Savin et al., 2014). Such network models establish important proofs of the principle that neural circuit dynamics can give rise to sampling-based representations, and will be useful for making predictions about correlations on faster, within-trial timescales.

While the same stationary distribution can be attained by many different sampling algorithms, these will be different in their transient behaviors (so-called “burn-in”) and non-equilibrium properties (i.e., whether and how they violate detailed balance), and so data about autocorrelations, including characteristic oscillations, fast timescale cross-correlations, and transients (Azouz and Gray, 1999, Ray and Maunsell, 2010), should reveal hallmarks of the specific sampling dynamics employed by the cortex (Hennequin et al., 2014a). For example, our preliminary results indicate that the stimulus-onset-related transients and the contrast-dependent oscillation frequency of V1 responses may be accounted for by a specific class of sampling-based neural circuit dynamics that is both computationally efficient and neurally plausible (Aitchison and Lengyel, 2014), in that it accommodates separate classes of excitatory and inhibitory neurons which most previous approaches eschewed (Buesing et al., 2011, Savin et al., 2014).

### Sampling in Hierarchical Systems

Sampling-based representations lend themselves particularly naturally to self-consistent computations across multiple layers of a processing hierarchy ranging from low-level to high-level visual features, such as those found along the visual pathway (Lee and Mumford, 2003, Salakhutdinov and Hinton, 2012). Relating sampling in such hierarchically organized systems to neural variability along the cortical hierarchy should be able to capture various top-down effects in sensory processing that our simplified, non-hierarchical model could not address (Cohen and Maunsell, 2009, Kohn et al., 2009, Roelfsema et al., 2004). Indeed, recent results indicate that such a hierarchical sampling model can account for a variety of top-down task-related effects in visual cortical areas (Haefner et al., 2016). Moreover, our derivations for such a hierarchical extension not only reproduce all the main results of our simpler model, but they also predict that even images with equal contrast can evoke different amounts of response variability at both high and low levels of the hierarchy, depending on whether they afford higher-order percepts (Supplemental Experimental Procedures; Table S2). This is in line with recent experimental data comparing the sparseness and reliability of V1 responses to natural and phase-scrambled images (Froudarakis et al., 2014).

Note that hierarchical inference also obviates the need for an explicit, direct decoding of the posterior distribution from the samples, e.g., in the form of a histogram, as decision variables can be simultaneously inferred (and sampled from) together with lower-level variables. Moreover, both decision making and learning only require posteriors indirectly, through integrals of a cost function (Dayan and Abbott, 2005), thus implicitly implying a “smoothing” of samples. This smoothing mitigates the effects of the idiosyncratic “spiky” or “spaghetti-like” shape of sampling-based representations (e.g., in Figure 1C). More generally, non-linear effects in hierarchical inference can also explain how a relatively modest (less than a factor of 2) reduction in variance at the level of V1 following stimulus onset (Figure 3) can be compatible with a drastic, orders-of-magnitude change in perceptual confidence (Supplemental Experimental Procedures; Figures S3E and S3F).

### Sampling through Time

As inferences in our model are represented by sequentially generated samples at the rate of one new statistically independent sample every few tens (for membrane potentials) or hundreds of milliseconds (for spike counts), we expect this to limit the resolution of the representation of uncertainty. (Although, by using over-complete representations, in which many neurons effectively code for the same variable, even one sample of a population activity pattern may represent multiple samples of the relevant variables, such that the effective rate of sampling can be faster than expected from neural time constants; see, e.g., Savin and Deneve, 2014, and also Supplemental Experimental Procedures.) Indeed, such a gradual buildup of the representation of uncertainty over time within individual trials has been recently described (Lengyel et al., 2015). Moreover, it has been suggested that human-level performance in a range of behavioral tasks is indeed achievable by collecting a limited number of samples from a probability distribution given either static (P. Berkes et al., 2011b, COSYNE, conference; Vul et al., 2009) or dynamic stimuli (Levy et al., 2009). It has also been shown that specific patterns of perceptual variability in bi-stable percepts can be directly accounted for by sampling-based dynamics (Moreno-Bote et al., 2011). Our work complements these behavioral results by identifying the neural signatures of a sampling-based representation in V1, and demonstrates that the structure of neural variability and covariability provides useful clues for understanding the underlying probabilistic computations and representations utilized by the cortex.

## Experimental Procedures

### The Gaussian Scale Mixture Model

We used a Gaussian scale mixture (GSM) model (Wainwright and Simoncelli, 2000) to define a generative model of image patches (Figure 1A). Each patch was represented by a vector of pixel values x and assumed to be generated by a scaled, linear combination of features plus additive Gaussian white noise (see also Equation 1),

(Equation 2) $$\text{P}\left(x|y,z\right)=N\left(x;z\;A\;y,\sigma_{x}^{2}\;I\right),$$

where y describes the activation of features in A for that image, z is an independent variable scaling the output of these features, and $\sigma_{x}^{2}$ is the variance of observation noise independently affecting the intensity of every pixel of the image. The multiplicative interaction between z and the basis functions captures two important aspects of natural images: first, that the effective contribution of each basis function (its activation level, y, multiplied by z) is sparsely distributed, and second, that the magnitude of basis-function contributions within the same local image patch tends to be correlated (Schwartz and Simoncelli, 2001).

The prior of activations was a multivariate normal distribution with a mean of zero and covariance matrix C,

(Equation 3) P(y)=N(y;0,C),

and the prior distribution of the scale variable, P(z), was a Gamma distribution with parameters k and θ.

The posterior distribution over feature activations could be obtained in a closed form for the scale variable z and, conditioning on z, also for the feature activations y,

(Equation 4) $$\text{P}\left(z|x\right)\propto \text{P}\left(z\right)N\left(x;0,z^{2}ACA^{\text{T}}+\sigma_{x}^{2}\;I\right)$$

and

(Equation 5) P(y|z,x)=N(y;μ(z,x),Σ(z)),

where the posterior mean and covariance of feature activations is

$$\Sigma \left(z\right)={\left(C^{-1}+\frac{z^{2}}{\sigma_{x}^{2}}A^{\text{T}}A\right)}^{-1}\;\text{and}\;\mu \left(z,x\right)=\frac{z}{\sigma_{x}^{2}}\Sigma \left(z\right)A^{\text{T}}\;x.$$

As it was not necessary to represent the posterior distribution of z explicitly, we marginalized over this variable in order to express P(y|x)=∫dzP(z|x)P(y|z,x).

Membrane potentials (dimensionless), u, were taken to represent a weakly non-linear function of visual feature activations y (Figure 1B, bottom):

(Equation 6) $$u_{i}=\text{sign}\left(y_{i}\right){\left|y_{i}\right|}^{\alpha }.$$

Firing rates were generated by first sampling membrane-potential values and then transforming them using a standard, rectified non-linearity (Carandini, 2004) (Figure 1B, middle):

(Equation 7) $$r_{i}=m{\left(u_{i}-u_{\text{thresh}}\right)}_{+}^{\beta }.$$

For sampling consecutive firing-rate values, we approximated autocorrelation timescales by regarding the firing rate of a cell to be constant within each 20 ms time bin and independently sampling across bins. Spike counts, **n**, were generated simply by integrating instantaneous firing rates over time, starting from a random value distributed uniformly between zero and one (Figure 1B, top). Spike counts were computed over trial durations that matched those used in the corresponding experiments.

See Supplemental Experimental Procedures for a justification of model choices and more details of the model, including the setting of parameters, criteria used to select relevant experimental data to test the model, and procedures for analyzing neural responses in the model and in experimental data. Code for the model is available at https://github.com/gergoorban/sampling_in_gsm.

## Author Contributions

M.L. conceived the theoretical framework. G.O. and M.L. developed the model and conducted the mathematical analyses. G.O. performed the numerical simulations. G.O., P.B., J.F., and M.L. discussed the results and wrote the manuscript.

## Supplemental Information

Document S1. Supplemental Experimental Procedures, Figures S1–S6, and Tables S1 and S2

Document S2. Article plus Supplemental Information
