# Supplementary material for: Neural Variability and Sampling-Based Probabilistic Representations in the Visual Cortex
Source: Neuron. 2016 Oct 19;92(2):530–43. doi: 10.1016/j.neuron.2016.09.038 (PMC5077700; doi:10.1016/j.neuron.2016.09.038)
Supplement: Document S1. Supplemental Experimental Procedures, Figures S1–S6, and Tables S1 and S2 [file mmc1.pdf]

Neuron, Volume *92*

Supplemental Information

Neural Variability and Sampling-Based Probabilistic Representations in the Visual Cortex

Gergő Orbán, Pietro Berkes, József Fiser, and Máté Lengyel

Neural variability and sampling-based probabilistic representations in the visual cortex
# – Supplemental Experimental Procedures –

Gergő Orbán, Pietro Berkes, József Fiser, Máté Lengyel

Contents

1 Model details 2
2 Data set selection criteria 5
3 Statistical measures of neural activity 7
4 Change of variables: membrane potential- and firing rate-based representations 12
5 Relation to earlier representational models and reproducing data about average responses 13
6 Establishing relationships between different forms of correlations . 17
7 Hierarchical inference and top-down influences on response variability in V1 21
8 The relevance of stimulus-dependent changes in neural variability: decoding stimuli from spike trains 26

List of Figures

S1 Model parameters 37
S2 Contrast-invariance and non-classical receptive field effects in a representative model neuron 39
S3 Response variability in the model: parameter dependence and implications for perceptual confidence 41
S4 Response correlations 43
S5 Decoding performance of a linear and an optimal decoder 44
S6 Match between spontaneous (SA) and average evoked activity (aEA) distributions in the model depends on correlations 45

1 Model details

Gaussian scale mixture model. The Gaussian scale mixture model is defined in Eqs. 2-5 in Experimental Procedures. We have chosen this widely used model as our starting point not only because its variants capture essential aspects of the statistics of natural images and thus demonstrated cutting-edge performance in image compression and denoising (Wainwright & Simoncelli, 2000; Portilla & Simoncelli, 2000), but also because it has provided a normative account of both electrophysiological data about average firing rates in V1 (Olshausen & Field, 1996; Schwartz & Simoncelli, 2001), as well as about behavioral data on low-level vision (e.g. the tilt illusion; Schwartz et al. (2009)). While the true generative process of natural images, including high-level objects and attributes, is certainly more complex than the GSM model we are using here, the representation of these features also goes well beyond the level of V1. Therefore, we regard the GSM as an appropriate model to investigate stimulus-related changes in the distribution of V1 responses as long as the stimulus manipulations are well captured by the low-level visual features represented in V1.

In order to obtain a posterior that is not dependent on z, marginalization was performed by integrating over the posterior distribution of z, $\mathrm{P}(\mathbf{y}|\mathbf{x}) = \int \mathrm{P}(\mathbf{y}|z, \mathbf{x})$ $\mathrm{P}(z|\mathbf{x})\,\mathrm{d}z$ (with $\mathrm{P}(\mathbf{y}|z, \mathbf{x})$ given by Eq. 5 in Experimental Procedures). Marginalization was approximated as follows: a range of z around the maximum a *posteriori* (MAP) estimate was discretized and the posterior at the discretized values of was evaluated, thus the posterior of $\mathbf{y}$ effectively became a mixture of a finite number of Gaussians. The number of mixture elements was 50 during learning and 1 for inference, with the MAP value used for z. Using a mixture instead of the MAP value for inference did not qualitatively affect our results. Due to the lack of top-down influences in our underlying GSM model (see above), the posteriors we computed did not express higher-level forms of uncertainty, e.g. resulting from bistable percepts or ambiguity at the object-level. Nevertheless, the model still captured one of the most important forms of ambiguity at the level of V1: a certain local contrast level in the image was congruent with a continuum of hypotheses about (localized) basis function activations and global contrast level. As we show in the Results, this fundamental source of uncertainty has direct consequences on the forms of neural variability that are implied by a sampling-based representation of uncertainty.

Neural responses in the model. Membrane potentials, $\mathbf{u}$, were obtained by a weakly nonlinear transformation from feature activations, $\mathbf{y}$ (Eq. 6 in Experimental Procedures). This can also be understood as a generative model in which the latent variables are the membrane potentials, $\mathbf{u}$, themselves, but their prior is not a multivariate Gaussian anymore, as for $\mathbf{y}$ (Eq. 3 in Experimental Procedures), but instead a multivariate Gaussian mapped through this nonlinearity (see also Section 4). In contrast to the non-kurtotic normal prior distribution of $\mathbf{y}$, the prior of $\mathbf{u}$ becomes sparse at α values larger than 1 (**Fig. S3**).

Membrane potentials were generated by sampling $\mathbf{y}$ directly from the posterior (see above) and transforming them into samples of by using Eq. 6 in Experimental Procedures (**Fig. 1B**). This way, we did not address the question of how the dynamics of a neural circuit would obtain samples from $\mathbf{u}$ (but see Büsing et al., 2011; Grabska-Barwinska et al., 2013; Hennequin et al., 2014; Savin et al., 2014). Although, in general, samples generated by such dynamics will be correlated, samples separated by intervals beyond the time scale over which autocorrelations decay (20-50 ms for membrane potentials, Azouz & Gray (1999), and spike trains, Berkes et al. (2011b), in V1) will effectively be uncorrelated. Therefore, for membrane potentials, we compared our results, based on obtaining independent samples from the posterior, to data describing variability across trials which is well beyond the decorrelation time scale. In practice, the statistics of membrane potential responses (their mean, variance, and covariance) were computed by performing the corresponding integrals of Eq. 6 in Experimental Procedures over the posterior of $\mathbf{y}$ using numerical quadrature rather than using the samples to compute Monte Carlo integrals.

Spike counts were obtained by feeding membrane potentials through the firing rate nonlinearity (Eq. 7 in Experimental Procedures) and integrating these instantaneous firing rates over time and finally generating spikes at integer values of the integral. This corresponded to a simple deterministic (non-leaky integrate and fire) spike generation process in which a spike is taken to be emitted deterministically whenever the integrated input reaches a fix threshold (**Fig. 1B**). (Note that only spike counts and not the exact timing of spikes were analyzed in this study.) This procedure can be shown to generate spikes with exactly the mean firing rate given by r, such that spike count variability is determined almost entirely by firing rate variability (with minimal additional variability due to randomness in the initial condition). This is in contrast to an inhomogeneous Poisson process, which

would add a substantial amount of spiking noise, increasing Fano factors by one on top of the variability due to variations in firing rates.

Model parameters and inputs. All results were obtained with a single set of parameters (except where explicitly noted to demonstrate robustness to changes in parameters, **Figs. S2-S3**). For the GSM, we obtained the variance of the observation noise, σ_{x}^2 (Eq. 1), and the prior covariance matrix of visual features, $\mathbf{C}$ (Eq. 3 in Experimental Procedures), by fitting the model to pseudo-whitened 16-by-16 pixel natural image patches (van Hateren, 1992) using the expectation-maximisation (EM) algorithm (Dempster et al., 1977) (**Fig. S1B**). The E-step of the algorithm consisted of computing the posterior distributions as described above, while the M-step was subdivided into three partial alternating steps each changing one (set of) parameter(s) of the model: σ_{x}^2, and the norm and direction of $\mathbf{C}$. The rest of the parameters were fixed at preset values: $k = 2$ and $\theta = 2$ for the prior of the scale variable z (the specific values of these parameters did not affect our results); and the matrix of features, $\mathbf{A}$ (Eq. 2 in Experimental Procedures), was a bank of Gabor filters (Schwartz & Simoncelli, 2001) generated using four orientations and four spatial frequencies, each with a size of 1.9 times the length scale and at 1, 9, 16, or 36 different spatial locations (for increasing spatial frequency), so that a nearly complete (dimensionality: 248) basis set was formed (**Fig. S1A**). (Maximum likelihood learning of $\mathbf{A}$ simultaneously with $\mathbf{C}$ would be underconstrained because the predictive density only depends on their product $\mathbf{A}\mathbf{C}\mathbf{A}^{\mathsf{T}}$.) The best-fit value of σ_{x}^2 (noise), relative to the product of the average of the diagonal elements of the matrix $\mathbf{A}\mathbf{C}\mathbf{A}^{\mathsf{T}}$ and the expected value of z^2 (signal), was such that the model attributed a 1.8 signal-to-noise ratio to the training images.

The four remaining parameters of the model, determining the mapping of latent variables $\mathbf{y}$ to biological quantities (membrane potentials, firing rates, and spike counts), were determined based on experimental data. The exponent of the membrane potential nonlinearity (Eq. 6 in Experimental Procedures) was fitted to the orientation dependence of membrane potential variability (Finn et al., 2007) ($\alpha = 1.4$, see also Section 4). Following Carandini (2004), the exponent of the firing rate nonlinearity (Eq. 7 in Experimental Procedures) was set to be $\beta = 1.1$. As changes of the other two parameters of the firing rate nonlinearity, m and u_{thresh} (Eq. 7 in Experimental Procedures), could be compensated for by appropriate changes in the prior mean of $\mathbf{u}$ and the scale of $\mathbf{A}$, once those parameters were set (see above), we used these two parameters to fit the physiological range

of firing rate data: $m = 10\,\mathrm{Hz}$ and $u_{\mathrm{thresh}} = 1.9$. This both provided physiological firing rates (Carandini, 2004) in the model and set the level of spike count Fano factors (**Fig. 3D**, top) and variability (**Fig. 4D**, top) such that they matched experimentally measured levels (**Fig. 3C**, bottom, **4D**, bottom, see also **Fig. S3D**). (Note that no spike count correlation data was considered for these fits.)

Stimuli used to investigate response statistics of neurons in the simulations were chosen to match the statistics of stimuli used in the corresponding experiments. More specifically, the generation of natural image patches was identical to that used for generating training images (see above) (**Figs. 2, 5, 6, S6**). Grating images used either static (**Figs. 3E, 4B,D,E, 6** insets, **6b, S4C-E**) or drifting full-field sinusoidal gratings (**Figs. 3A-D, 4A,C, 6, S2, S3**) at 12 different orientations and 12 different phases. Random noise images were 2×2 binary block noise image patches (**Figs. 7B, S6**). Following Haider et al. (2010), natural image movie sequences (**Fig. 5**) were created by sampling a larger natural image along a linear trajectory. CRF and nCRF stimulation corresponded respectively to stimulating only the central 11 pixel-diameter part of the input, or all 16-by-16 pixels. In all these cases we simply modelled responses based on independent samples for each 20 ms time bin conditioned on the corresponding snapshot of the stimulus.

2 Data set selection criteria

We selected the experimental data sets we modeled based on two sets of criteria.

Neural data. Whenever possible, we used data sets in which membrane potentials were (also) recorded with their variance (or, as a proxy, their reliability) quantified, because these provided the most direct test of the theory. However, this had the disadvantage of almost always being recorded in anesthetised animals in which variability can show different patterns from awake animals (Ecker et al., 2014, see also below), and that with very few exceptions only one neuron is recorded at a time thus preventing the measurement of noise covariability. Therefore, we used spike count data recorded in the awake animal to demonstrate that the main results are also borne out in the awake state and specifically to analyse data on covariability which is particularly sensitive to effects of anesthesia (see below). For this, we compared to previous work in which changes in spike count variability – our main

interest (eg. change of Fano factor with contrast) – were analysed (Churchland et al., 2010). As most other published papers on spike counts did not perform such analyses, we also included previous work for which the original data was made publicly available (Ecker et al., 2010) or was our own (Berkes et al., 2011a) so that we could perform these analyses ourselves.

Stimulus manipulations. We chose data sets in which stimulus manipulations relevant for testing the main predictions of the model were carried out: orientation, contrast, aperture, natural vs. artificial stimulus ensembles. Wherever possible, we used data obtained with static stimuli because the generative process (the Gaussian scale mixture, GSM) underlying our model was defined for such inputs (although it is certainly possible to extend it to dynamic inputs). We made exceptions and used dynamic stimuli when such data were not available (membrane potential recordings and experiments investigating the effect of aperture), and for analyses involving signal correlations to improve the quality of their estimation (see Section 3).

Based on these criteria, the data sets used in the studies listed in Table S1 were the only appropriate ones.

Excluding anesthetised data from the analysis of correlations

For comparing correlations in our model to experimental data, we used only data recorded in awake animals. This is because commonly used forms of anesthesia can introduce coordinated, stimulus-independent fluctuations in the responses of cortical populations which can inflate both variances and correlations of neural responses (Ecker et al., 2014; Goris et al., 2014). Despite the effects of these fluctuations on the absolute magnitude of single neuron variability, as we show below they are not expected to qualitatively affect relative stimulus-dependent changes in variability, ie. increasing contrast should still decrease variance (**Fig. S4A**). Thus, it was possible to include anesthetized data for analyzing stimulus-dependent changes in variances and Fano factors in the preceding sections. Indeed, all our main predictions regarding single neuron variability were confirmed not only by anesthetized but also by awake recordings (see **Figs. 3-5** of the main text). In contrast, we show below that the same fluctuations, depending on their relative magnitude, can have more complex effects on changes in correlations (Goris et al.,

2014) whereby they can even revert the direction of correlation changes that would be seen in the awake animal in the absence of these fluctuations (**Fig. S4B**). For these reasons, we excluded anesthetized data from the analyses of correlations and joint pattern statistics (**Figs. 6-7** of the main text).

In order to estimate the effect of synchronized activity fluctuations on membrane potential variance and correlations, we constructed a simple model that describes the membrane potential of a pair of neurons as the sum of two factors: a baseline that is identical and shared across the two neurons and undergoes fluctuations with variance ω^2, and a neuron-specific 'private' component which has variance σ^2 in both neurons and (noise) correlation ρ across the two neurons. (A similar additive interaction between population-wide shared and single neuron-specific private variability has been found to provide a good fit to the overall variability in firing rates of V1 neurons in the awake macaque; Ecker et al., 2014.) With these assumptions, the overall variance of neural activities is the sum of the variances of the two contributing factors:

$$\tilde{\sigma}^2 = \sigma^2 + \omega^2 \tag{S1}$$

and the overall correlation between the two neurons, which includes the effects of synchronized baseline fluctuations, will be:

$$\tilde{\rho} = \frac{\rho\,\sigma^2 + \omega^2}{\sigma^2 + \omega^2} \tag{S2}$$

To see how our model's predictions transfer to this case, we consider that increasing contrast decreases private variance, σ^2, and the underlying correlation, ρ, but does not affect shared variance, ω^2. In this case, it is easy to see that single neuron variability, $\tilde{\sigma}^2$, will decrease irrespective of the magnitude of shared variance (**Fig. S4A**), ie. our main prediction carries over. In contrast, pairwise correlations, $\tilde{\rho}$, may decrease or increase, depending on the interaction of three factors: the magnitude of private variance reduction, correlation reduction, and the shared noise variance (**Fig. S4B**).

3 Statistical measures of neural activity

In order to ensure a fair comparison, responses generated by the model were analyzed in the same way as the corresponding experimental data (either already

published or our own analysis see below). The size of the model network was chosen based on computational considerations. For panels showing analysis of model results either all cells from the network were used, or cells were included following the same criteria as those used for the inclusion of cells in the corresponding experiments. Number of “animals” in **Fig. 7** were the same as in the corresponding experiments (Berkes et al., 2011a). Statistical tests used are accepted as standard for the given purposes and were the same as those used in the experiments (wherever applicable). We used parametric tests wherever sample size justified such a choice, otherwise the appropriate non-parametric tests were chosen. Tests with equal or unequal variance assumption were used depending on the characteristics of the data. All tests reported are two-tailed.

Fano factor analysis was constrained to neurons whose spike counts exceeded 0 in the analyzed stimulus conditions. Comparison of Fano factors between spontaneous and evoked activity, or across different contrast levels (**Figs. 3D-E** and **4B**) was based on mean-matched Fano factors computed following the methods in Churchland et al. (2010). Specifically, each neuron contributed one datum (point on a spike count variance vs. mean scatter plot) for each stimulus orientation, the ”population” of these data points was subsampled such that the distribution of spike count means across the population was matched under the different conditions being compared, and then the Fano factor in each condition was measured as the slope of the regression line relating spike count variances to means in the subsampled population. This regression was weighted by (the inverse of) the variance of the spike count variance estimate of each datum. This procedure ensured that differences in Fano factors could not be attributed simply to changes in spike count means, or a reduction in spiking process noise due to the spike train-regularizing effect of higher mean rates, and instead reflected changes in the variability of underlying firing rates. For comparing Fano factors across orientations (**Fig. 4E**) no mean matching was used as it would have reduced the number of trials too drastically (by requiring a match of spike count mean distributions across all orientations) and because no mean matching made it easier to reject the null hypothesis that there was no difference in Fano factors (which in this case we sought to confirm). For all comparisons, the significance of the difference of Fano factors was computed based on the sampling distributions implied by the 95% confidence intervals of the regression yielding the Fano factors.

Following Vinje & Gallant (2000) and Haider et al. (2010), lifetime sparseness (**Fig. 5B**) was computed as

$$S = 1 - \frac{\left(\sum_i r_i/N\right)^2}{\sum_i r_i^2/N} \tag{S3}$$

where r_i was the firing rate of a neuron averaged across 500 trials for image frame i, and $N = 100$ was the number of frames. Sparseness analysis was constrained to neurons whose receptive fields overlapped with the smaller aperture in which the stimulus was presented (n=54). Reliability of membrane potential responses for the same set of neurons (**Fig. 5C**) was assessed by calculating the cross correlation of membrane potential sequences in each of 124,750 possible pairs of 500 trials and averaging this measure across trial pairs.

Decorrelation of population responses (**Fig. 5D**) was assessed as in Vinje & Gallant (2000). We calculated peri-stimulus time histograms (PSTHs) for neurons for 100 20-ms frames of a natural image sequence, and represented each PSTH as a vector with 100 elements. Dissimilarity of the responses of a pair of neurons was then quantified as the separation angle between their PSTH vectors in this 100-dimensional space:

$$\vartheta_{ij} = \arccos \frac{\mathbf{PSTH}_i^{\mathsf{T}}\,\mathbf{PSTH}_j}{||\mathbf{PSTH}_i||\,||\mathbf{PSTH}_j||} \tag{S4}$$

The distribution over separation angles provided a measure of signal correlations in the population response under different conditions (lower angles corresponding to higher correlations).

Noise and spontaneous correlations were calculated from z-scored spike counts in a 400-ms window. The time scale of obtaining independent samples from the posterior (20 ms in our simulations) might seem to provide a more natural time scale for computing correlations but measurement noise due to very low spike counts dominates for such short time windows and so we used longer time windows that were also compatible with those used in previously published experimental work. Trials with an absolute z-score larger than 3 were discarded from the analysis. For a cell pair to be included in the analysis, the mean firing rate (across all orientations) of both neurons needed to exceed a threshold of 0.5 Hz. As we wanted to avoid the confounding effects of low firing rates on noise correlations (de La Rocha et al., 2007), we only included trials at the stimulus orientation

for which the pair had maximal geometric mean firing rate. Signal correlations were also analyzed in 400-ms windows, excluding trials with an absolute z-score greater than 3, and cell pairs in which the mean firing rate of both cells did not exceed 0.5 Hz.

For the analysis of Kullback-Leibler (KL) divergence (**Fig. 7**), as the experimental data consisted of multiunit spike trains (Berkes et al., 2011a), we created 16 multiunit spike trains by combining the spike trains of 4 randomly selected (without replacement) model neurons for each multiunit. (Similar results were obtained by using 16 randomly selected individual model neurons for the analysis, see **Fig. S6**.) Different animals were simulated by randomizing over the assignment of model neurons to multiunits (n=20), and drawing a different random sample of membrane potentials (and hence spike counts).

KL divergence was computed between the average evoked and spontaneous activity distribution following the equation:

$$\mathrm{KL}\left[\mathrm{aEA}\,||\,\mathrm{SA}\right] = \sum_{\mathbf{n}_{0/1}} \mathrm{P}_{\mathrm{aEA}}\big(\mathbf{n}_{0/1}\big) \log_2 \frac{\mathrm{P}_{\mathrm{aEA}}\big(\mathbf{n}_{0/1}\big)}{\mathrm{P}_{\mathrm{SA}}\big(\mathbf{n}_{0/1}\big)} \tag{S5}$$

where $\mathbf{n}_{0/1}$ is the population spike count vector of the multiunits binarized in 2 ms time bins. The average evoked activity distribution

$$\mathrm{P}_{\mathrm{aEA}}\big(\mathbf{n}_{0/1}\big) = \int \mathrm{P}\big(\mathbf{n}_{0/1}|\mathbf{x}\big)\ \mathrm{P}(\mathbf{x})\,\mathrm{d}\mathbf{x} \tag{S6}$$

is the distribution of responses given to stimuli coming from a particular stimulus statistics, $\mathrm{P}(\mathbf{x})$. The spontaneous activity distribution $\mathrm{P}_{\mathrm{SA}}\big(\mathbf{n}_{0/1}\big)$ was based on the prior of visual features, $\mathrm{P}(\mathbf{y})$ (Eq. 3 in Experimental Procedures), or equivalently, using responses to a blank stimulus, $\mathrm{P}\big(\mathbf{n}_{0/1}|\mathbf{x}=0\big)$ (see **Fig. 2**).

In practice $\mathrm{P}_{\mathrm{aEA}}\big(\mathbf{n}_{0/1}\big)$, (Eq. S6) was computed as a histogram of responses, by sampling 600 stimuli from $\mathrm{P}(\mathbf{x})$ (i.e. randomly selected natural image patches or gratings) and for each stimulus sampling 1200 responses from the response distribution of the model, $\mathrm{P}\big(\mathbf{n}_{0/1}|\mathbf{x}\big)$ (Eqs. 5-7 in Experimental Procedures, followed by binarization), yielding a total of 720,000 sample responses. Similarly, $\mathrm{P}_{\mathrm{SA}}\big(\mathbf{n}_{0/1}\big)$ was also computed as a histogram, using the same number of sample responses. The KL divergence between the two response histograms (Eq. S5) was estimated using the methods described in Berkes et al. (2011a).

Analysis of neural variability in awake monkey recordings. In short, single unit recordings from V1 neurons were analyzed in response to static gratings with 8 orientations and two contrast levels, or moving grating images with 16 directions and a single contrast level (Ecker et al., 2010). Data were stored as spike counts in 10-ms time bins. According to the standards of the original analysis methods, only units with low contaminations (<0.05) were used in all our analyses.

For the analyses of the stimulus-dependence of neural variability (Fano factors: **Figs. 3E**, and **4B,E**), we used the static grating data set because it included several contrast levels (see above). We analyzed 100-ms segments of the neural response, preceding or following stimulus onset by 50 ms respectively for spontaneous and evoked activity. (For measuring correlations, 400-ms segments were used, see also above.) In this data set, each recording session, yielding 7-22 units, included a lower and a higher contrast condition, but the actual contrast levels in these conditions varied across sessions. To increase statistical power, we pooled data across sessions and split the data into low and high contrast conditions at a contrast level of 20% (which resulted in approximately the same number of sessions being analyzed in the two contrast conditions).

For the analysis of the orientation-dependence of Fano factors (**Fig. 4E**), the preferred orientation of units was determined by fitting Gaussian tuning curves to their orientation-dependent firing rates during stimulus presentation in a 400-ms window. The maximum of the fitted tuning curve was used as the preferred orientation of each cell and stimulus orientations were measured relative to this preferred orientation.

For the analyses involving signal correlations (**Fig. 6**) we used the moving gratings data set to compute all three types of correlations (signal, noise, and spontaneous) as this data set included more orientations and thus a more robust measure of signal correlations, and also because it yielded higher average firing rates and thus a smaller potential confounding effect of low firing rates (see also above).

All other steps of the analyses proceeded as described in the previous section to ensure uniformity of analysis for model and experimental data.

4 Change of variables: membrane potential- and firing rate-based representations

We have chosen to define the GSM in terms of abstract latent variables (representing basis function activations), $\mathbf{u}$, and then to obtain membrane potentials $\mathbf{u}$ as representing nonlinearly warped versions of $\mathbf{y}$, $u_i = f_\mathrm{u}(y_i)$, and firing rates $\mathbf{r}$ through a further rectified nonlinear mapping of $\mathbf{u}$, $r_i = f_\mathrm{r}(u_i)$ (Eqs. 6-7 in Experimental Procedures and **Fig. 1B**). The question may arise as to why we did not choose instead a generative model in which membrane potentials or instantaneous firing rates directly represent latent variables? Our choice was motivated mainly by mathematical convenience: this way, the prior distribution of our latent variables could be kept a simple multivariate normal (Eq. 3 in Experimental Procedures), while neither membrane potentials (see below) nor firing rates are normally distributed *in vivo*. Nevertheless, note that our formulation of the model is formally strictly equivalent to a model in which membrane potentials or instantaneous firing rates represent latent variables directly. In that model, the prior and the likelihood are simply the prior and the likelihood used in our model ($\mathrm{P}(\mathbf{y})$ and $\mathrm{P}(\mathbf{x}|\mathbf{y}, z)$, Eqs. 2-3 in Experimental Procedures) appropriately transformed through f_u (and f_r):

$$\mathrm{P}(\mathbf{u}) = \int \delta(\mathbf{u} - \mathbf{f}_\mathrm{u}(\mathbf{y}))\ \mathrm{P}(\mathbf{y})\, \mathrm{d}\mathbf{y} \tag{S7}$$

$$\mathrm{P}(\mathbf{x}|\mathbf{u}, z) = \frac{1}{\mathrm{P}(\mathbf{u})} \int \delta(\mathbf{u} - \mathbf{f}_\mathrm{u}(\mathbf{y}))\ \mathrm{P}(\mathbf{x}|\mathbf{y}, z)\ \mathrm{P}(\mathbf{y})\, \mathrm{d}\mathbf{y} \tag{S8}$$

or

$$\mathrm{P}(\mathbf{r}) = \int \delta(\mathbf{r} - \mathbf{f}_\mathrm{r}(\mathbf{f}_\mathrm{u}(\mathbf{y})))\ \mathrm{P}(\mathbf{y})\, \mathrm{d}\mathbf{y} \tag{S9}$$

$$\mathrm{P}(\mathbf{x}|\mathbf{r}, z) = \frac{1}{\mathrm{P}(\mathbf{r})} \int \delta(\mathbf{r} - \mathbf{f}_\mathrm{r}(\mathbf{f}_\mathrm{u}(\mathbf{y})))\ \mathrm{P}(\mathbf{x}|\mathbf{y}, z)\ \mathrm{P}(\mathbf{y})\, \mathrm{d}\mathbf{y} \tag{S10}$$

It is easy to see that both the predictive distribution over images, $\mathrm{P}(\mathbf{x})$, and the posterior distributions, $\mathrm{P}(\mathbf{u}|\mathbf{x})$ or $\mathrm{P}(\mathbf{r}|\mathbf{x})$, in that model are equivalent to those in our version. In other words, both the statistical justification (an internal model of natural image statistics) and the biological predictions (for neural responses) of our model remain the same. Including a separate membrane potential and firing rate step in our model also had the advantage that we could simultaneously test our model with both kinds of data.

Sparsely distributed membrane potential activations and the dependence of variability on orientation

One particular consequence of the non-linear mapping from $\mathbf{y}$ to $\mathbf{u}$ is a difference in their prior distributions (see also above). Specifically, the membrane potential of neurons in sensory cortices during spontaneous activity (corresponding to the prior, see the main text) has often been described as sparsely distributed (DeWeese & Zador, 2006; Okun et al., 2009). In contrast, the Gaussian scale mixture (GSM) model used in our work defines a normally distributed prior for the basis function activations which is characterized by zero sparseness. Thus, we used a weak power-law nonlinearity (**Fig. S3A**) to obtain membrane potential responses from basis function activations (Eq. 6 in Experimental Procedures) so that the membrane potential distribution under spontaneous activity became weakly kurtotic (**Fig. S3B**, excess kurtosis $\simeq 2.1$).

The same power-law nonlinearity also has another consequence: it amplifies high-amplitude membrane potential responses in both their mean and variance. This is because both the nonlinearity itself and its derivative are monotonically increasing (in absolute value) as the basis function activation is increasingly offset from zero. Therefore, this nonlinearity also predicts, in line with experimental data (Finn et al., 2007), that the variance of membrane potential responses to stimuli with preferred orientations will be proportionately slightly greater than those evoked by non-preferred stimuli (**Fig. 4C** of the main text). Higher membrane potential variance for low contrast stimulus was consistent across different levels of α and the orientation-dependence of the variance became more pronounced with higher levels of the parameter (**Fig. S3C**).

5 Relation to earlier representational models and reproducing data about average responses

Most previous representational models of V1 did not consider representing the full posterior, and instead proposed that neural activities encode a single combination of features (Olshausen & Field, 1996; Schwartz & Simoncelli, 2001; Karklin & Lewicki, 2009; Rao & Ballard, 1999). This point estimate can often be shown to correspond to the maximum probability under the posterior (*maximum a posteriori*, MAP) of a corresponding generative model (some variant of the GSM,

Richard Turner, personal communication, see also Olshausen, 1996). Thus for any input, these models assigned a single deterministic response which were taken as modelling the average response of the neuron to that stimulus as determined by its tuning curve and other receptive field properties. Indeed, these models showed prominent success at accounting for simple and complex cell receptive fields and tuning curves (Olshausen & Field, 1996; Schwartz & Simoncelli, 2001; Karklin & Lewicki, 2009).

In contrast, neural activities in our model represented uncertainty by encoding randomly sampled feature combinations under the posterior distribution. In turn, we used mean responses in our model, representing the mean of the posterior, to map receptive field properties (in close analogy to data analysis methods applied to experimental data). Nevertheless, for a reasonably unimodal and symmetric posterior distribution, the mean of the posterior will be close to its maximum. Therefore, as expected based on the formal relationship between the GSM and the generative models underlying previous work, our model replicated the success of earlier models at accounting for the classical and non-classical receptive field properties of V1 simple cells (**Fig. S2**). In particular, as the linear bases of the GSM consisted of oriented Gabor filters, neurons in our model showed clear orientation-tuning (see also section on *Key features* in the main text). Furthermore, the width of these orientation tuning curves was also contrast-invariant, and the cells also showed prominent non-classical receptive field effects, as expected based on earlier results (Schwartz & Simoncelli, 2001; Anderson et al., 2000; Finn et al., 2007), and as we explain below.

Contrast-invariant tuning

Both the membrane potential (especially at higher contrast levels) and the firing rate tuning curves of our model neurons showed contrast-invariant width (**Fig. S2A-B**). The former can be understood as a consequence of membrane potential responses in our model being a probabilistic interpretation of the model of Schwartz & Simoncelli (2001) – in which neural responses can be shown to correspond to the posterior mode (or mean, see above) of a GSM, at least approximately. Neural responses in Schwartz & Simoncelli (2001) show contrast invariant tuning curves due to divisive normalisation by a term that depends (quadratically) on a linearly filtered version of the input. Similarly, the posterior mean in our

model (Eq. 5 in Experimental Procedures) can approximately (at high contrasts) be written as $\boldsymbol{\mu} \simeq \mathbf{A}^{+}\,\mathbf{x}/\bar{z}$, where $\mathbf{A}^{+} = \left(\mathbf{A}\,\mathbf{A}^{\mathsf{T}}\right)^{-1}\mathbf{A}^{\mathsf{T}}$ is the (left) pseudo-inverse of $\mathbf{A}$, the bank of basis functions in our model, $\mathbf{x}$ is the input image, and $\bar{z} \propto \sqrt{\mathbf{x}\,\left(\mathbf{A}\,\mathbf{C}\,\mathbf{A}^{\mathsf{T}}\right)^{-1}\mathbf{x}^{\mathsf{T}}} \simeq \mathrm{argmax}_z\,\mathrm{P}(\mathbf{x}|z)$ is the (approximate) MAP estimate of the scaling variable, z, given the image (for the complete or overcomplete case). It is this division by $\bar{z}$, which itself depends (quadratically) on the input, that implements divisive normalization in our model, thus leading to the (approximate) contrast invariance of the posterior mean. As membrane potentials are directly related to $\mathbf{y}$ (of which $\boldsymbol{\mu}$ is the mean) through a weak nonlinearity (Eq. 6 in Experimental Procedures and **Fig. 1B**), this contrast-invariance is inherited by (average) membrane potential responses in our model (**Fig. S2A**, **C** gray line), in line with experimental data showing approximate contrast-invariance in the membrane potential responses of many V1 cells (Finn et al., 2007).

Importantly, even if membrane potential tuning curves had perfectly contrast-invariant width, this would not automatically result in contrast-invariant firing rate tuning curves, as the transformation of membrane potentials to firing rates involves rectification which could be expected to give rise to the “iceberg” effect, whereby an increasingly larger fraction of the membrane potential tuning curve exceeds the threshold of rectification as contrast is increased (Anderson et al., 2000). Thus, this “iceberg” effect would lead to a widening of the firing rate tuning curve and consequently a lack of contrast invariance (**Fig. S2C**, black line). This effect would be further exacerbated by the fact that membrane potential tuning curves themselves are already not perfectly contrast invariant and thus can show a slight increase in width with contrast (**Fig. S2C**, gray line, low contrasts).

The argument above for the iceberg effect assumed that the rectifying firing rate nonlinearity acted directly on the membrane potential tuning curves, which are defined by *trial-average* membrane potential responses. However, the firing rate nonlinearity acts on *instantaneous* membrane potentials (at a ~ 30 ms timescale, Priebe et al., 2004), which, crucially, are also affected by (noise) variability which in turn scales with contrast. This variability acts to restore the contrast invariance of firing rate tuning curves, as has been shown previously by Anderson et al. (2000) and Finn et al. (2007), whose data on membrane potential variability our model indeed reproduces (**Fig. 4** of the main text) and whose argument for contrast invariance we thus briefly recapitulate below (for more details see their papers).

Briefly, the crucial feature of contrast invariance is that suboptimally oriented stimuli at high contrast do not cause higher activity than an optimally oriented stimulus at low contrast. Crucially, in our model, membrane potential responses showed significant amounts of noise variability, and increasing stimulus contrast not only increased signal variability, but at the same time it also reduced this noise variability (**Fig. 2** of the main text, see also **Fig. S2A**, inset). This was in agreement with experimental data on the membrane potential statistics of V1 simple cells in the anesthetized cat (Finn et al., 2007).* These properties of noise variability prevented the iceberg effect in two ways. First, the additional variability smoothes out the "effective" firing rate nonlinearity, that maps from average membrane potentials to firing rates, such that its hard threshold disappears and so even stimuli for which the mean response falls below threshold can occasionally evoke spikes. Thus, simple additional variability, without contrast-dependence, will already restore contrast-invariance in firing rate tuning curves as long as membrane potential tuning curves themselves are contrast invariant (**Fig. S2C**, blue lines, high contrasts) (Anderson et al., 2000; Finn et al., 2007).

However, when membrane potential tuning curves were not contrast invariant (**Fig. S2C**, gray line, low contrasts), and in particular showed a contrast-dependent offset (**Fig. S2A**, low contrasts) this fixed amount of additional variability was insufficient to produce contrast invariance in firing rates (**Fig. S2C**, blue lines, low contrasts). In this case, the modulation of noise variability by contrast became important: the concomitant decrease in noise variance with increasing signal variance at higher contrasts meant that at suboptimal orientations a smaller fraction of the membrane potential distribution exceeded threshold, thus counteracting the iceberg effect and keeping the width of the tuning curves contrast invariant across all contrast levels (**Fig. S2B** and **C**, red line) (Finn et al., 2007). These effects were also robust to changes in the parameters of the firing rate nonlinearity within a reasonably broad range ($\pm$30-40%, including cases when the nonlinearity is sublinear rather than supralinear) (**Fig. S2D**-**E**). Therefore, despite the failure of previous attempts to reconcile sampling-based probabilistic representations with contrast invariant tuning curves (Pouget et al., 2013), our results indicate that contrast invariant tuning curves arise as a direct and robust consequence of the opposing

*Interestingly, these opposing changes in signal and noise variability with contrast were incompatible with a potentially simpler (linear) alternative account, according to which both signal and noise variability would originate from the same form of contrast-dependent variability in the input (Moreno-Bote et al., 2014). Such linear models can be extended to include normalization or adaptation, which could enable them to also exhibit mean-independent changes in noise variability.

changes in signal and noise variability of membrane potentials that are intrinsic to our model.

Non-classical receptive field effects

Nonlinear behavior of mean simple cell responses arises in the model when stimuli are presented in the nonclassical receptive field (nCRF) of the neuron (**Fig. S2F-H**). As we explain in the mean text, pixels that were not part of the "projective field" (Gabor basis function) that a neuron represented (the corresponding element of the $\mathbf{A}$ matrix was zero) could still influence the activity of this neuron due to the fundamental explaining away effect of probabilistic inference. There were two forms of explaining away in the model. First, there was explaining away via inferences in z (also explained in the main text, and illustrated in **Fig. 2** there, as well as above wrt. contrast invariance). Second, explaining away also took place via the posterior covariance matrix, $\mathbf{\Sigma}$, as the posterior mean was proportional to a product between the activities that would have resulted from a simple linear filtering of the image with the projective fields ($\mathbf{A}^{\mathsf{T}}\,\mathbf{x}$) and $\mathbf{\Sigma}$ (Eq. 5 in Experimental Procedures). Explaining away via z generally resulted in suppression by nCRF stimuli (**Fig. S2F-G**) simply because it increased the effective contrast-content of the image (as when aperture was increased, see corresponding section of the main text, and **Fig. 5**). This decrease could be rescued by explaining away via $\mathbf{\Sigma}$ when the nCRF stimulus was appropriately aligned with the projective fields of neurons which had positive noise correlations with the "recorded" neuron (**Fig. S2H**).

6 Establishing relationships between different forms of correlations

In order to establish a relationship for how signal, spontaneous, and noise correlations are related, we demonstrate how learning, inference, and stimulus statistics affect these quantities. First, we establish the link between signal correlations and correlations during spontaneous activity. Next, we derive a relationship between signal correlations and noise correlations. Although the derivations below regard the latent variables of the GSM, $\mathbf{y}$, we also show numerically that these results

carry over to membrane potentials, $\mathbf{u}$ (**Fig. 2** of the main text, and **Fig. S4C**), or firing rates (spike counts), $\mathbf{r}$ (**Fig. 6** of the main text).

Relationship between spontaneous and signal correlations

An important consequence of the adaptation of any well-calibrated internal model to the statistics of input stimuli can be revealed by expressing the prior distribution as the posterior distribution marginalized over the distribution of stimuli (Gelman et al., 2013; Berkes et al., 2011a):

$$\mathrm{P}(\mathbf{y}) = \int \mathrm{P}(\mathbf{y}|\mathbf{x})\ \mathrm{P}(\mathbf{x})\,\mathrm{d}\mathbf{x} \simeq \int \mathrm{P}(\mathbf{y}|\mathbf{x})\ \mathrm{P}^*(\mathbf{x}) \tag{S11}$$

where the average on the right is taken over the ensemble of natural images, $\mathrm{P}^*(\mathbf{x})$, to which the model has previously been adapted (see Section 1). As a result, a similar relationship holds for the first and second moments of responses:

$$\mathrm{E}[y_i] \simeq \mathrm{E}_{\mathbf{x}}[\mathrm{E}[y_i|\mathbf{x}]] \tag{S12}$$

$$\mathrm{E}[y_i\, y_j] \simeq \mathrm{E}_{\mathbf{x}}[\mathrm{E}[y_i\, y_j|\mathbf{x}]] \tag{S13}$$

Using the mapping of spontaneous activity to the prior distribution and stimulus-evoked activity to the posterior distribution (see main text and **Fig. 2**) and the above link between the prior distribution, the average posterior distribution, and the image statistics (Eqs. S12-S13), we can decompose the response covariance during spontaneous activity to a sum of the average noise covariance and the signal covariance:

$$\begin{aligned}
\overbrace{\mathrm{Cov}[y_i, y_j]}^{\text{cov. during spont. act.}} &= \mathrm{E}[y_i\, y_j] - \mathrm{E}[y_i]\ \mathrm{E}[y_j] \\
&= \mathrm{E}_{\mathbf{x}}[\mathrm{E}[y_i\, y_j|\mathbf{x}]] - \mathrm{E}_{\mathbf{x}}[\mathrm{E}[y_i|\mathbf{x}]]\ \mathrm{E}_{\mathbf{x}}[\mathrm{E}[y_j|\mathbf{x}]] \\
&= \mathrm{E}_{\mathbf{x}}[\mathrm{Cov}[y_i, y_j|\mathbf{x}]] + \mathrm{E}_{\mathbf{x}}[\mathrm{E}[y_i|\mathbf{x}]\ \mathrm{E}[y_j|\mathbf{x}]] - \mathrm{E}_{\mathbf{x}}[\mathrm{E}[y_i|\mathbf{x}]]\ \mathrm{E}_{\mathbf{x}}[\mathrm{E}[y_j|\mathbf{x}]] \\
&= \underbrace{\mathrm{E}_{\mathbf{x}}[\mathrm{Cov}[y_i, y_j|\mathbf{x}]]}_{\text{avg. noise cov.}} + \underbrace{\mathrm{Cov}_{\mathbf{x}}[\mathrm{E}[y_i|\mathbf{x}]\,, \mathrm{E}[y_j|\mathbf{x}]]}_{\text{signal cov.}}
\end{aligned} \tag{S14}$$

Inferring basis function activations from a high-contrast image results in a posterior with significantly smaller (co)variances than those in the prior (see **Figs. 2-3**)

and so the second term in Eq. S14 dominates, that is, spontaneous covariances are largely determined by signal covariances (**Fig. 2D**). As a consequence, correlations during spontaneous activity (which can be expressed as a weighted sum of signal and noise correlations, weighted by their respective total variances) will closely resemble signal correlations (**Fig. S4C**):

$$\underbrace{\mathrm{Corr}[y_i, y_j]}_{\text{corr. during spont. act.}} \simeq \underbrace{\mathrm{Corr}_{\mathbf{x}}[\mathrm{E}[y_i|\mathbf{x}]\,, \mathrm{E}[y_i|\mathbf{x}]]}_{\text{signal corr.}} \tag{S15}$$

Although the above argument strictly holds in the case when signal correlations are measured using natural images, it also provides a good fit when grating stimuli are used instead, even (**Fig. S4C**, green vs. blue).

Relationship between signal and noise correlations

Approximating inference over $\mathbf{y}$ being conditioned on the MAP estimate of z, $\bar{z}$ (rather than integrating out the full posterior over z, $\mathrm{P}(z|\mathbf{x})$), as we did in Section 5, we can write the posterior mean of $\mathbf{y}$ (Eq. 5 in Experimental Procedures) as:

$$\mathrm{E}[\mathbf{y}|\mathbf{x}] \simeq \boldsymbol{\mu}(\mathbf{x}, \bar{z}) = \frac{\bar{z}}{\sigma_{\mathrm{x}}^2}\, \boldsymbol{\Sigma}(\bar{z})\ \mathbf{A}^{\mathsf{T}}\, \mathbf{x} \tag{S16}$$

Then, relying on the fact that whitened images are used during training and testing, we assume that $\mathrm{Cov}[\mathbf{x}] \sim \mathbf{I}$. Based on these assumptions, Eq. S14 can be rewritten (again ignoring the contribution of noise covariances as above) as

$$\begin{aligned} \mathrm{Cov}[\mathbf{y}] &\approx \mathrm{E}_{\mathbf{x}}\Big[\boldsymbol{\mu}(\mathbf{x}, \bar{z})\ \boldsymbol{\mu}(\mathbf{x}, \bar{z})^{\mathsf{T}}\Big] \propto \boldsymbol{\Sigma}\,\mathbf{A}^{\mathsf{T}}\,\mathrm{E}\big[\mathbf{x}\,\mathbf{x}^{\mathsf{T}}\big]\ \mathbf{A}\,\boldsymbol{\Sigma} \\ &\propto \boldsymbol{\Sigma}\,\mathbf{A}^{\mathsf{T}}\,\mathbf{A}\,\boldsymbol{\Sigma} \end{aligned} \tag{S17}$$

At high contrast levels, the posterior (noise) covariance (Eq. 5 in Experimental Procedures) can be approximated by

$$\mathrm{Cov}[\mathbf{y}|\mathbf{x}] = \boldsymbol{\Sigma} \propto \left(\mathbf{A}^{\mathsf{T}}\,\mathbf{A}\right)^{-1} \tag{S18}$$

and thus Eq. S17, the expression for prior (spontaneous) correlations, can be simplified as

$$\mathrm{Cov}[\mathbf{y}] \propto \left(\mathbf{A}^{\mathsf{T}}\,\mathbf{A}\right)^{-1} \tag{S19}$$

This establishes the similarity of spontaneous (prior, Eq. S19) and noise (posterior, Eq. S18) correlations. Taken together with the similarity of signal and spontaneous correlations we established in the previous section (Eq. S15), this implies (by transitivity) the similarity of signal and noise correlations that can be tested both in the model and in experiments (**Fig. 6**).

Redundancy and noise correlations in overcomplete models

As we note in the Discussion of the main text, overcomplete representations, in which several latent variables represent similar (or identical) features (such that the corresponding columns of $\mathbf{A}$ are strongly non-orthogonal), may be helpful in increasing the effective number of samples in each time step. This is because in such an overcomplete system, having one sample from each of these redundant variables amounts to having several samples from a variable that represents the single shared feature. The caveat with this argument is that it relies on the assumption that the *posterior distribution* over redundant variables remains sufficiently uncorrelated – as getting many strongly correlated samples would still not be better than getting just a single sample. Yet, intuitively, one would expect explaining away to produce strong (negative) posterior (or noise) correlations between such redundant variables (eg. arising from mutual suppression between neurons representing these variables). However, this intuition really refers to the *likelihood*, so for it to carry over to the *posterior*, it in turn relies on the assumption that the *prior distribution* of redundant variables is sufficiently broad and / or uncorrelated. Crucially, the prior distribution of an overcomplete system is underconstrained by the data, so that different prior distributions will be equally good for modelling the data. (For example, for models in which the likelihood is linear, as in our GSM model, Eqs. 1-2 of the main text, the prior covariance can be anything within the appropriate null space of the feature matrix, $\mathbf{A}$). This leaves a number of degrees of freedom in the prior that could in principle be tuned to minimise the (average) correlation (or statistical dependence) of posteriors. Whether, and to what degree, such optimisation for posterior independence is possible in overcomplete systems will be an important and interesting question for future research.

7 Hierarchical inference and top-down influences on response variability in V1

The model presented in the paper aims to account for the main bottom-up determinants (i.e. signal-driven sources) of the response variability of V1 simple cells. Nevertheless, our approach can also be extended to include higher-order visual cortical areas, and in particular top-down influences on variability as statistical inference and sampling provide a self-consistent way to interpret the activities of neurons, and their interactions, at all levels of the cortical hierarchy.

Here we demonstrate that our main results on bottom-up driven changes in variability hold even in such a hierarchical model and are not simply a special case of the simple, fundamentally linear-Gaussian nature of the GSM model (at a fixed contrast level). We formulate interactions of V1 simple cell activity (or the latent variables represented thereof), $\mathbf{y}$, and higher-level (e.g. V2) neurons, $\mathbf{h}$, such that the prior over $\mathbf{y}$ depends on $\mathbf{h}$, $\mathrm{P}(\mathbf{y}|\mathbf{h})$, in potentially complex ways, rather than being a simple Gaussian. In this case, the posterior over V1-level variables given an input image $\mathbf{x}$ can be written as (c.f. Eq. 5 in Experimental Procedures):

$$\mathrm{P}(\mathbf{y}|\mathbf{x}) = \int \mathrm{P}(\mathbf{y}|\mathbf{h},\mathbf{x})\ \mathrm{P}(\mathbf{h}|\mathbf{x})\,\mathrm{d}\mathbf{h} \tag{S20}$$

Thus, inference of $\mathbf{y}$ upon observing stimulus $\mathbf{x}$ now involves marginalization over higher-level activities. Without loss of generality, we write the expected value of $\mathbf{y}$ given $\mathbf{x}$ and $\mathbf{h}$ as a sum of two terms:

$$\mathrm{E}[\mathbf{y}|\mathbf{h},\mathbf{x}] = \mathrm{E}[\mathbf{y}|\mathbf{h}] + \delta\bar{\mathbf{y}}(\mathbf{h},\mathbf{x}) \tag{S21}$$

where the first term is a stimulus-independent (but $\mathbf{h}$-dependent) component and the second term summarizes the influence of the stimulus. Using the rule of total covariance we can write the response (noise) covariance as:

$$\mathrm{Cov}[\mathbf{y}|\mathbf{x}] = \mathrm{E}_{\mathbf{h}|\mathbf{x}}[\mathrm{Cov}[\mathbf{y}|\mathbf{h},\mathbf{x}]] + \mathrm{Cov}_{\mathbf{h}|\mathbf{x}}[\mathrm{E}[\mathbf{y}|\mathbf{h},\mathbf{x}]] \tag{S22}$$

Given these preliminaries, we now consider the case of a stimulus (e.g. a grating) that does not elicit a concrete higher order percept, such that the posterior over higher-order variables, $\mathbf{h}$, remains similar to the prior:

$$\mathrm{P}(\mathbf{h}|\mathbf{x}) \simeq \mathrm{P}(\mathbf{h}) \tag{S23}$$

This allows us to rewrite the posterior covariance of $\mathbf{y}$ (Eq. S22) as

$$\mathrm{Cov}[\mathbf{y}|\mathbf{x}] \simeq \mathrm{E}_{\mathbf{h}}[\mathrm{Cov}[\mathbf{y}|\mathbf{h},\mathbf{x}]] + \mathrm{Cov}_{\mathbf{h}}[\mathrm{E}[\mathbf{y}|\mathbf{h},\mathbf{x}]] \tag{S24}$$

In general, we expect the sum in Eq. S24 to be dominated by the second term as the amount of variation in (average) V1 activations due to changes in $\mathbf{h}$, $\mathrm{Cov}_{\mathbf{h}}[\mathrm{E}[\mathbf{y}|\mathbf{h},\mathbf{x}]]$, is expected to be much larger than the residual (noise) covariance when both $\mathbf{h}$ and $\mathbf{x}$ are fixed, $\mathrm{E}_{\mathbf{h}}[\mathrm{Cov}[\mathbf{y}|\mathbf{h},\mathbf{x}]]$. This will certainly hold when higher order percepts can elicit reliable responses even in the absence of the actual low-level visual feature that defines the classical receptive field and tuning curve of a cell, as is the case for V1 neurons that respond to illusory contours (Grosof et al., 1993; Lee & Nguyen, 2001) and perceived brightness (Rossi et al., 1996). Thus we can write that

$$\mathrm{Cov}[\mathbf{y}|\mathbf{x}] \mathrel{\tilde{\propto}} \mathrm{Cov}_{\mathbf{h}}[\mathrm{E}[\mathbf{y}|\mathbf{h},\mathbf{x}]] \tag{S25}$$

Next, applying Eq. S21 to S25 yields

$$\begin{aligned}\mathrm{Cov}[\mathbf{y}|\mathbf{x}] &\mathrel{\tilde{\propto}} \mathrm{Cov}_{\mathbf{h}}[\mathrm{E}[\mathbf{y}|\mathbf{h}]] + \mathrm{Cov}_{\mathbf{h}}[\delta\overline{\mathbf{y}}(\mathbf{h},\mathbf{x})] + \\ &\qquad + 2\,\mathrm{Cov}_{\mathbf{h}}[\mathrm{E}[\mathbf{y}|\mathbf{h}]\,,\delta\overline{\mathbf{y}}(\mathbf{h},\mathbf{x})] \qquad \text{(S26)} \\ &\mathrel{\tilde{\propto}} \mathrm{Cov}_{\mathbf{h}}[\mathrm{E}[\mathbf{y}|\mathbf{h}]] \qquad \text{(S27)}\end{aligned}$$

where we made the additional but reasonable assumption that $\mathbf{x}$ elicits consistent changes in (the average) $\mathbf{y}$ across the range of possible settings of $\mathbf{h}$, and so the corresponding covariances (the last two terms in Eq. S26) can be neglected.

We also note that the prior (ie. spontaneous) covariance (of V1-level variables) in this model can be written simply as

$$\mathrm{Cov}[\mathbf{y}] = \mathrm{E}_{\mathbf{h}}[\mathrm{Cov}[\mathbf{y}|\mathbf{h}]] + \mathrm{Cov}_{\mathbf{h}}[\mathrm{E}[\mathbf{y}|\mathbf{h}]] \tag{S28}$$

Again, under the same conditions as above (higher order percepts can elicit reliable responses even in the absence of the appropriate low-level visual feature), we expect this sum to be dominated by the second term, and so we can write

$$\mathrm{Cov}[\mathbf{y}] \mathrel{\tilde{\propto}} \mathrm{Cov}_{\mathbf{h}}[\mathrm{E}[\mathbf{y}|\mathbf{h}]] \tag{S29}$$

Thus, we can conclude from Eq. S27 that when the stimulus carries limited information about higher-level inferences, such as in the case of simplistic artificial

stimuli, the noise correlation structure is largely independent of the stimulus.[†] Comparing Eq. S27 and S29 also shows that, just as in the GSM, in this case prior (spontaneous) and posterior (noise) correlations are also expected to be similar. In addition, note that the arguments for why spontaneous activity distribution (and thus correlations) will match average evoked activity distributions (and thus correlations), and that spontaneous correlations will be dominated by noise correlations (Section 6) are general and do not make use of the specifics of the GSM. This also means that signal and noise correlations will also be similar in this hierarchical model for non-naturalistic stimuli.

For more naturalistic stimuli, the stimulus will afford higher level inferences and constrain the posterior over $\mathbf{h}$ such that its posterior is no longer equivalent to its prior, $\mathrm{P}(\mathbf{h}|\mathbf{x}) \neq \mathrm{P}(\mathbf{h})$ and instead becomes highly constrained. In this case, the two terms in Eq. S24 are averages and covariances over $\mathrm{P}(\mathbf{h}|\mathbf{x})$, not $\mathrm{P}(\mathbf{h})$, and in particular the covariance over $\mathrm{P}(\mathbf{h}|\mathbf{x})$ becomes much smaller. This predicts that noise (co)variability in V1 responses ($\mathbf{y}$) will be reduced for natural stimuli, which agrees with the finding that natural images evoke more reliable and sparser responses than their phase-scrambled counterparts (which do not afford higher-order percepts) (Froudarakis et al., 2014). As a special case, this also implies a hierarchical version of the prediction, tested in the main text (**Figs. 3-5**), that increasing contrast or stimulus aperture (presumably better allowing higher-order percepts to arise) leads to decreased variability and increased reliability, sparseness, and decorrelation, and that these effects should be particularly prominent when using natural stimuli (Haider et al., 2010; Vinje & Gallant, 2000).

Table S2 summarises these results on how hierarchical inference accounts for all the main effects obtained by using a GSM elsewhere in the paper.

[†]As a sanity check, note that using the same assumptions, the response mean is – quite appropriately – *not* independent of the stimulus:

$$\begin{aligned}\mathrm{E}[\mathbf{y}|\mathbf{x}] &= \mathrm{E}_{\mathbf{h}|\mathbf{x}}[\mathrm{E}[\mathbf{y}|\mathbf{h},\mathbf{x}]] \simeq \mathrm{E}_{\mathbf{h}}[\mathrm{E}[\mathbf{y}|\mathbf{h},\mathbf{x}]] \\ &= \mathrm{E}_{\mathbf{h}}[\mathrm{E}[\mathbf{y}|\mathbf{h}]] + \mathrm{E}_{\mathbf{h}}[\delta\overline{\mathbf{y}}(\mathbf{h},\mathbf{x})] = \mathrm{E}[\mathbf{y}] + \mathrm{E}_{\mathbf{h}}[\delta\overline{\mathbf{y}}(\mathbf{h},\mathbf{x})]\end{aligned} \tag{S30}$$

and there is no reason to assume that the expectation (rather than the variance) of $\delta\overline{\mathbf{y}}(\mathbf{h},\mathbf{x})$ is small (relative to the prior mean of $\mathbf{y}$, $\mathrm{E}[\mathbf{y}]$).

Nonlinear relationship between low-level and high-level confidence

While our model only predicted (in line with experimental data) a relatively modest (less than a factor of 2) reduction in (membrane potential) variance at the level of V1 at the onset of a high contrast stimulus (**Fig. 3**), perceptual confidence typically undergoes a drastic, orders-of-magnitude change under the same condition. This seeming contradiction can be reconciled by considering nonlinear effects that are ubiquitous in inference under hierarchical models (in which perceptual confidence corresponds to inferences over higher-level variables). The key insight is that the variance of the posterior over a higher-level variable will in general depend not only on the variance of a low level posterior but also on its mean.

As a toy example, consider a generative model[‡] with a single, binary higher level variable $h = \pm 1$ (related to eg. whether one or another stimulus is present in a two-alternative forced choice perceptual decision making task) which is related to V1-level variables through a simple (multivariate) normal likelihood, such as:

$$\mathrm{P}(h) = \mathrm{Bernoulli}\left(\frac{h+1}{2}; \frac{1}{2}\right) \tag{S31}$$

$$\mathrm{P}(\mathbf{y}|h) = \mathcal{N}\left(\mathbf{y}; \frac{\mathbf{a}\,h}{2}, \sigma_{\mathrm{y}}^2\,\mathbf{I}\right) \tag{S32}$$

In this model, the conditional probability of $h = +1$ for a given $\mathbf{y}$ can be written as

$$\mathrm{P}(h = +1|\mathbf{y}) = \mathrm{Sigmoid}(f)\,, \qquad \text{with } f = \frac{\mathbf{a}^{\mathsf{T}}\,\mathbf{y}}{\sigma_{\mathrm{y}}^2} \tag{S33}$$

where $\mathrm{Sigmoid}(f) = 1/\left(1 + e^{-f}\right)$ is the standard logistic sigmoid function (**Fig. S3E**, middle, red curve). Finally, the posterior over h given an input image $\mathbf{x}$ can be written by marginalising out the posterior over $\mathbf{y}$:

$$\mathrm{P}(h = +1|\mathbf{x}) = \int \mathrm{P}(h = +1|\mathbf{y})\;\mathrm{P}(\mathbf{y}|\mathbf{x})\,\mathrm{d}\mathbf{y} \tag{S34}$$

Note that this marginalisation really only depends on the relevant projection of $\mathbf{y}$, that is f, and the projected posterior will usually be approximately normal

[‡]This model is a simplified special case of the model we consider for decoding a multinomial orientation variable from V1 responses in Section 8.

(due to the central limit theorem, if $\mathbf{y}$ is sufficiently high dimensional – and for the GSM also because the individual elements of $\mathbf{y}$ are already approximately normally distributed even under the posterior – **Fig. S3E**, middle, blue and brown Gaussians). Thus, Eq. S34 can be rewritten as

$$\mathrm{P}(h=+1|\mathbf{x}) \simeq \int \mathrm{Sigmoid}(f)\ \mathcal{N}\big(f;\mu_\mathrm{f},\sigma_\mathrm{f}^2\big)\,\mathrm{d}f \tag{S35}$$

$$\text{where } \mu_\mathrm{f} = \mathbf{a}^\mathsf{T}\,\boldsymbol{\mu}_\mathrm{y}/\sigma_\mathrm{y}^2, \text{ and } \sigma_\mathrm{f}^2 = \mathbf{a}^\mathsf{T}\,\boldsymbol{\Sigma}_\mathrm{y}\,\mathbf{a}/\sigma_\mathrm{y}^4 \tag{S36}$$

and $\boldsymbol{\mu}_\mathrm{y}$ and $\boldsymbol{\Sigma}_\mathrm{y}$ are the posterior mean and covariance of $\mathbf{y}$. In general (as in our non-hierarchical GSM model), there are at least two aspects of the $\mathbf{y}$-posterior (and its f-projection) that change when contrast goes to zero: its (co)variance grows, and its mean goes towards zero (see main text and **Fig. 2**). Crucially, the h-posterior, $\mathrm{P}(h=+1|\mathbf{x})$, and thus its variance depends on *both* these statistics, not only the change in the variance of $\mathbf{y}$ (or f). Our goal is to understand how changes in the variance of low-level variables, α_y^2, relate to changes in the variance of the high-level variable, α_h^2, in this model.

For an illustration, we consider two cases, before stimulus onset, and after the onset of a stimulus which is such that the posterior mean of $\mathbf{y}$ is just as expected for the $+1$ stimulus, i.e. $\boldsymbol{\mu}_\mathrm{y} = \mathbf{a}$ (**Fig. S3E**, bottom, blue vs. brown distributions). Thus, the f-posterior will have a mean $\mu_\mathrm{f} = 0$ and $\mu_\mathrm{f} = \mu_\mathrm{f}^+ = \mathbf{a}^\mathsf{T}\,\mathbf{a}/\sigma_\mathrm{y}^2$ and variance $\sigma_\mathrm{f}^2 = \sigma_\mathrm{f0}^2$ and $\sigma_\mathrm{f}^2 = \sigma_\mathrm{f0}^2/\alpha_\mathrm{y}^2$, before and after stimulus onset, respectively, where $\alpha_\mathrm{y}^2 > 1$ is the relative reduction of $\mathbf{y}$-variance at stimulus onset (**Fig. S3E**, middle, blue vs. brown Gaussians). Note that as f is a linear projection of $\mathbf{y}$, the variance of its (normally distributed) posterior will also be reduced by the same factor, α_y^2. As before stimulus onset, the mean of f is sitting at exactly $\mu_\mathrm{f} = 0$, which is the classification boundary for h in f-space (**Fig. S3E**, straight red line), and its distribution is symmetric around it (being approximately normal), its probability mass on the two sides of the boundary will be equal, and so the posterior distribution of h is uniform. Thus, before stimulus onset, the posterior variance of h is maximal, i.e. it is equal to 1 (**Fig. S3E**, top, blue). After stimulus onset, the h-posterior is a Bernoulli variable with probability (**Fig. S3E**, top, brown):

$$\mathrm{P}(h=+1|\mathbf{y}) = \int \mathrm{Sigmoid}(f)\ \mathcal{N}\big(f;\mu_\mathrm{f}^+,\sigma_\mathrm{f0}^2/\alpha_\mathrm{y}^2\big)\,\mathrm{d}f \tag{S37}$$

from which the relative reduction in the variance of h can be obtained as

$$\alpha_\mathrm{h}^2 = \frac{1}{4\,\mathrm{P}(h=+1|\mathbf{y})\ [1-\mathrm{P}(h=+1|\mathbf{y})]} \tag{S38}$$

In sum, Eqs. S37-S38 establish the dependence of α_{h}^2 on α_{y}^2 and identify the relevant parameters of this dependence as μ_{f}^{+} ('signal') and σ_{f0}^2 ('noise', **Fig. S3F**). As we can see, there is a nonlinear interplay between the effect of the mean and variance of the $\mathbf{y}$-posterior on inferences about h, such that especially for high signal and low noise, the reduction in h-variance, α_{h}^2, can be orders of magnitude greater than the reduction in $\mathbf{y}$-variance, α_{y}^2. In particular, note that even when there is no reduction in $\mathbf{y}$-variance ($\alpha_{\mathrm{y}}^2 = 1$), there can still be substantial reduction in h-variance ($\alpha_{\mathrm{h}}^2 \gg 1$).

Finally, we expect (as long as the $\mathbf{y}$ posterior only has finite-range correlations, as in our GSM model) both μ_{f}^{+} and σ_{f0}^2 to scale linearly with N (the number of V1-level variables on which the perceptual decision depends). Thus, in the limit of large N, the posterior over f goes to a delta distribution which simplifies Eq. S37 to

$$\mathrm{P}(h = +1|\mathbf{x}) \simeq \mathrm{Sigmoid}\big(\mu_{\mathrm{f}}^{+}\big) \tag{S39}$$

and so plugging Eq. S39 into Eq. S38 reveals that $\alpha_{\mathrm{h}}^2 \propto e^N$. In other words, the reduction in h-variance can be arbitrarily large (with sufficiently high N) irrespective of the value of α_{y}^2.

8 The relevance of stimulus-dependent changes in neural variability: decoding stimuli from spike trains

Our sampling-based model encodes the posterior distribution over visual features such that Fano factors and noise correlations of V1 responses depend on particular attributes of the stimulus (Figs. 4-6). This is in contrast with other probabilistic encoding schemes that do not predict such stimulus-dependent modulations, and in fact specifically assume their absence (Ma et al., 2006). Although experimental data do show the modulations that our theory predicts (Churchland et al., 2010; Ecker et al., 2010; Finn et al., 2007; Haider et al., 2010; Carandini, 2004; Berkes et al., 2011a) (**Figs. 4-6**), it is difficult to assert whether these are actually relevant for neural computations or they can be regarded epiphenomenal. One prominent approach to make inroads in this issue employs a decoding framework: it is based on measuring how well the stimulus can be inferred from (simulated or experimentally measured) neural responses using different decoder algorithms (Averbeck et al., 2006; Pillow et al., 2008). The relevance of a particular aspect of

the neural code, such as the stimulus-dependence of (co)variability in our model, can then be assessed by comparing a decoder which takes that aspect into account with one that ignores it: if the two decoders perform similarly then the given aspect of the neural code can be deemed irrelevant, if they do not then it is likely functionally relevant.

To assess the functional relevance of stimulus-dependent changes in variability in our model, we compared the performance of two algorithms decoding the stimulus from spike counts generated by the model (**Fig. S5**). The first was a linear decoder, which ignored these variability modulations (Berens et al., 2012; Ma, 2010) but was otherwise optimised for inferring the stimulus. The second was an optimal decoder, which took the variability modulations into account in a formally optimal way by explicitly inverting the probabilistic process that led from stimulus to spike counts in the model (**Fig. 1B**, Experimental Procedures, see also the end of this section for the mathematical definition of the two decoders). Spike counts were measured in 20 ms windows from 42 randomly chosen neurons in response to static gratings that varied in four attributes: orientation (12 different values), phase (12), contrast levels (7), and aperture size (7). In a given decoding task, two of these attributes remained fixed and known to the decoders, while the other two were unknown and varied from trial to trial. Of the two unknown attributes, one needed to be decoded while the other disregarded (which thus acted as a nuisance parameter that needed to be marginalised out). Performance of the decoders was assessed by the fraction of trials on which they assigned the highest probability to the correct value of the stimulus attribute that needed to be decoded.

When orientation had to be decoded at unknown contrasts, the linear and optimal decoders performed similarly (**Fig. S5A**). However, when orientation had to be decoded at known contrast levels but at unknown phases, or when stimulus phase had to be decoded with orientation unknown, the optimal decoder substantially outperformed the linear decoder at high contrast levels (**Fig. S5B-C**). There was a similar difference between the two decoders at high contrast levels for orientation decoding with unknown aperture sizes (**Fig. S5D**). Training individual linear decoders for distinct (discretized) values of the nuisance parameters abolished the advantage of the optimal decoder (data not shown), indicating that knowledge of nuisance parameters would ameliorate their effect on a linear decoder.

These results indicate that our sampling-based population code cannot be generally decoded, and thus interpreted, as a linear invariant PPC (Ma et al., 2006).

While it may still be possible to decode our network as a more general linear (non-invariant) PPC, there is currently no theory for how such a PPC could be instantiated in cortical circuits[§]. In sum, the variability modulations in our model that we tested against experimental data (**Figs. 4-6**) are functionally relevant in a variety of scenarios, making sampling-based representations fundamentally distinct from other probabilistic encoding schemes, such as probabilistic population codes for which linear decoding is optimal (Ma et al., 2006).

Although previous decoding-based analyses of experimental data suggested that linear decoders might be sufficient to achieve optimal decoding performance (Graf et al., 2011; Berens et al., 2012), they only considered simple decoding tasks in which one particular attribute, orientation, had to be decoded either without any nuisance parameters, or only contrast as a nuisance parameter – in which case we also found linear decoding to be near-optimal (**Fig. S5A**). (Note that using shifting rather than static gratings of stimuli, as was done in Graf et al., 2011, is not equivalent to requiring the decoder to marginalise over phase.) However, our results suggest that a sound experimental test of the importance of response variability modulations should include more diverse and challenging decoding tasks employing a richer set of possible combinations for which stimulus attributes need to be decoded and which others marginalised. We predict that once such tasks are considered, linear decoding of population responses will significantly fall short of being optimal.

Definition of decoders

The linear decoder assessed the probability of a stimulus attribute, θ, given a vector of spike counts in the population, $\mathbf{n}$, as the following:

$$\mathrm{P}(\theta|\mathbf{n}) = \frac{\exp\left(\mathbf{w}_\theta^\mathsf{T}\mathbf{n} + \eta_\theta\right)}{\exp\left(\sum_{\theta'} \mathbf{w}_{\theta'}^\mathsf{T}\mathbf{n} + \eta_{\theta'}\right)} \tag{S40}$$

where $\mathbf{w}_\theta$ and η_θ are a vector of decoder weights and a scalar bias associated to the particular value of the stimulus attribute, and the denominator provides a normalization of the probability. The decoder was trained (its parameters, $\mathbf{w}_\theta$ and η_θ, optimized) on a set of stimulus-response pairs corresponding to the specific

[§]Although it might be possible to extend the approach of Beck et al. (2011) to this particular problem.

decoding task. The training data consisted of 2400 repetitions of each value of the decoded stimulus attribute while the nuisance parameter was varied randomly and uniformly and the other parameters were kept fixed. (Note that this included multiple presentations of each stimulus which was necessary because model responses were stochastic.) Testing was performed in cross validation, i.e. using a data set of responses that were separate from that used for training.

The optimal decoder used the generative model to compute the probability of a particular stimulus attribute given the spike counts of model neurons. The generative model defined the posterior distribution over visual feature activations, $\mathbf{y}$ given the stimulus $\mathbf{x}$ (Eqs. 2-5 in Experimental Procedures), and feature activations were encoded deterministically in membrane potentials and, in turn, firing rates $\mathbf{r}$ (Eqs. 6-7 in Experimental Procedures). As a decoding window of $T = 20\,\mathrm{ms}$ was used in our simulations, the spike counts recorded, $\mathbf{n}$, represented a single sample from the posterior, and thus the conditional probability of observing spike counts $\mathbf{n}$ could be formalised using a multivariate Bernoulli distribution (over $\lfloor r_i T \rfloor$ and $\lfloor r_i T \rfloor + 1$):

$$\mathrm{P}(\mathbf{n}|\mathbf{r}) = \prod_i \mathrm{P}(n_i|r_i) \tag{S41}$$

$$\mathrm{P}(n_i|r_i) = \begin{cases} \mathrm{Bernoulli}(n_i - \lfloor r_i T \rfloor; r_i - \lfloor r_i T \rfloor) & n_i - \lfloor r_i T \rfloor \in \{0, 1\} \\ 0 & \text{otherwise} \end{cases} \tag{S42}$$

As only $\mathbf{x}$ and $\mathbf{n}$ but not $\mathbf{y}$ (or z) were directly known to the decoder, $\mathbf{y}$ (and z) needed to be marginalized out to obtain the likelihood of a particular stimulus:

$$\mathrm{P}(\mathbf{n}|\mathbf{x}) = \int \mathrm{P}(\mathbf{n}|\mathbf{r}(\mathbf{y}))\ \mathrm{P}(\mathbf{y}|\mathbf{x}, z)\ \mathrm{P}(z|\mathbf{x})\ d\mathbf{y}\, dz \tag{S43}$$

Computing the multi-dimensional integral in Eq. S43 is intractable, therefore we performed a Monte Carlo integral to approximate the true value by obtaining samples from $\mathrm{P}(z|\mathbf{x})$ and $\mathrm{P}(\mathbf{y}|\mathbf{x}, z)$. Finally, the particular stimulus attribute of interest can be decoded using the (uniform) prior over stimulus attributes, $\mathrm{P}(\theta)$, and the distribution $\mathrm{P}(\mathbf{x}|\theta)$, which is uniform over the discrete and finite set of stimuli which are obtained by keeping this attribute fixed while varying the nuisance attribute of the stimulus:

$$\mathrm{P}(\theta|\mathbf{n}) \propto \mathrm{P}(\theta) \sum_{\mathbf{x}} \mathrm{P}(\mathbf{n}|\mathbf{x})\ \mathrm{P}(\mathbf{x}|\theta) \tag{S44}$$

As only a small number of stimuli were compatible with any given value of θ, the sum in Eq. S44 was evaluated by simple enumeration. Note that the optimal decoder did not have free parameters and thus it did not need a separate training phase.

References

Anderson, J. S., Lampl, I., Gillespie, D. C., & Ferster, D. (2000). The contribution of noise to contrast invariance of orientation tuning in cat visual cortex. *Science*, *290*(5498), 1968–1972.

Averbeck, B. B., Latham, P. E., & Pouget, A. (2006). Neural correlations, population coding and computation. *Nature Reviews Neuroscience*, *7*(5), 358–366.

Azouz, R., & Gray, C. M. (1999). Cellular mechanisms contributing to response variability of cortical neurons in vivo. *Journal of Neuroscience*, *19*(6), 2209–2223.

Beck, J. M., Latham, P. E., & Pouget, A. (2011). Marginalization in Neural Circuits with Divisive Normalization. *Journal of Neuroscience*, *31*, 15310–15319.

Berens, P., Ecker, A. S., Cotton, R. J., Ma, W. J., Bethge, M., & Tolias, A. S. (2012). A fast and simple population code for orientation in primate V1. *Journal of Neuroscience*, *32*(31), 10618–10626.

Berkes, P., Orbán, G., Lengyel, M., & Fiser, J. (2011a). Spontaneous cortical activity reveals hallmarks of an optimal internal model of the environment. *Science*, *331*(6013), 83–87.

Berkes, P., Turner, R. E., & Fiser, J. (2011b). The army of one (sample): the characteristics of sampling-based probabilistic neural representations. In *Frontiers in Neuroscience*.

Büsing, L., Bill, J., Nessler, B., & Maass, W. (2011). Neural dynamics as sampling: a model for stochastic computation in recurrent networks of spiking neurons. *PLoS Computational Biology*, *7*(11), e1002211.

Carandini, M. (2004). Amplification of trial-to-trial response variability by neurons in visual cortex. *PLoS Biology*, *2*(9), E264.

Churchland, M. M., Yu, B. M., Cunningham, J. P., Sugrue, L. P., Cohen, M. R., Corrado, G. S., Newsome, W. T., Clark, A. M., Hosseini, P., Scott, B. B., Bradley, D. C., Smith, M. A., Kohn, A., Movshon, J. A., Armstrong, K. M., Moore, T., Chang, S. W., Snyder, L. H., Lisberger, S. G., Priebe, N. J., Finn, I. M., Ferster, D., Ryu, S. I., Santhanam, G., Sahani, M., & Shenoy, K. V. (2010). Stimulus onset quenches neural variability: a widespread cortical phenomenon. *Nature Neuroscience*, *13*(3), 369–378.

de La Rocha, J., Doiron, B., Shea-Brown, E., Josić, K., & Reyes, A. (2007). Correlation between neural spike trains increases with firing rate. *Nature*, *448*(7155), 802–806.

Dempster, A. P., Laird, N. M., & Rubin, D. B. (1977). Maximum likelihood from incomplete data via the EM algorithm. *Journal of the Royal Statistical Society. Series B (Methodological)*, *39*, 1–38.

DeWeese, M. R., & Zador, A. M. (2006). Non-Gaussian membrane potential dynamics imply sparse, synchronous activity in auditory cortex. *Journal of Neuroscience*, *26*(47), 12206–12218.

Ecker, A. S., Berens, P., Cotton, R. J., Subramaniyan, M., Denfield, G. H., Cadwell, C. R., Smirnakis, S. M., Bethge, M., & Tolias, A. S. (2014). State dependence of noise correlations in macaque primary visual cortex. *Neuron*, *82*(1), 235–248.

Ecker, A. S., Berens, P., Keliris, G. A., Bethge, M., Logothetis, N. K., & Tolias, A. S. (2010). Decorrelated neuronal firing in cortical microcircuits. *Science*, *327*(5965), 584–587.

Finn, I. M., Priebe, N. J., & Ferster, D. (2007). The emergence of contrast-invariant orientation tuning in simple cells of cat visual cortex. *Neuron*, *54*(1), 137–152.

Froudarakis, E., Berens, P., Ecker, A. S., Cotton, R. J., Sinz, F. H., Yatsenko, D., Saggau, P., Bethge, M., & Tolias, A. S. (2014). Population code in mouse V1 facilitates readout of natural scenes through increased sparseness. *Nature Neuroscience*, *17*(6), 851–857.

Gelman, A., Carlin, J. B., Stern, H. S., Dunson, D. B., Vehtari, A., & Rubin, D. B. (2013). *Bayesian Data Analysis*. CRC Press, 3rd ed.

Goris, R. L. T., Movshon, J. A., & Simoncelli, E. P. (2014). Partitioning neuronal variability. *Nature Neuroscience*, *17*(6), 858–865.

Grabska-Barwinska, A., Beck, J., Pouget, A., & Latham, P. (2013). Demixing odors - fast inference in olfaction. In C. J. C. Burges, L. Bottou, M. Welling, Z. Ghahramani, & K. Q. Weinberger (Eds.) *Advances in Neural Information Processing Systems 26*, (pp. 1968–1976). Curran Associates, Inc.

Graf, A. B. A., Kohn, A., Jazayeri, M., & Movshon, J. A. (2011). Decoding the activity of neuronal populations in macaque primary visual cortex. *Nature Neuroscience*, *14*(2), 239–245.

Grosof, D. H., Shapley, R. M., & Hawken, M. J. (1993). Macaque V1 neurons can signal 'illusory' contours. *Nature*, *365*(6446), 550–552.

Haider, B., Häusser, M., & Carandini, M. (2013). Inhibition dominates sensory responses in the awake cortex. *Nature*, *493*(7430).

Haider, B., Krause, M. R., Duque, A., Yu, Y., Touryan, J., Mazer, J. A., & McCormick, D. A. (2010). Synaptic and network mechanisms of sparse and reliable visual cortical activity during nonclassical receptive field stimulation. *Neuron*, *65*(1), 107–121.

Hennequin, G., Aitchison, L., & Lengyel, M. (2014). Fast Sampling-Based Inference in Balanced Neuronal Networks. In Z. Ghahramani, M. Welling, C. Cortes, N. D. Lawrence, & K. Q. Weinberger (Eds.) *Advances in Neural Information Processing Systems 27*, (pp. 2240–2248). Curran Associates, Inc.

Karklin, Y., & Lewicki, M. S. (2009). Emergence of complex cell properties by learning to generalize in natural scenes. *Nature*, *457*(7225), 83–86.

Kohn, A., & Smith, M. A. (2005). Stimulus dependence of neuronal correlation in primary visual cortex of the macaque. *Journal of Neuroscience*, *25*(14), 3661–3673.

Lee, T. S., & Nguyen, M. (2001). Dynamics of subjective contour formation in the early visual cortex. *Proceedings of the National Academy of Sciences of the United States of America*, *98*(4), 1907–1911.

Ma, W. J. (2010). Signal detection theory, uncertainty, and Poisson-like population codes. *Vision Research*, *50*(22), 2308–2319.

Ma, W. J., Beck, J. M., Latham, P. E., & Pouget, A. (2006). Bayesian inference with probabilistic population codes. *Nature Neuroscience*, *9*(11), 1432–1438.

Mante, V., Frazor, R. A., Bonin, V., Geisler, W. S., & Carandini, M. (2005). Independence of luminance and contrast in natural scenes and in the early visual system. *Nature Neuroscience*, *8*(12), 1690–1697.

Moreno-Bote, R., Beck, J., Kanitscheider, I., Pitkow, X., Latham, P., & Pouget, A. (2014). Information-limiting correlations. *Nature Neuroscience*, *17*(10), 1410–1417.

Okun, M., Mokeichev, A., Katz, Y., & Lampl, I. (2009). On the dynamics of synaptic inputs during ongoing activity in the cortex. In K. Josić, M. A. Matias, J. Rubin, & R. Romo (Eds.) *Coherent Behavior in Neuronal Networks*, (pp. 1–16). Springer.

Olshausen, B. A. (1996). Learning linear, sparse, factorial codes. Tech. Rep. AIM-1580, Artificial Intelligence Laboratory, Massachusetts Institute of Technology.

Olshausen, B. A., & Field, D. J. (1996). Emergence of simple-cell receptive field properties by learning a sparse code for natural images. *Nature*, *381*(6583), 607–609.

Pillow, J. W., Shlens, J., Paninski, L., Sher, A., Litke, A. M., Chichilnisky, E. J., & Simoncelli, E. P. (2008). Spatio-temporal correlations and visual signalling in a complete neuronal population. *Nature*, *454*(7207), 995–999.

Portilla, J., & Simoncelli, E. P. (2000). A parametric texture model based on joint statistics of complex wavelet coefficients. *International Journal of Computer Vision*, *40*(1), 49–70.

Pouget, A., Beck, J. M., Ma, W. J., & Latham, P. E. (2013). Probabilistic brains: knowns and unknowns. *Nature Neuroscience*, *16*(9), 1170–1178.

Priebe, N. J., Mechler, F., Carandini, M., & Ferster, D. (2004). The contribution of spike threshold to the dichotomy of cortical simple and complex cells. *Nature Neuroscience*, *7*(10), 1113–1122.

Rao, R. P. N., & Ballard, D. H. (1999). Predictive coding in the visual cortex: a functional interpretation of some extra-classical receptive-field effects. *Nature Neuroscience*, *2*(1), 79–87.

Rossi, A. F., Rittenhouse, C. D., & Paradiso, M. A. (1996). The representation of brightness in primary visual cortex. *Science*, *273*(5278), 1104–1107.

Savin, C., Dayan, P., & Lengyel, M. (2014). Optimal recall from bounded metaplastic synapses: predicting functional adaptations in hippocampal area CA3. *PLoS Computational Biology*, *10*(2), e1003489.

Schwartz, O., Sejnowski, T. J., & Dayan, P. (2009). Perceptual organization in the tilt illusion. *Journal of Vision*, *9*(4), 19.1–20.

Schwartz, O., & Simoncelli, E. P. (2001). Natural signal statistics and sensory gain control. *Nature Neuroscience*, *4*(8), 819–825.

van Hateren, J. H. (1992). Real and optimal neural images in early vision. *Nature*, *360*(6399), 68–70.

Vinje, W. E., & Gallant, J. L. (2000). Sparse coding and decorrelation in primary visual cortex during natural vision. *Science*, *287*(5456), 1273–1276.

Wainwright, M. J., & Simoncelli, E. P. (2000). Scale mixtures of Gaussians and the statistics of natural images. In S. A. Solla, T. K. Leen, & K.-R. Muller (Eds.) *Advances in Neural Information Processing Systems 12*, (pp. 855–861). MIT Press, Cambridge, MA.

figure	reference	species	condition	recording
3A-C	Finn et al. (2007) (as analysed in Churchland et al., 2010)	cat	anesthesia	intracellular single cell
3D	Kohn & Smith (2005) (as analysed in Churchland et al., 2010)	macaque	anesthesia	extracellular multi-electrode single unit
4A,C	Finn et al. (2007)	cat	anesthesia	intracellular single cell
3E, 4B,D,E, 6	Ecker et al. (2010)	macaque	awake	extracellular multi-electrode single unit
5A,B,C	Haider et al. (2010)	cat	anesthesia	intracellular single cell
5D	Vinje & Gallant (2000)	macaque	awake	extracellular multi-electrode multiunit
7	Berkes et al. (2011a)	ferret	awake	extracellular multi-electrode multiunit

Table S1. Related to Figs. 3–7. Summary of experimental data to which model results and predictions were compared. See data selection criteria in Section 2.

**result**	**GSM**	**hierarchical model**
stimulus onset quenches variability	✓(Fig. 3)	✓
contrast decreases variability	✓(Fig. 4)	✓
aperture increases reliability, sparseness, and decorrelation	✓(Fig. 5)	✓ esp. for natural stimuli
natural stimuli increases reliability, sparseness, and decorrelation	×	✓
noise correlations do not depend on the stimulus	✓(Fig. 2)	for non-natural stimuli
spontaneous correlations are similar to signal correlations	✓(Fig. 6)	✓
noise correlations are similar to signal correlations	✓(Fig. 6)	for non-natural stimuli
spontaneous and evoked activity distributions are similar	✓(Fig. 7)	✓

Table S2. Related to Figs. 3–7. Generalization of the results with a GSM to a hierarchical inference model (see details in Section 7). Figure numbers in parentheses refer to the main text.

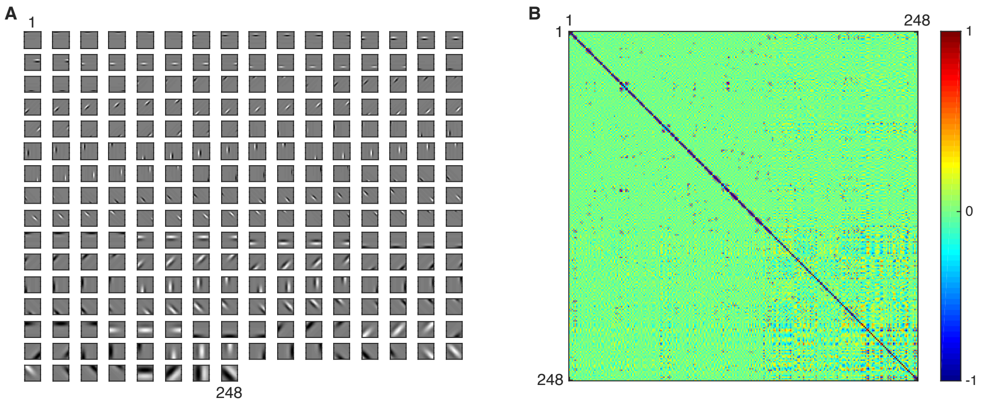

Figure S1. Related to Fig. 1. Model parameters. (**A**) Basis functions (columns of **A**) in the model. Each panel shows one of the 248 columns of **A** rearranged as a 16×16 image, revealing oriented, band-pass, localized filters that were synthetized using one of four orientations and four spatial frequencies. (**B**) Correlations of the prior covariance matrix of the model, obtained by training the model on natural images.

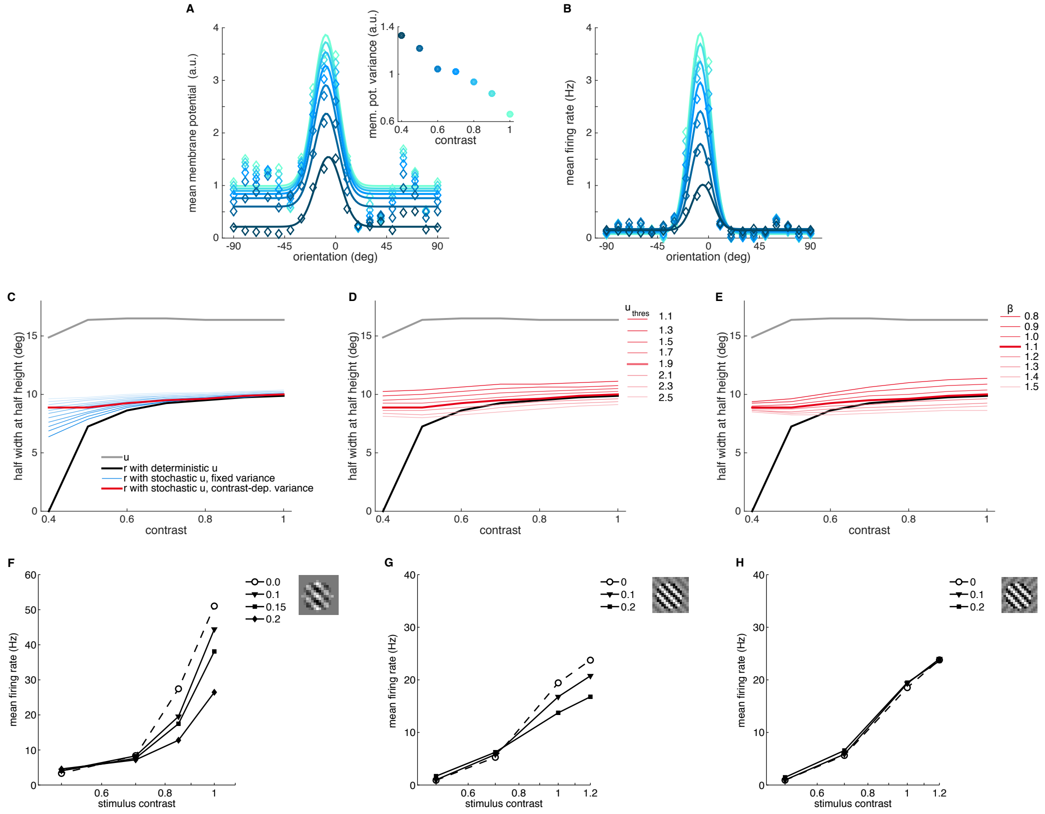

Figure S2 ***(continued on following page)*.**

Figure S2 ***(preceding page).*** **Related to Fig. 2.** Contrast-invariance and non-classical receptive field effects in a representative model neuron. (**A**-**B**) Contrast-dependence of membrane potential (**A**) and firing rate tuning curves (**B**) for orientation (diamonds). Different colors represent different contrast levels (for reference, see inset). Solid lines show circular Gaussian fits with baseline offset, amplitude, center, and width parameters fitted individually for each contrast level. Inset: membrane potential variance at different contrast levels. Colors as in the main main panel. (**C**) Half-width at half-height of different (actual and hypothetical) tuning curves as a function of contrast. Gray line: actual membrane potential tuning curves (from panel **A**); black line: hypothetical firing rate tuning curves assuming no noise variance in membrane potentials around membrane potential tuning curves; blue lines: hypothetical firing rate tuning curves assuming a fixed amount of membrane potential noise variance across different levels of contrast (from dark to light blue: fixed variance increases from that of the highest contrast posterior to the prior variance of the neuron); red line: actual firing rate tuning curves (from panel **B**) based on membrane potential noise variance that decreases with contrast (as shown in panel **A**, inset). (**D, E**) As in **C**, but showing the effects of varying the parameters of the firing rate nonlinearity (**D**: threshold, u_{thresh}; **E**: power-law exponent, β; see also Eq. 7 in Experimental Procedures). Thick red lines correspond to parameter values used in all other figures. (**F**) Cross-orientation suppression of mean firing rate responses. A grating stimulus (inset) with a range of contrast levels (x-axis) was presented at the neuron's preferred orientation while an orthogonally oriented grating stimulus was presented at a different contrast level (symbols). The orthogonal stimulus gradually suppressed (solid lines and filled symbols) the response given to the optimal stimulus alone (dashed line, open symbols). (**G, H**) Selective suppression of the mean firing rate response by non-classical receptive field stimulation. A grating stimulus (inset) with a range of contrast levels (x-axis) was presented at the neuron's preferred orientation in the classical receptive field while a parallel (**G**) or orthogonal grating stimulus (**H**) was presented in the surround non-classical receptive field at different contrast levels (symbols). The parallel surround stimulus gradually suppressed (solid lines and filled symbols, **G**) the response given to the center stimulus alone (dashed line, open symbols), while an orthogonally oriented surround stimulus left it unchanged (**H**).

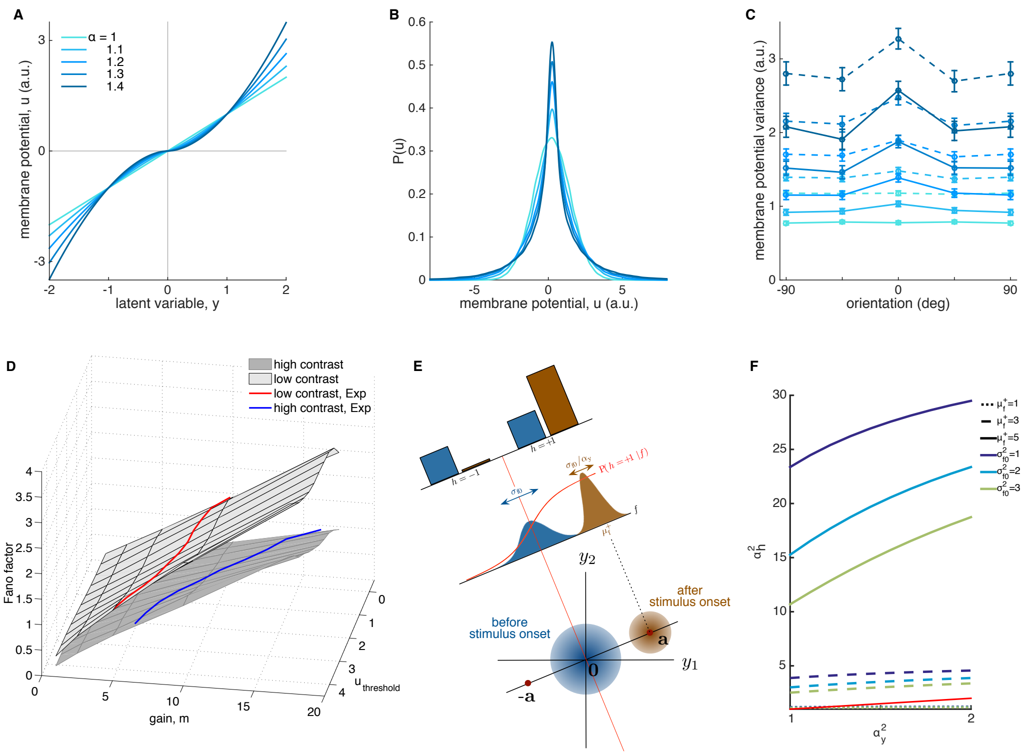

Figure S3 ***(continued on following page)*****.**

Figure S3 ***(preceding page).*** **Related to Figs. 3-4.** Response variability in the model: parameter dependence and implications for perceptual confidence. (**A-C**) Effect of membrane potential nonlinearity on response statistics. (**A**) Mapping from y to u for different values of the exponent of the nonlinearity, α (see also Eq. 6 of the main text). (**B**) Distribution of membrane potentials, u, at different values of α (colors as in **A**). Note sharper peak and larger tails for higher values of α. (**C**) Dependence of membrane potential variance on orientation as a function of α (colors as in **A**). Continuous lines represent the variance of high-contrast responses, dashed lines show the variance of low-contrast responses (cf. **Fig. 4C** of the main text). (**D**) Effect of firing rate nonlinearity on (mean matched) Fano factors. Two free parameters of the firing rate nonlinearity: threshold (u_{thresh}) and gain (m, Eq. 7 of the main text) are explored for spontaneous activity (light gray) and activity evoked by high contrast grating stimuli (dark gray). Red and blue lines show experimentally observed Fano factors (Churchland et al., 2010). (**E**) Schematic of inference under a hierarchical model. y_1, y_2: low (V1-)level variables, f: their summary statistic relevant for a perceptual decision, h: high-level (binary) perceptual decision variable. Blue and brown distributions respectively show posteriors before and after stimulus onset, red sigmoid curve and straight line show classification function and boundary relating $\mathbf{y}$ and f to h. Note that the large shift in h towards $+1$ at stimulus onset, and the consequent drastic reduction in its variance, is mainly driven by a shift of the *mean* of $\mathbf{y}$ away from the classification boundary, rather than the relatively modest reduction in the *variance* of $\mathbf{y}$. See text (Section 7) for further details. (**F**) Relationship between the reduction in low-level variance, α_{y}^2 (as for our simulated membrane potential variances), and the reduction in high-level variance, α_{h}^2 (as reflected in perceptual experience), at stimulus onset in a simple hierarchical model of perceptual decision making as parametrised by different levels of 'signal', μ_{f}^{+} (dotted, dashed, and solid lines), and 'noise', σ_{f0}^2 (dark blue, light blue, green). The red line shows the identity transformation for reference. See text (Section 7) for details.

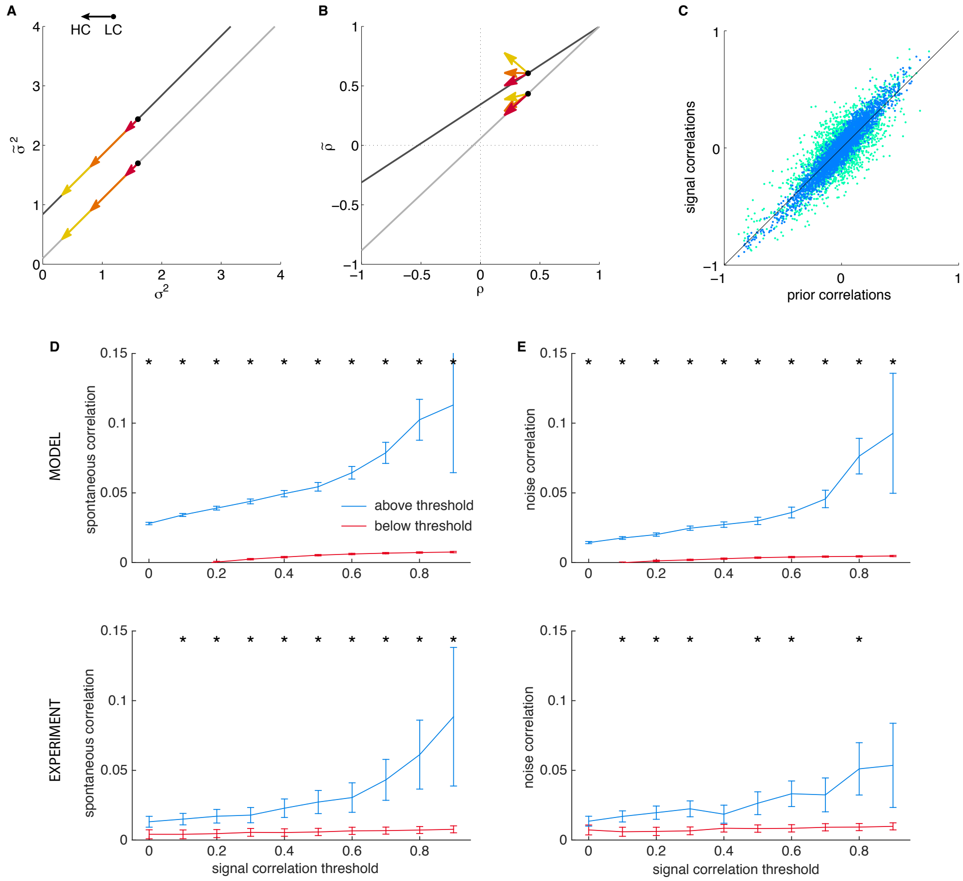

Figure S4 ***(continued on following page)***

Figure S4 *(preceding page).* Related to Figs. 4-6. Response correlations. (**A,B**) Effect of synchronized fluctuations on membrane potential variance and cross-correlation, respectively. A change from low contrast (LC) to high contrast (HC) reduces within-state membrane potential variance (σ^2) and correlation (ρ) which in turn cause changes in overall membrane potential variance ($\tilde{\sigma}^2$) and correlation ($\tilde{\rho}$). Arrows point from LC to HC values, different colors correspond to different magnitudes of within-state variance reduction (see below). Grey lines correspond to different state-variance magnitudes, $\omega^2 = 0.0975\,\mathrm{mV}^2$ (light gray) and $\omega^2 = 0.84\,\mathrm{mV}^2$ (dark gray). For illustration, based on Haider et al. (2013) reporting bimodal membrane potential distributions in anesthesia, a mixture of two Gaussians was used for modelling response distributions with the following parameters: fraction of low membrane potential state $p = 0.3$ (dark gray) or 0.025 (light gray), difference between high and low baseline membrane potential $\Delta_\mu = 2\,\mathrm{mV}$, $\sigma^2_{HC} = 1.6\,\mathrm{mV}^2$, $\rho_{HC} = 0.4$, $\sigma^2_{LC} = \sigma^2_{HC} \cdot [1.15, 2, 5]$ for red, orange, and yellow arrows, respectively, $\rho_{LC} = 0.2$. (**C**) Prior correlations in the model reflect signal correlations. Correlation between prior and signal membrane potential correlations across all pairs of neurons during natural image presentation was 0.95 (blue dots), and 0.79 when grating stimuli were used to compute signal correlations (green dots). (**D,E**) Robustness of the signal correlation-dependence of spontaneous (**D**) and noise correlations (**E**). Different thresholds for splitting signal correlations were tested (x-axis), lines show mean (±s.e.) spontaneous and noise correlations across cell pairs with above- (blue line) and below-threshold (red line) signal correlations, $^*p < 0.05$. Cf. **Fig. 6** of the main text, showing analysis of same data (for both model and experiments) with a threshold of 0.5.

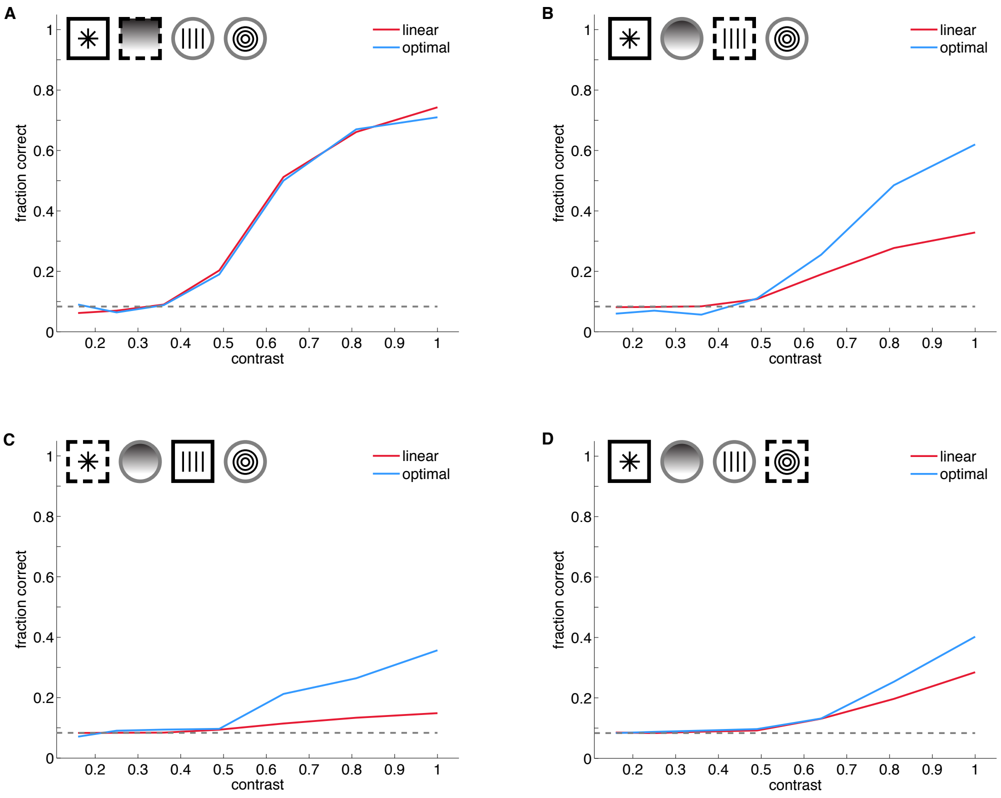

Figure S5. Related to Figs. 4-6. Decoding performance of a linear and an optimal decoder. Panels show four decoding tasks, differing in the role of four stimulus attributes (insets: orientation, contrast, phase, and aperture size of a sinusoidal grating stimulus). In each task, two attributes were kept fixed (icons in gray circles), and two varied (icons in black squares), out of which one had to be decoded (solid contour), and one ignored (nuisance parameter, dashed contour). (**A**) Decoder performance when the stimulus attribute to be decoded was orientation, and contrast needed to be ignored. Stimulus phase and aperture size were fixed. Dashed line denotes chance level. The linear decoder was trained using grating images of which the contrast was uniformly distributed among seven discrete levels, and test performance is shown at each contrast level separately. The optimal decoder (blue) was tested on the same stimuli as the linear decoder (red). (**B-D**) Similar to **B**, but with different stimulus attributes as the decoded and nuisance parameters.

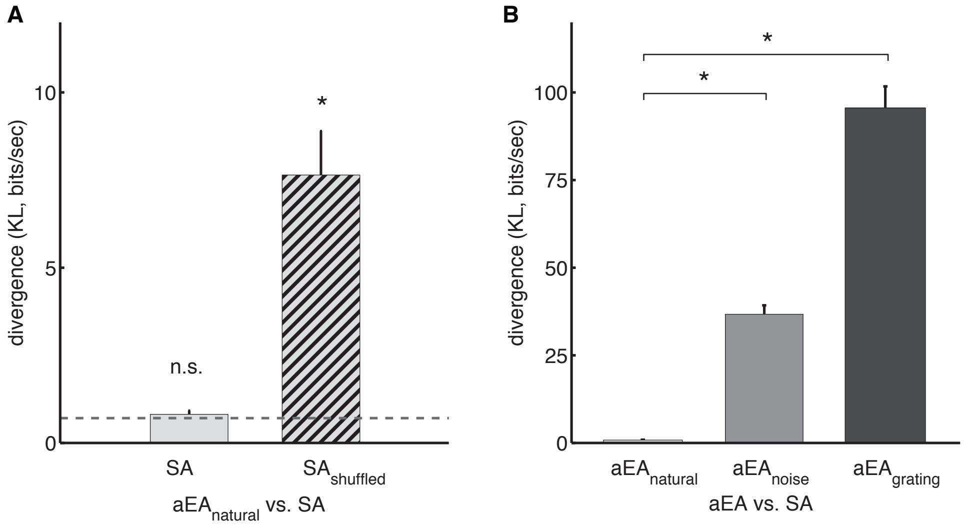

Figure S6. Related to Fig. 7. Match between spontaneous (SA) and average evoked activity (aEA) distributions in the model depends on correlations. (**A**) and the stimulus ensemble used (**B**). Well isolated units were simulated in which the spike count of each model neuron was separately recorded (c.f. **Fig. 7** of the main text, in which multiunit activity was simulated by combining the spike counts of several model neurons in each unit). (**A**) Kullback-Leibler (KL) between aEA for natural image patches ($\mathrm{aEA}_{\mathrm{natural}}$) and SA (light grey bar), and between $\mathrm{aEA}_{\mathrm{natural}}$ and a shuffled version of SA, preserving individual firing rates but destroying all correlations across electrodes ($\mathrm{SA}_{\mathrm{shuffled}}$, hatched bar). For reference, baseline KL divergence between two halves of SA data is also shown (dashed line). (**B**) KL divergence of SA from aEA under three different stimulus conditions: natural image patches ($\mathrm{aEA}_{\mathrm{natural}}$, light grey bar, same as in panel **A**), random block noise images ($\mathrm{aEA}_{\mathrm{noise}}$, dark grey bar), and grating stimuli with various phases, orientations, and frequencies ($\mathrm{aEA}_{\mathrm{grating}}$, black bar). In both panels, bars show averages across multiple simulations, error bars show s.e., $*p < 0.05$.
