# Supplementary material for: Neural Variability and Sampling-Based Probabilistic Representations in the Visual Cortex
Source: Neuron. 2016 Oct 19;92(2):530–43. doi: 10.1016/j.neuron.2016.09.038 (PMC5077700; doi:10.1016/j.neuron.2016.09.038)
Supplement: Document S2. Article plus Supplemental Information [file mmc2.pdf]

Article

Neuron

Neural Variability and Sampling-Based Probabilistic Representations in the Visual Cortex

Highlights

- Stochastic sampling links perceptual uncertainty to neural response variability
- Model accounts for independent changes in strength and variability of responses
- Model predicts relationship between noise, signal, and spontaneous correlations
- Stimulus statistics dependence of response statistics is explained

Authors

Gergő Orbán, Pietro Berkes, József Fiser, Máté Lengyel

Correspondence

orban.gergo@wigner.mta.hu

In Brief

Orbán et al. show that linking perceptual uncertainty to neuronal variability accounts for systematic changes in variability and covariability in simple cells of the primary visual cortex. The theory also establishes a formal relationship between signal, noise, and spontaneous correlations.

Orbán et al., 2016, Neuron *92*, 530–543
October 19, 2016 © 2016 The Author(s). Published by Elsevier Inc.
http://dx.doi.org/10.1016/j.neuron.2016.09.038

CellPress

Neuron
Article

Neural Variability and Sampling-Based Probabilistic Representations in the Visual Cortex

Gergő Orbán,[1,2,5,6,*] Pietro Berkes,[3] József Fiser,[3,4,5] and Máté Lengyel[1,4]
[1]Computational and Biological Learning Lab, Department of Engineering, University of Cambridge, Cambridge CB2 1PZ, UK
[2]MTA Wigner Research Center for Physics, Budapest 1121, Hungary
[3]Volen National Center for Complex Systems, Brandeis University, Waltham, MA 02454, USA
[4]Department of Cognitive Science, Central European University, Budapest 1051, Hungary
[5]Brain & Cognitive Sciences, University of Rochester, Rochester, NY 14627, USA
[6]Lead Contact
*Correspondence: orban.gergo@wigner.mta.hu
http://dx.doi.org/10.1016/j.neuron.2016.09.038

SUMMARY

Neural responses in the visual cortex are variable, and there is now an abundance of data characterizing how the magnitude and structure of this variability depends on the stimulus. Current theories of cortical computation fail to account for these data; they either ignore variability altogether or only model its unstructured Poisson-like aspects. We develop a theory in which the cortex performs probabilistic inference such that population activity patterns represent statistical samples from the inferred probability distribution. Our main prediction is that perceptual uncertainty is directly encoded by the variability, rather than the average, of cortical responses. Through direct comparisons to previously published data as well as original data analyses, we show that a sampling-based probabilistic representation accounts for the structure of noise, signal, and spontaneous response variability and correlations in the primary visual cortex. These results suggest a novel role for neural variability in cortical dynamics and computations.

INTRODUCTION

Neural responses in sensory cortices are notoriously variable: the same stimulus can evoke a different response on each presentation (Henry et al., 1973; Tomko and Crapper, 1974). While there have been great advances in characterizing the detailed patterns and statistical structure of cortical variability (Ecker et al., 2014; Goris et al., 2014; Kohn and Smith, 2005; Lin et al., 2015), its computational relevance has received far less attention. Indeed, the consequences of cortical variability have almost exclusively been studied from the perspective of neural coding, where variability is considered as pure noise or nuisance (Carandini, 2004; Moreno-Bote et al., 2014; Shadlen and Newsome, 1998; Tolhurst et al., 1983). Conversely, computational theories of cortical representations (Adelson and Bergen, 1985; Karklin and Lewicki, 2009; Olshausen and Field, 1996; Schwartz and Simoncelli, 2001) and dynamics (Churchland et al., 2012; Hennequin et al., 2014b; Mante et al., 2013; Rigotti et al., 2013; Rubin et al., 2015) focused only on trial-average responses, either ignoring variability altogether or considering only a simple scaling of variability with average responses (Ma et al., 2006).

Here, we argue that the rich structure of neural variability in sensory cortices reveals a key aspect of cortical computations: the representation of perceptual uncertainty. The need to represent uncertainty is the logical consequence of formalizing perception as unconscious inference (Helmholtz, 1962). For example, our retinal activations can have several different interpretations in terms of the composition and arrangement of objects in the environment, each being valid with a different probability. Thus, the uncertainty inherent in perceptual inference can be formalized as a probability distribution over possible perceptual interpretations of our input (Knill and Richards, 1996). The question is, then, how do neural activities represent probability distributions (Fiser et al., 2010)? We propose that probability distributions are directly represented by the variability of cortical responses.

To study the implications of representing uncertainty through neural variability, we developed a model of population responses in the primary visual cortex (V1) with three main assumptions. First, we posit that neural activity patterns represent statistical samples from a probability distribution over visual features of a scene (Fiser et al., 2010; Hoyer and Hyvarinen, 2003; Lee and Mumford, 2003). Second, we specifically propose that individual samples in the model are represented by the membrane potentials (or, equivalently, the instantaneous firing rates) of neurons. Third, as the autocorrelations of membrane potentials for any static stimulus typically decay on a relatively short ($\sim$20 ms) timescale (Azouz and Gray, 1999), membrane-potential values (and consequently firing rates) separated on this timescale are considered statistically independent and therefore are modeled as independent stochastic samples from the underlying probability distribution. This naturally gives rise to within- as well as across-trial variability in the model.

This proposed representational scheme has two main implications. First, the set of responses (i.e., membrane-potential values) at any time in a population of neurons in V1 represents a combination of visual features as a possible interpretation of

 © 2016 The Author(s). Published by Elsevier Inc.
This is an open access article under the CC BY license (http://creativecommons.org/licenses/by/4.0/).

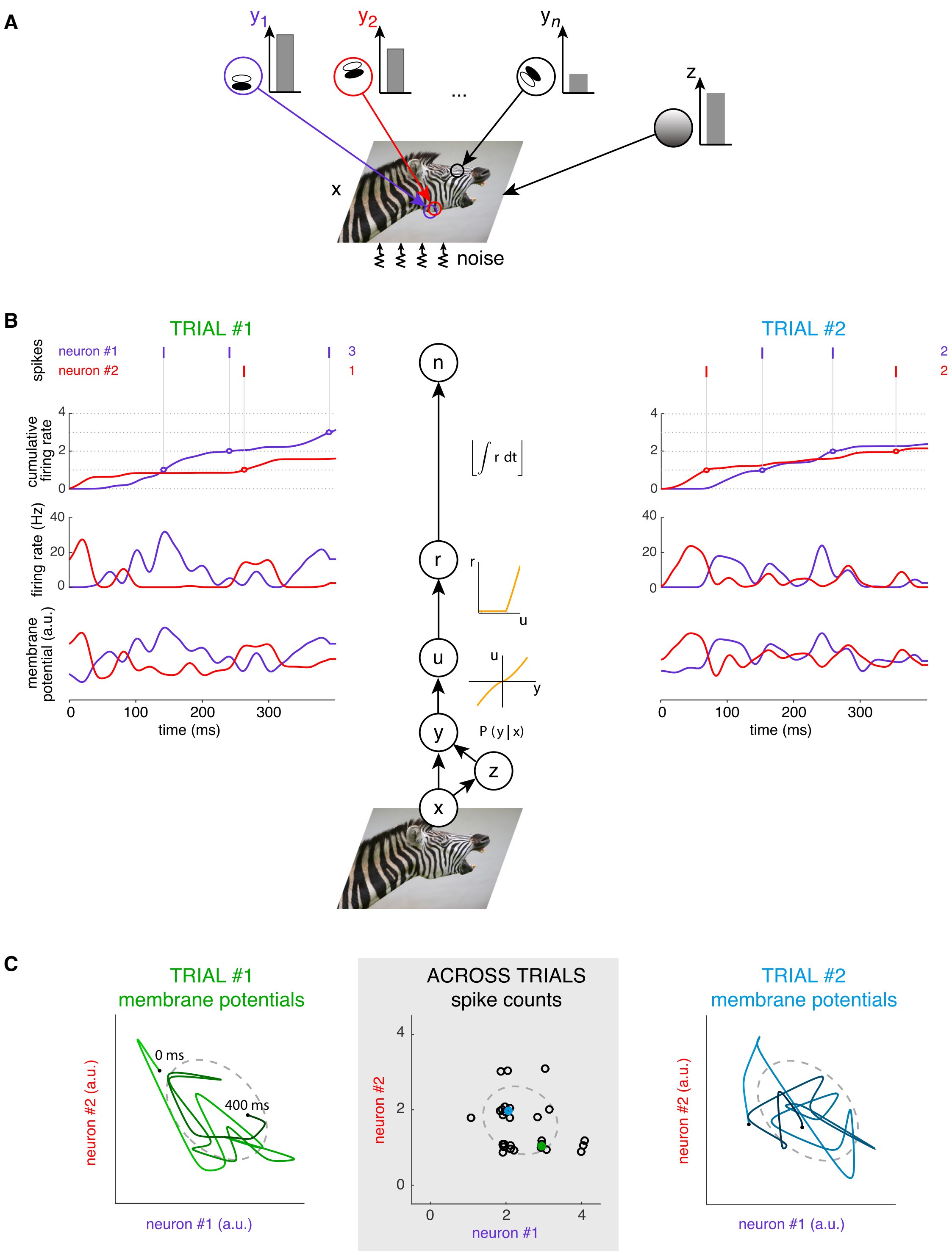

Figure 1. Schematic of the Model

(A) The generative model describing the statistical structure of image patches (**x**). Images arise as a linear combination of Gabor-filter basis functions with intensities $\mathbf{y} = \{y_1, \ldots, y_n\}$, whose contribution to the image is jointly scaled by a "contrast" variable, z, plus Gaussian white noise (see Experimental Procedures for details).

(B) Probabilistic inference and the generation of membrane potentials and spike counts. The progression of four steps in the model is shown in the middle of the panel, advancing from the bottom toward the top. The activations of two example cells in red and purple (see the corresponding basis functions in A) are illustrated in two different trials using the same stimulus, **x** (left and right sides in B). Basis function activations, **y**, are inferred by inverting the generative process shown in (A).

(legend continued on next page)

the input. Second, the within-trial variability of responses is such that the relative frequency with which any population pattern is visited is equal to the probability that the corresponding combination of features is a valid interpretation of the visual scene. Thus, neural response variability is directly linked to uncertainty about the stimulus: the wider the inferred range of possible feature combinations is, the wider the distribution of responses will become. In contrast to earlier proposals for how uncertainty may be represented in cortical activities (Deneve, 2008; Ma et al., 2006; Rao, 2004; Zemel et al., 1998), this establishes the mean and variability of responses as independent information channels, respectively encoding the mean and the associated uncertainty of the probability distribution over visual features. Importantly, these predictions about within-trial variability can also be tested in variability measured across trials that use the same stimulus and thus elicit the same probability distribution from which responses are sampled.

To test our model, we systematically compared the neural variability that our model predicted in response to various visual stimuli with the across-trial variability recorded in V1 in response to the same set of stimuli. As the parameters of our model were fundamentally determined by the statistical properties of visual scenes, rather than the properties of V1 circuits, this approach allowed a strong test of the model. Specifically, we show that the sampling-based representation of our model accounts for several key properties of response variability in V1. First, response variability not directly related to the stimulus can be so high that it dominates evoked responses (Arieli et al., 1996; Fiser et al., 2004; Vogels et al., 1989). Second, just as mean responses show systematic changes with particular attributes of the stimulus (as characterized by tuning curves), so does the variability of responses. In particular, experimental manipulations of image contrast or aperture (known to control perceptual uncertainty; Weiss et al., 2002) modulate the magnitude of variability largely independently from changes in mean responses (Churchland et al., 2010); conversely, changes in the orientation of the stimulus (which do not influence uncertainty) mainly affect the trial average of responses, and affect their relative variability much less. Third, response variability exhibits systematic patterns not only in its overall magnitude but also in its fine structure: signal correlations bear a specific relationship to noise (Ecker et al., 2010) and spontaneous correlations. Fourth, more generally, the structure of response variability during evoked activity closely resembles variability during spontaneous activity (Arieli et al., 1996; Berkes et al., 2011a; El Boustani et al., 2009; Fiser et al., 2004). In order to test and evaluate these implications of the model quantitatively, we compared model results directly to previously published experimental results whenever possible. To confirm the specific new predictions of the model about the structure and stimulus-dependent modulation of spike-count variability, we further performed novel analyses of a published dataset of V1 recordings from awake macaques (Ecker et al., 2010). These results suggest a new perspective on the functional role of variability in cortical dynamics and distinguish between previous conflicting proposals about how uncertainty is represented in the cortex.

RESULTS

From Natural Image Statistics to Neural Representations

We extended a well-known family of representational models of V1, in which the visual cortex maintains an internal model of how images are generated by underlying visual features (Figure 1A; see also Figure S1, Experimental Procedures, and Supplemental Experimental Procedures). According to this internal model, an image patch is generated by a multiplicative interaction between two terms (plus noise):

$$\text{image} = z \times \left(\sum_i \text{activation}_i \times \text{basis}_i\right) + \text{noise}. \qquad \text{(Equation 1)}$$

The first term, z, which we assumed for simplicity to be a single scalar, determines the global contrast level of the image patch. The second term is a linear combination of basis functions, and simple cell activations represent the coefficients with which each of these basis functions contribute to the image (Olshausen and Field, 1996; Schwartz and Simoncelli, 2001). In addition, the internal model also defines the prior probability distribution of basis function activations, P(activations), which expresses the frequency with which any combination of activations is expected to occur across different images. The role of V1 is then to invert this generative process and infer the level of activation for each feature in an image (Karklin and Lewicki, 2009; Olshausen and Field, 1996; Rao and Ballard, 1999; Schwartz and Simoncelli,

Due to noise and ambiguity in the model, **y** cannot be inferred from the image with certainty; hence, the result of Bayesian inference is a posterior probability distribution, $P(\mathbf{y}|\mathbf{x})$. Membrane-potential values, **u**, represent stochastic samples from $P(\mathbf{y}|\mathbf{x})$ through a weak non-linear transformation (inset), with independent samples drawn every ~20 ms, corresponding to typical autocorrelation timescales of V1 neurons (Azouz and Gray, 1999) (For illustration, membrane potentials are plotted after smoothing with a 7-ms Gaussian kernel here. See also Experimental Procedures). Instantaneous firing rates, **r**, are obtained from membrane potentials by a rectifying non-linearity (Carandini, 2004; inset). Spike counts are obtained by deterministically integrating firing rates across time over the duration of a trial: a spike is fired whenever the cumulative firing rate reaches an integer value (open circles on cumulative firing-rate traces and ticks in spike rasters, with the final spike counts shown at the right end of each raster). Note that while the distribution of neural responses (mean, variance, and covariance) remains unchanged across trials using the same stimulus, the actual time course of membrane potentials and the spike counts can vary substantially across trials due to stochastic sampling from the same underlying distribution.

(C) Statistics of the joint activity of a pair of neurons. The two sides show the membrane-potential trajectories of the pair of neurons in the two trials presented in (B) plotted against each other, revealing the higher-order statistics of the joint distribution (e.g., non-zero correlations). Colored lines correspond to the membrane-potential trajectories shown in (B) (color shade indicates elapsed time), and dashed gray ellipses show the covariance underlying the stochastic trajectories (identical for the two trials). The center shows joint spike-count distribution of the same two cells across a large set of trials (circles) for the same stimulus. The two colored circles correspond to the spike counts obtained from the two trials shown at the two sides and presented in (B). Small jitter was added to integer spike counts for illustration purposes. Photo is from Istock.com/CliffParnell.

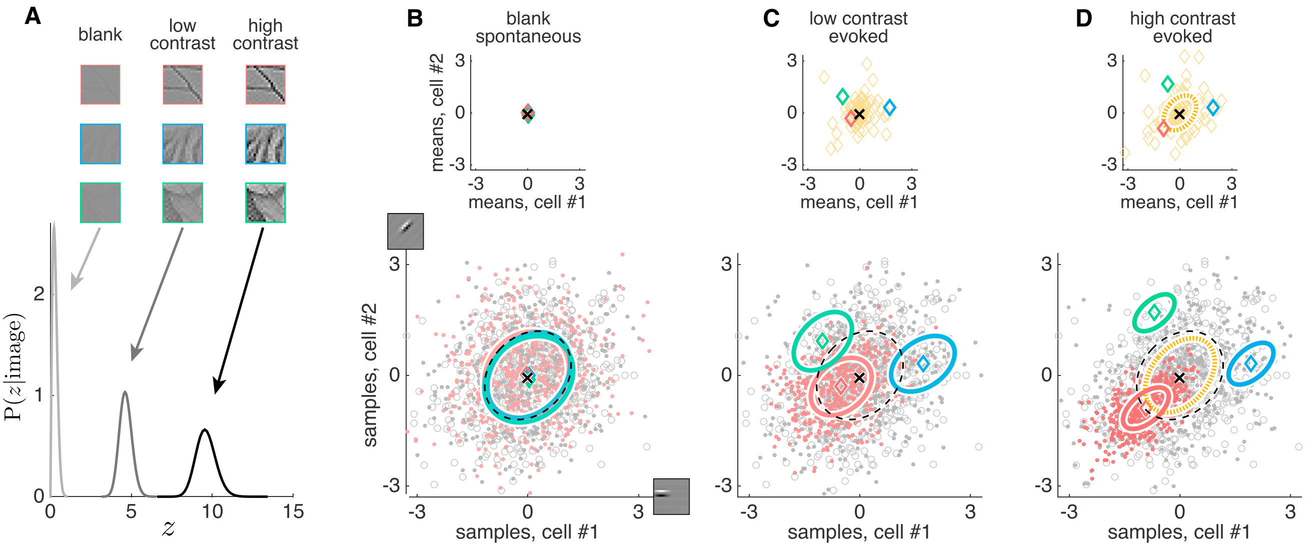

Figure 2. Key Features of Response Variability in Model Membrane Potentials

(A) Three example images (identified by the color of their frame) are shown at increasing contrast levels from left to right. Increasing the contrast shifts the posterior over the inferred contrast level, z, away from zero (gray distribution curves, from light to dark).

(B) Joint membrane-potential distributions of two example neurons (images at the end of axes show corresponding basis functions) for the three sample images in (A) at low contrast (colored diamonds, means; colored ellipses, covariances for the three images). Colors correspond to image frames in (A) compared to the prior distribution (black cross, mean; dashed black ellipse, covariance). The prior and the three posteriors strongly overlap; therefore, samples drawn from these distributions (gray circles, prior; red dots, posterior for image with red frame; gray dots, average posterior across 100 different images) and their means (crosses and diamonds) are indistinguishable. Inset on top shows the prior mean (black cross) and posterior means for the three natural image patches presented in (A) (colored diamonds).

(C and D) Shown as in (B), but for two higher contrast levels. The posteriors for the three images increasingly deviate from the prior and each other: their mean moves further away from zero while their covariances (noise covariances) shrink and remain similar. Signal covariance (yellow dotted ellipse in D) is aligned with the covariance of the prior (black dashed ellipse). Individual posteriors tile the subspace covered by the spontaneous covariance, such that samples drawn from the average posterior (gray dots), but not those drawn from any individual posterior (red dots), still overlap with those from the prior (gray circles). Insets on top show prior mean (black cross) and posterior means for the three images in (A) (red, green, and blue diamonds) as well as for 100 other natural image patches (yellow diamonds). In contrast to the decrease in noise covariances, signal covariances (covariances of posterior means across stimuli) increase with increasing contrast levels.

2001; Figure 1B, bottom; Experimental Procedures). The result of inference is a posterior distribution, P(activations | image), expressing the probability that any particular combination of features may underlie the current input.

Despite behavioral evidence for the representation of uncertainty (Ernst and Banks, 2002; Weiss et al., 2002), most previous representational models assumed that neural activities represent a single combination of features for each input (Karklin and Lewicki, 2009; Olshausen and Field, 1996; Rao and Ballard, 1999; Schwartz and Simoncelli, 2001), such as the one with the maximum posterior probability. These models were thus unable to capture the uncertainty expressed by the extent of the posterior. In contrast, our model maintained a representation of uncertainty by neural activities encoding randomly sampled feature combinations under the posterior. That is, the relative occurrence frequency of any neural activity pattern was equal to the inferred probability that the feature combination represented by it may have generated the input image. More specifically, we assumed that samples from the posterior were represented by the fluctuating membrane potentials of V1 cells through a weak compressing non-linearity, and we derived the instantaneous firing rate of a cell as a rectified-nonlinear function of its membrane potential (Carandini, 2004; Figure 1B, top; Supplemental Experimental Procedures). Thus, we took the membrane-potential values in a population of cells at any moment in time to represent a single sample from the multidimensional posterior, so that subsequent membrane potential values represented a sequence of samples (Figure 1C). This allowed us to make predictions about the form of the resulting distribution of neural activities in V1 without assuming a specific form for the underlying neural circuit dynamics.

Key Features of Neural Response Variability in the Model

Interpreting neural population activity patterns as samples from the posterior distribution of the internal model determined by Equation 1 establishes a direct link between the parameters of the posterior and the statistics of population responses. For example, the mean and the covariance of the posterior given a particular input image respectively correspond to the average and covariance of the neural responses evoked by that image. Thus, understanding the basic properties of the posterior distribution, and their dependence on the stimulus, provides key insights about the stimulus-dependent changes of cortical variability predicted by our model, which can be most directly demonstrated in the membrane-potential responses of a pair of model neurons (Figure 2).

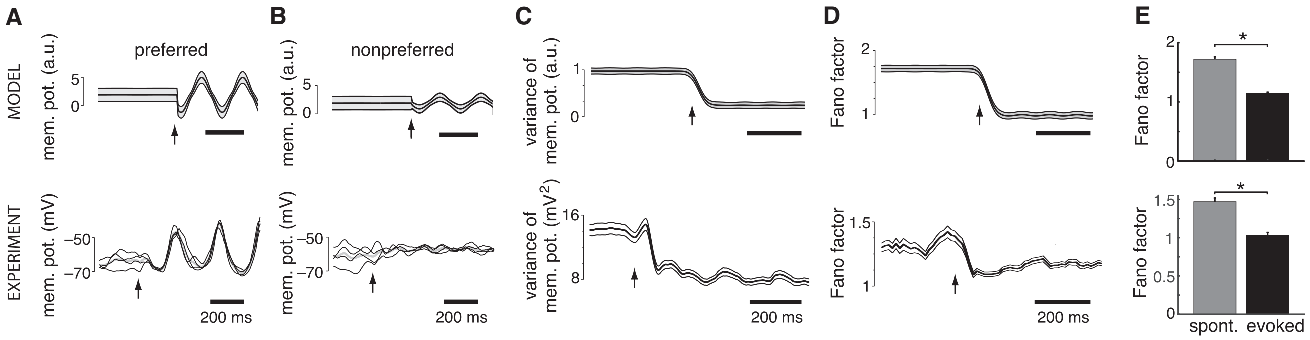

Figure 3. Stimulus Onset Quenches Neural Variability

(A and B) Periodic membrane-potential oscillations induced in an example neuron by a drifting sinusoid grating stimulus with preferred (A) and non-preferred (orthogonal to preferred) orientation (B) appearing after a blank image. Variability of responses is shown by their standard deviation (flanking gray area) for the model (top), and by individual trajectories in example trials (thin black lines) for the experimental data (bottom). Thick black (top) and gray (bottom) lines show across-trial average. Arrows mark stimulus onset.

(C and D) Population analysis of the effect of stimulus onset on the variance of membrane potentials (C) and the Fano factor of spike counts (D). Arrows mark stimulus onset; thick black lines and flanking thin lines show population average and SE.

(E) Direct comparison of spike-count Fano factors during spontaneous activity in response to a blank stimulus and evoked activity in response to high-contrast stimuli.

Bars show population average, error bars indicate 95% bootstrap confidence intervals, *$p < 0.05$. In each panel, the top plot shows the model results, and the bottom plot presents experimental data. Experimental data in (A)–(D) were reprinted by permission from Macmillan Publishers Ltd: Nature Neuroscience (Churchland et al., 2010, intracellular recordings in anesthetized cat). (E) presents an analysis of data from Ecker et al. (2010) (extracellular unit recordings in awake macaque). Fano factors in (D) and (E) were computed after mean matching (see Supplemental Experimental Procedures).

The variability of the average response of each cell across different stimuli is predicted by the dependence of the posterior mean on the image. As the basis functions in our model are oriented Gabor filters that are assumed to combine linearly in the image (Equation 1), the posterior mean of the activation of each basis function is largely determined by its linear overlap with the stimulus (Experimental Procedures; Equation 5). Thus, as in earlier models (Olshausen and Field, 1996), the trial-average response for simple oriented stimuli (such as commonly used full-field gratings) depends monotonically on the similarity of the "preferred orientation" of a cell (the orientation of its basis function) and the orientation of the stimulus, resulting in orientation-dependent tuning curves (Figure S2).

Changes in image contrast lead to corresponding changes in the inferred level of contrast, z (Figure 2A). A low-contrast image provides less evidence about the exact content of the image, so inferences rely predominantly on prior expectations, $\mathrm{P}(\text{activations})$. In the extreme case of a blank stimulus, z approaches zero (Figure 2A, light gray), so inferences about the basis function activations that neurons represent are unconstrained by the image (Equation 1 is constant with respect to the activations), and thus the posterior becomes entirely determined by the prior (Berkes et al., 2011a; Fiser et al., 2010). In other words, spontaneous activity, as a special case of evoked activity recorded in response to a blank stimulus, represents samples from the prior (Figure 2B).

For higher contrast levels, the inferred level of z also grows (Figure 2A, dark gray and black), so that the input image increasingly constrains the posterior of basis-function activations, which thus increasingly deviates from the prior (Figures 2C and 2D). This deviation has two major aspects. First, the mean of the posterior becomes different from the prior mean, and will be specific to the particular image that gave rise to it. This implies that signal variability, the variability of the mean response across different stimuli, grows with contrast (Figures 2B–2D, insets on top). Second, the observation of a high-contrast image reduces uncertainty (on average) about basis function activations relative to the prior. Thus, the (co)variance of individual posteriors will be smaller than that of the prior, implying that noise (co)variances, the across-trial variability of neural responses to the same stimulus, must decrease with increasing contrast (e.g., red covariance ellipses across Figures 2B–2D; see also Figures 3, 4A, 4B, and 5C). As opposed to the mean of the posterior (cf. Figure S2), its covariance does not show a strong dependence on the detailed content of the stimulus beyond its overall contrast (red versus green versus blue covariance ellipses within Figures 2B–2D; see also Figures 4C–4E). This is intuitive; for example, changing the orientation of a grating, as opposed to its contrast, does not influence our uncertainty about it.

As long as the internal model is well-adapted to the statistics of stimuli, it can be shown that its prior, $\mathrm{P}(\text{activations})$ (Figures 2B–2D, gray circles), must match the average posterior, $\langle \mathrm{P}(\text{activations} \mid \text{image}) \rangle_{\mathrm{P}(\text{image})}$, averaged across the distribution of stimuli, $\mathrm{P}(\text{image})$, to which it has been adapted (Gelman et al., 2013; Figures 2B–2D, gray dots). As for high-contrast images, noise variability in responses is low, but signal variability is high (see above; compare the size of the yellow covariance ellipse to that of the red-green-blue covariance ellipses in Figure 2D); most of the response variability is due to signal variability; and thus, spontaneous correlations (see above; reflecting the prior) are predicted to largely follow signal correlations (compare black dashed and yellow dotted covariance ellipses in Figure 2D; see also Figure 6A). As a consequence, we were also able to show in our model (Supplemental Experimental Procedures) that noise correlations will also be similar to signal correlations (compare the shape of the yellow covariance ellipse to red-green-blue

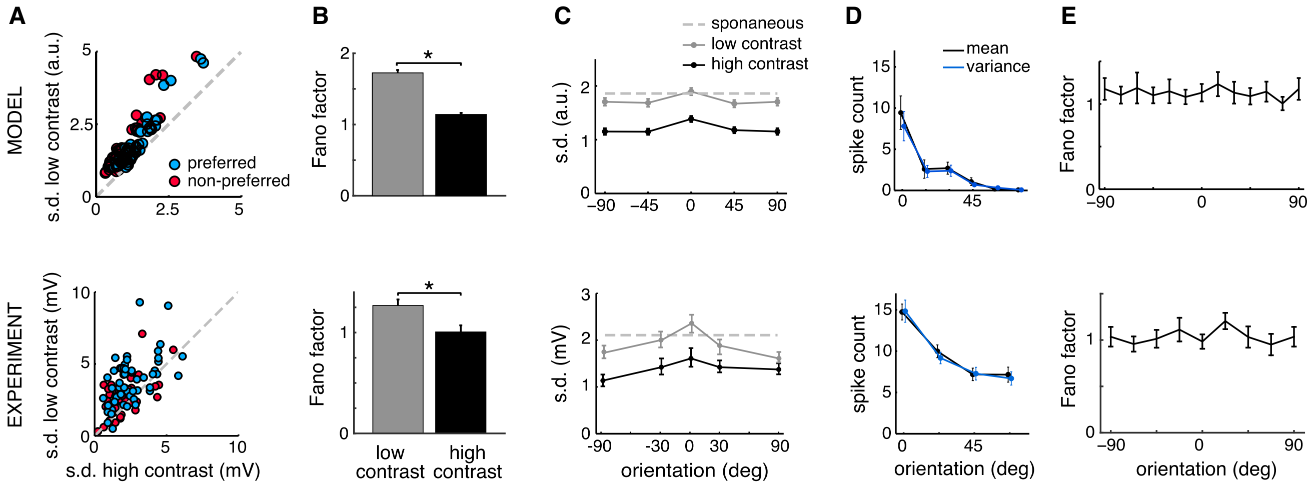

Figure 4. Stimulus Dependence of Neural Response Variability

(A) Across-trial SD of peak response amplitudes of a population of cells (circles) for low-contrast gratings plotted against the SD for high-contrast gratings at the preferred (blue) and non-preferred (red) stimulus orientation.

(B) Spike-count Fano factors (mean matched) for low- and high-contrast stimuli.

(C) Dependence of membrane potential SD on grating orientation at high (solid black line) and low (solid gray line) contrast. For reference, membrane potential SD during spontaneous activity recorded in response to a blank stimulus is also shown (dashed gray line).

(D and E) Mean and variance (black and blue lines in D) and Fano factor (E) of spike counts as a function of stimulus orientation relative to the preferred orientation of the cell.

(B)–(E) show population averages (bars or lines), with error bars showing 95% bootstrap confidence intervals (B) and SE (C)–(E), $*p < 0.05$. Experimental data in (A) and (C) were reproduced from Finn et al. (2007) with permission from Cell Press (intracellular recordings in anesthetized cat), and (B), (D), and (E) present analyses of data from Ecker et al. (2010) (extracellular unit recordings in awake macaque).

covariance ellipses in Figure 2D; see also Figure 6B). More generally, the matching of the average posterior to the prior predicts a match between the distribution of spontaneous activities and the average distribution of evoked activities (compare the scatter of empty and filled circles in Figures 2C and 2D; see also Figure 7) (Berkes et al., 2011a).

In the following, we test each of these key features of our model in neural data. For this, most parameters of the model were set according to the statistics of natural image patches, without regard to neural data, leaving only four free parameters to determine how sampled feature values under the posterior were mapped to membrane potentials and firing rates in V1 neurons (Experimental Procedures). Out of these four parameters, we determined one based on previous literature and tuned only three to fit specific experimental data recorded in V1. The experimental data to be reproduced were selected by a set of predetermined criteria regarding both the type of neural data recorded and the stimulus manipulations used in the experiments (Supplemental Experimental Procedures). Importantly, although these data included multiple species and conditions, we took a conservative approach and used a single setting of parameters across all our simulations (Table S1). For a fair comparison, in each case model responses were analyzed using the same statistical methods as those used for the analysis of the corresponding experimental dataset (Supplemental Experimental Procedures).

Mean Responses, Tuning Curves, and Contrast Invariance

In order to establish the validity of our model at a basic level, we first validated the model by reproducing some fundamental aspects of the mean responses of V1 simple cells. For this, we followed the method by which tuning curves are measured experimentally and computed average responses in the model for full-field grating stimuli with different orientations. As expected, our model neurons possessed clear orientation tuning for both membrane potentials and firing rates as found experimentally (Figures S2A and S2B). Importantly, despite the failure of previous attempts to reconcile sampling-based probabilistic representations with contrast invariant tuning curves (Pouget et al., 2013), firing-rate tuning curves in the model also showed contrast invariance (Skottun et al., 1987); i.e., only their amplitude scaled with contrast, but their width remained roughly constant (Supplemental Experimental Procedures; Figures S2B–S2E). This meant that, unlike models in which neuronal activity is proportional to probabilities (Pouget et al., 2013), our model did not suffer from the unrealistic property of tuning curves becoming exceedingly narrow at high-contrast levels, as high certainty was encoded by small noise variability instead (Figure 2). Moreover, our model also reproduced various characteristic non-classical receptive field (nCRF) effects, such as cross-orientation suppression and surround suppression (Bonds, 1989; Cavanaugh, 2001; Schwartz and Simoncelli, 2001; Supplemental Experimental Procedures; Figures S2F–S2H).

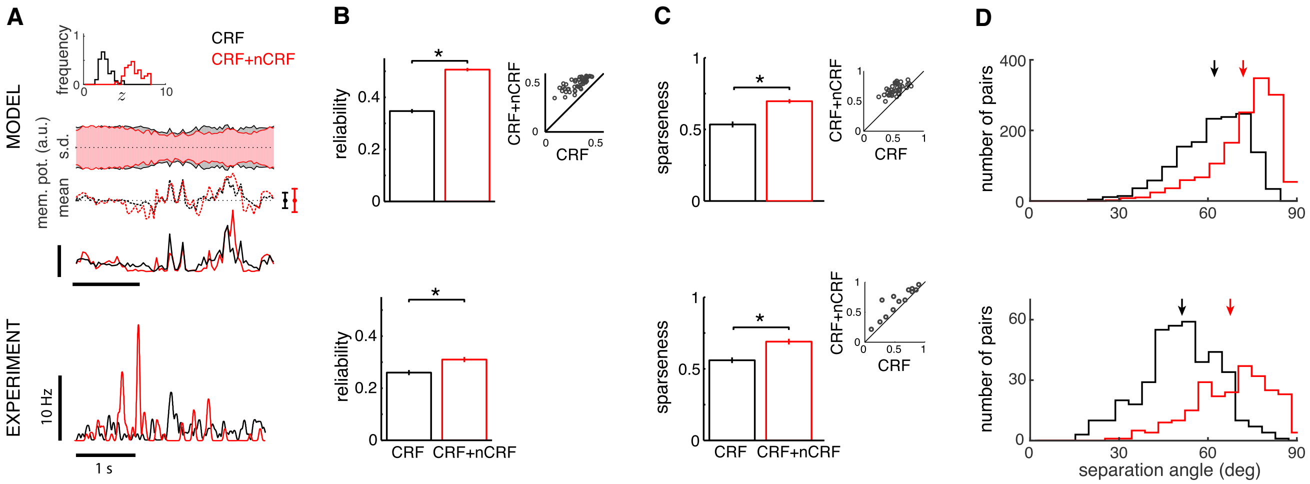

Figure 5. The Effect of Aperture on Response Reliability, Sparseness, and Signal Correlations

(A) The response of a representative neuron to repeated presentation of an image sequence constrained to the classical receptive field (CRF, black) or combined non-classical receptive field (nCRF) and CRF stimulation (CRF + nCRF, red). Model plots from top to bottom show distribution of inferred contrast levels, z, across frames of the stimulus movie (histograms); the SD (shaded area) and mean of the membrane potential (dotted lines, error bars to the right show signal variability); and the trial-average firing rate (solid lines) of a representative neuron across time. Experimental data show trial-average firing rate.

(B) Reliability of membrane-potential responses with CRF-only and combined nCRF + CRF stimulation. Inset (top) shows changes in the reliability for individual neurons.

(C) Lifetime sparseness of firing rates with CRF-only and combined nCRF + CRF stimulation. Insets show changes in sparseness for individual neurons.

(D) Distribution of separation angles between the mean response vectors of cell pairs with overlapping CRFs for CRF-only and combined nCRF + CRF stimulation. Arrows mark average separation angles. A higher separation angle means lower signal correlation.

(B) and (C) show population averages (bars or lines) with error bars showing SE, $^*p < 0.05$. Experimental data in (A)–(C) were reproduced from Haider et al. (2010) with permission from Cell Press (intracellular recordings in anesthetized cat), and those in (D) were reprinted from Vinje and Gallant (2000) with permission from AAAS (extracellular recording from awake macaque).

Response Variability and Stimulus Onset

The decrease in noise variability with contrast (Figure 2) in our model predicts that a high-contrast image following a blank period should lead to decreasing variability in V1 membrane-potential responses, and that this effect should hold regardless of whether or not the stimulus is aligned with the preferred orientation of a cell (Figures 3A–3C, top). Moreover, these changes in membrane-potential variability should carry over to changes in spike-count Fano factors even with the effects of changes in mean firing rates being factored out (Churchland et al., 2010; Figures 3D and 3E, top, two-sample t test, $n = 90$, $p < 10^{-4}$, $t[178] = -5.4$; Figure S3D; see also Supplemental Experimental Procedures). Such quenching of variability at stimulus onset is a general feature of cortical responses reported under a wide variety of experimental conditions (Churchland et al., 2010); in particular, it has been observed in recordings from V1 simple cells of anesthetized cats (Figures 3A–3C, bottom) and monkeys (Figure 3D, bottom). Furthermore, our analysis of recordings from awake macaques (Ecker et al., 2010) shows that this effect is also present in the awake V1 (Figure 3E, bottom, two-sample t test, $n = 800$, $p < 10^{-4}$, $t[1,598] = 37.3$).

Contrast and Orientation Dependence of Noise Variability

Behavioral studies indicate that stimulus contrast directly affects subjective uncertainty (Weiss et al., 2002). This is consistent with the inverse scaling of posterior (co)variances with contrast in the model, which in turn predicts a similar scaling of noise (co)variances in V1 responses (Figures 2B–2D). Indeed, our model generated systematically higher membrane-potential variances for low- versus high-contrast stimuli (Figure 4A, top; paired t test, $n = 61$, $t[60] = -6.02$, $p < 10^{-4}$, and $t[60] = -6.28$, $p < 10^{-4}$ for stimuli with preferred and non-preferred orientations, respectively). Once again, this difference between the variances at high and low contrast was present for preferred as well as non-preferred stimuli (Figure 4A, top). The same pattern of results had been obtained experimentally from V1-simple cells of anesthetized cats (Finn et al., 2007; Figure 4A, bottom). The decrease in model membrane-potential variability was also reflected in a decrease in spike-count Fano factors (mean matched, see Supplemental Experimental Procedures; Figure 4B, top; two-sample t test, $n = 102$, $t[200] = -4.32$, $p < 10^{-4}$). Our analysis of data recorded in awake-monkey V1 also showed a similar decrease in (mean matched) Fano factors with increasing contrast (Figure 4B; bottom; two-sample t test, $n = 800$, $t[1,598] = 37.3$, $p < 10^{-4}$), confirming that it could not be attributed to the confounding effects of anesthesia, in which slow, synchronized activity fluctuation can have a major impact on measures of variability (Ecker et al., 2014; Goris et al., 2014; see also Supplemental Experimental Procedures and Figure S4A). Moreover, at the same time that noise variability decreased with contrast in the model, signal variability increased (Figures 2 and S2)—in agreement with experimental data showing a general scaling of average membrane-potential and

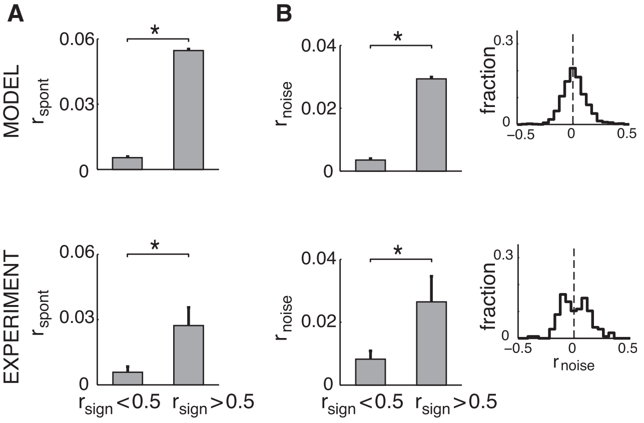

Figure 6. Relationship between Signal, Noise, and Spontaneous Correlations

(A) Dependence of correlations during spontaneous activity, r_{spont}, on spike-count signal correlations, r_{sign}.

(B) Dependence of noise correlations during evoked activity, r_{noise}, on signal correlations. Bars show averages across cell pairs with signal correlations below or above the r_{sign} = 0.5 threshold, as shown on the x axis; error bars show SE, *p < 0.05. Insets show the distribution of noise correlations; dashed line shows the mean of the distribution. Bottom panels present analyses of data from Ecker et al. (2010) (extracellular unit recordings in awake macaque).

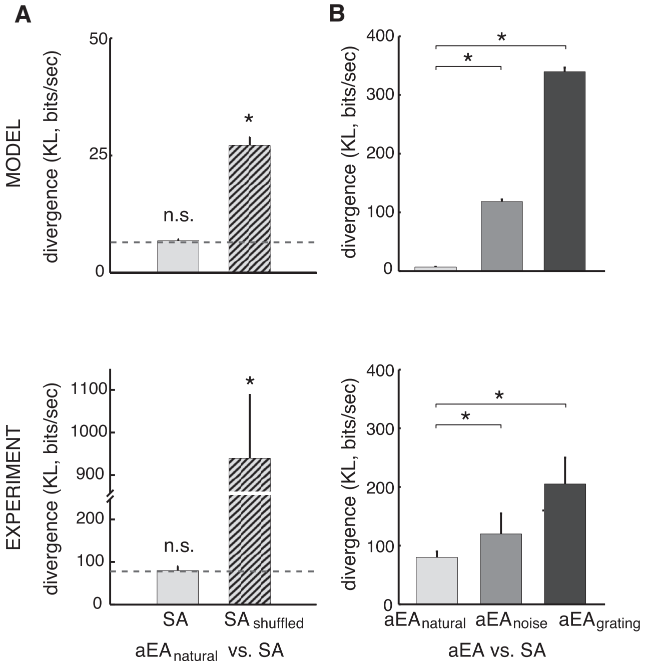

Figure 7. Match between Spontaneous and Average Evoked Activity Multi-Unit Distributions Depends on Correlations and the Stimulus Ensemble Used

(A) Kullback-Leibler (KL) divergence between aEA for natural image patches ($aEA_{natural}$) and SA (light gray bar), and between $aEA_{natural}$ and a shuffled version of SA, preserving individual firing rates but destroying all correlations across electrodes ($SA_{shuffled}$, hatched bar). For reference, baseline KL divergence between two halves of SA data is also shown (dashed line).

(B) KL divergence between aEA and SA under three different stimulus conditions: natural image patches ($aEA_{natural}$, light gray bar, same as in (A); random block noise images (aEA_{noise}, dark gray bar); and grating stimuli with various phases, orientations, and frequencies ($aEA_{grating}$, black bar). In all panels, bars show averages across animals and error bars show SE, *p < 0.05. Bottom panels present analyses of experimental data from Berkes et al. (2011a) with permission from AAAS (extracellular multi-unit recordings in awake ferrets).

firing-rate responses with contrast (Finn et al., 2007; Skottun et al., 1987), and in disagreement with a potentially simpler linear mechanism according to which both signal and noise variability would originate from the same form of contrast-dependent variability in the input (Moreno-Bote et al., 2014).

As opposed to contrast, the orientation of a stimulus primarily affects the mean estimate of how much the feature represented by a neuron contributes to the stimulus (reflected in the tuning curves of mean responses, Figure S2), and only much more moderately affects the uncertainty associated with this estimate (Figure 2, see also Supplemental Experimental Procedures). Confirming this observation, the membrane-potential variances in our model showed only mild modulation by stimulus orientation (Figure 4C, top). These results agreed with intracellular measurements showing a similar pattern of change in V1 simple cells of cats, with a small peak in the membrane-potential variance at the preferred stimulus orientations of neurons (Finn et al., 2007; Figure 4C, bottom).

The rectifying non-linearity that maps membrane potentials to firing rates in our model converted orientation-dependent changes in the mean membrane potential to changes in both the mean and the variance of spike counts (Figure 4D). However, as sampling resulted in the variance of membrane potentials remaining constant this time (as opposed to when contrast was changed, Figure 3), changes in spike-count variance were only as large as those in mean spike counts, such that the Fano factor of the spike-count distribution remained constant over the whole range of orientations (Figure 4E, top, one-way ANOVA $p = 0.98$, $F[11,108] = 0.30$). These predictions of the model have been confirmed by our analysis of awake-monkey recordings in V1 (Figures 4D and 4E, bottom, one-way ANOVA $p = 0.47$, $F[71,012] = 0.55$).

The Effect of Aperture on Response Variability, Sparseness, and Correlations

Although the generative process underlying our model specifies a relatively simple, largely linear mechanism for how natural image patches are generated as a combination of basic visual features (Equation 1; Figure 1A), inverting this process to infer the features from an image typically results in a complex posterior distribution that depends non-linearly on the image pixels. This complexity arises due to the so-called "explaining away" effect (Pearl, 1988), a common consequence of probabilistic inference, by which even distant pixel values that are unaffected by a visual feature under the generative process can indirectly influence the inferred value of that feature under the posterior. For example, in our model, all pixels in the image contribute to the inferred value of global contrast, *z*, which in turn influences the activity of all neurons (Figures 1 and 2), so even those portions of the image which are not part of the visual feature (basis function) represented by a neuron can change its activity.

As a result of explaining away, just as trial-average responses (tuning curves) were modified by suitable extra-classical

receptive field (eCRF) stimuli (see above and Figure S2), so too were the higher-order statistical moments of responses subject to such eCRF effects in our model. In particular, presenting the same natural movie sequence stimulus under a larger aperture that included both the classical receptive field (CRF) and the surround nCRF of a cell increased the effective contrast content of the input image (total variation in pixel values over the image), and thus led to a higher inferred value of z (Figure 5A, histograms). In other words, changes in aperture had effects on model inferences which were fundamentally analogous to changes in contrast (cf. Figure 2). In particular, just as when increasing contrast, an increase in inferred z resulted in higher signal variance and lower noise variance in membrane potentials (Figure 5A, dotted lines and shaded areas; cf. Figure 2) and thus more reliable membrane-potential responses (Figure 5B, top, one-sample t test, n = 54, t[53], 9.18, $p < 10^{-4}$). In turn, these opposite changes in signal and noise variability of membrane potentials meant that a larger fraction of the membrane-potential distribution of a cell lay respectively above or below the threshold for its preferred and non-preferred stimuli (frames of the movie). This increased the number of stimuli that evoked no firing in a cell while also increasing the firing rate for those stimuli that did evoke firing in it, and hence led to sparser spiking responses (Figures 5A, top solid line, and 5C, top, one-sample t test, n = 54, t[53] = −20.1, $p < 10^{-4}$). As the response of each neuron became sparser, these responses also became more de-correlated from each other, as reflected by the higher separation angles between the response vectors of neuron pairs with overlapping CRFs (Figure 5D, top, one-sample t test, n = 1,431, t[1,430] = −43.4, $p < 10^{-4}$). These results reproduced experimental data recorded in the anesthetized cat (Figures 5A–5C, bottom; Haider et al., 2010) and the awake monkey (Figure 5D, bottom; Vinje and Gallant, 2000) under similar stimulus manipulations. We found that the same mechanism also accounted for why phase scrambling of natural images, which decreased the overall local-contrast content of an image, led to less sparse responses in V1 in other experiments (Froudarakis et al., 2014; data not shown).

Next, we wanted to test whether the stimulus dependence (i.e., contrast and aperture dependence) of the variability of neural responses reproduced by our model (Figures 3–5) conveyed significant information about the stimulus beyond that information conveyed by mean responses. For this, we measured how well the stimulus could be decoded by taking into account or ignoring these stimulus-dependent variability modulations. We found that the decoding performance of an optimal decoder (which took all aspects of response distributions into account) was often substantially higher than that of a linear decoder (which assumed no changes in spike-count Fano factors; Figure S5; Supplemental Experimental Procedures). Thus, in contrast to other proposed population coding schemes for uncertainty (Ma et al., 2006), the sampling-based population code of our model was not linearly decodable in general.

Relationship between Signal, Spontaneous, and Noise Correlations

In the foregoing sections, we have demonstrated that the characteristics of the mean and the variance of individual model neuron responses in a sampling-based representation closely matched those found experimentally. In order to characterize the joint variability in the response distribution more completely, we also investigated the fine structure of correlations.

Our theory provided a principled link between various forms of response covariances and correlations during stimulus-evoked and spontaneous activity. In particular, it predicted a match between signal and spontaneous correlations as well as between signal and noise correlations (Figures 2 and S4C; see also Supplemental Experimental Procedures). Although these similarities were most cleanly predicted for membrane potentials, directly representing samples from the posterior distribution, they also carried over to firing rates and consequently to spike counts. In particular, we found a positive relationship between signal and spontaneous correlations of spike counts in the model (Figure 6A, top, two-sample t test, n = [27,232; 1,209], t[28,439] = −19.5, $p < 10^{-4}$), which was confirmed by our analysis of awake-monkey V1 recordings (Ecker et al., 2010; Figure 6A, bottom, two-sample t test, n = [1,474; 189], t[1,661] = −2.73, p = 0.0063). A similar relationship between spontaneous and signal correlations has also been noted in the anesthetized-cat V1, but it could not be captured by previous models (Lin et al., 2015). Spike-count noise correlations also had a positive relationship with signal correlations in the model (Figure 6B, top, two-sample t test n = [27,457; 1,223], t[28,678] = −12.0, $p < 10^{-4}$), in line with the general finding that noise and signal correlations tend to be positively related in a variety of cortical areas (Cohen and Maunsell, 2009; Gu et al., 2011) including the awake-macaque V1 (Ecker et al., 2010; Figure 6B, bottom, two-sample t test, n = [1,486; 172], t[1,656] = −2.20, p = 0.028). As our model neurons had a diverse set of receptive fields without a strong overrepresentation of any particular feature, the distribution of signal correlations was centered very close to zero (mean 0.015). As a corollary of the similarity of signal and noise correlations, the distribution of noise correlations also had a mean close to zero (Figure 6B, top inset mean 0.0074), in line with experimental findings in awake animals (Ecker et al., 2010; Figure 6B, bottom inset, mean 0.011).

Spontaneous and Evoked Response Distributions

In the previous sections, we have shown how a sampling-based representation accounted for differences in both neural variability and correlations between spontaneous and stimulus-evoked activities as responses recorded at zero and full contrast. However, sampling also implied specific similarities between spontaneous and stimulus-evoked activities (Figure 2D, bottom). In particular, it implied that the distribution of spontaneous activity (SA) must match the average distribution of evoked activities (aEAs). Importantly, this match was only expected to hold for stimuli to which the model has been adapted, i.e., for natural images but not for artificial images. Indeed, computing the dissimilarity between SA and the respective aEAs for natural images ($aEA_{natural}$), block noise patterns (aEA_{noise}), and drifting gratings ($aEA_{grating}$) confirmed these relationships in our model (Figures 7 and S6). More specifically, the divergence between $aEA_{natural}$ and SA was not different from a baseline divergence computed between the two halves of SA representing the minimal

divergence one could expect to see in the data (which was greater than zero due to finite sample size effects) (Figure 7A, top).

To test for the role of correlations for this match between $aEA_{natural}$ and SA, we independently shuffled the spike trains recorded on each electrode during spontaneous activity, thus preserving individual firing rates but destroying all correlations across electrodes (Berkes et al., 2011a; Fiser et al., 2013; $SA_{shuffled}$). This resulted in a divergence between $aEA_{natural}$ and $SA_{shuffled}$ that was significantly greater than baseline (Figure 7A, top, m-test, see also Berkes et al., 2011a, n = 20, m = 1.95e21, $p < 10^{-4}$) suggesting that the correlational structure of these activities, which we analyzed in the previous section, was crucial for the match between them. Extracellular recordings of multi-unit firing patterns in the V1 of awake adult ferrets (Berkes et al., 2011a) showed the same effect but with a greater magnitude (Figure 7A, bottom), possibly due to coordinated fluctuations in overall population activity during both SA and aEA (Fiser et al., 2013) that our model did not capture. Furthermore, the divergence between $aEA_{natural}$ and SA in the model was significantly smaller than the divergence between aEA_{noise} or $aEA_{grating}$ and SA (Figure 7B, top, m-test, see also Berkes et al., 2011a, n = 20, m = 9.15e42, $p < 10^{-4}$, and m = 2.97e55, $p < 10^{-4}$, respectively). This pattern of results was also observed in our ferret dataset: responses evoked by a natural movie ensemble showed less dissimilarity in distribution from spontaneous firing patterns than those evoked by grating stimuli or block noise (Berkes et al., 2011a; Figure 7B, bottom).

DISCUSSION

We presented a theory of the neural representation of uncertainty in the visual cortex that provides an account of a broad range of findings regarding neural variability in V1 which had previously lacked a unifying interpretation. Importantly, the model presented here is normative—it not only aims to capture the phenomenology of V1 activity but also proposes a rational, computational principle to explain why V1 should behave the way it does. In particular, the key principle of our model is that membrane-potential values (and hence firing rates) across a population of V1 neurons at subsequent moments in time are interpreted as samples drawn from a posterior distribution over visual features. This means that the variability of neural responses directly represents uncertainty about the visual image, such that higher uncertainty is reflected in increased noise variability of neural activity. This theory provided an intuitive explanation for why increasing contrast or aperture quenches variability in V1, and why stimulus orientation has little effect on it. The model also predicted the similarity of spontaneous and evoked activities and thus accounted for the finding that spontaneous, signal, and noise correlations tend to be correlated across cell pairs. To support these predictions, we presented analytical derivations and numerical simulations of the model as well as evidence from experimental recordings, including novel data analyses.

Distinguishing Different Probabilistic Representations

Our results provide a way to distinguish between previous conflicting proposals about the neural underpinning of probabilistic representations in the cortex (Fiser et al., 2010). These proposals broadly fall into two classes. In one class, both the mean of a probability distribution and the associated uncertainty are represented by time-average neural responses. In this class of models, changes in response variability are directly linked to changes in average responses and thus do not serve as an independent information channel (Deneve, 2008; Ma et al., 2006; Rao, 2004; Zemel et al., 1998). In the second class, which is based on sampling, the average and variability of responses encode different and complementary aspects of a probability distribution: average responses encode the mean, while variability and co-variability encode higher-order moments, such as variances and covariances, of the distribution (Fiser et al., 2010; Hoyer and Hyvarinen, 2003; Lee and Mumford, 2003). Therefore, the main empirically testable difference between sampling-based and most other types of probabilistic representations, such as probabilistic population codes (Ma et al., 2006), is that variability is controlled independently of mean responses in the former, while in the latter the mean and variance are coupled by a fixed constant of proportionality. Nevertheless, despite the fundamental differences in, e.g., how the mean and variability of responses are coupled in these two classes of models, no prior work attempted to link either of them directly to the rich structure of neural variability in sensory cortices.

We have shown here that a sampling-based representation correctly predicted that particular stimulus manipulations result in systematic, mean-independent modulations of variability in V1. Further analysis also revealed that these modulations of variability in the model, though they sometimes appeared to be subtle, in fact conveyed substantial amounts of information about the stimulus and thus could be expected to be functionally relevant for downstream computations (Supplemental Experimental Procedures; Figure S5). Crucially, models that couple response means and variances cannot capture these effects (Ma et al., 2006). Moreover, sampling also provided a parsimonious account of the similarity of noise, signal, and spontaneous correlations, as well as the similarity between evoked and spontaneous activities, which do not naturally emerge without additional assumptions in alternative models of probabilistic representations (Deneve, 2008; Ma et al., 2006; Rao, 2004; Zemel et al., 1998).

Key Model Assumptions

Our results were obtained by representing the result of inference over variables encoding basis function activations (Equation 1), and not those that encode contrast (z in Equation 1). This choice can be justified in two ways, both of which have precedents in previous representational models of V1 (Berkes et al., 2009; Karklin and Lewicki, 2009; Schwartz and Simoncelli, 2001). First, although such contrast variables are part of the generative model of natural images we considered, this does not imply that they also need to be explicitly included in the "recognition" model that the cortex uses to invert the generative model. Instead, they may be implicitly integrated out during inference. Note that even the posterior over basis function activations shows strong contrast dependence (both in its mean and covariance);

therefore, without an explicit representation of the contrast variable, contrast can be decoded from population activity should this decoding be necessary. Second, statistical arguments suggest that the number of contrast-like variables needs to be far lower than the number of those representing basis function activations, and so the experimental recordings which we use to test the theory are likely to be largely probing the latter. Nevertheless, were contrast-like variables represented explicitly in V1 and identifiable in experimental recordings (perhaps in inhibitory interneurons), we predict that their activity during spontaneous activity should not reflect the prior and, consequently, also should not match their average evoked-activity distribution.

In line with previous approaches (Karklin and Lewicki, 2009; Olshausen and Field, 1996; Schwartz and Simoncelli, 2001), our model took the posterior to be static compared to the timescale of inference, although under natural conditions, the posterior distribution itself may be changing due to both bottom-up and top-down effects. Bottom-up-driven changes in the posterior occur because the visual stimulus is changing, while top-down factors include changes in attention, cortical state (Goris et al., 2014; Harris and Thiele, 2011), and interactions with other sensory modalities (Driver and Noesselt, 2008). Thus, our results apply to standard visual electrophysiological experiments in which these factors are either well-controlled, by using the same stimulus and ensuring a homogeneous attentional state across multiple trials (Ecker et al., 2010), or averaged out, by pooling data over long time windows (Berkes et al., 2011a; Fiser et al., 2004). Furthermore, because the synchronized cortical state is characterized by large-amplitude fluctuations in membrane potentials and overall activity of cortical neurons, which are generally hard to control, our predictions are most directly testable in the desynchronized state characteristic of cortical populations processing the attended stimulus (Harris and Thiele, 2011; see also Supplemental Experimental Procedures and Figure S4).

Sampling and Neural Circuit Mechanisms

While our theory defines a neural representational scheme, it remains agnostic as to the neural circuit dynamics that give rise to such representations. As such, it accounts for the stationary distribution of neural network dynamics (as the posterior distribution that needs to be sampled) which is most readily testable in variability at slow timescales, e.g., across trials. However, anchoring the representation computationally in this way also provides useful constraints for mechanistic models that explicitly examine the underlying cellular- and network-level dynamics and thus make predictions about correlations at shorter timescales.

In particular, our model requires that the dynamically evolving membrane-potential or firing-rate traces of neurons represent sequences of stochastic samples from a posterior distribution. There have indeed been several neural circuit models proposed recently in which single neuron properties together with feedforward and recurrent connections shape either intrinsic or extrinsic noise in a network, such that for any particular input its dynamics produce samples from a computationally appropriate posterior distribution of activities (Buesing et al., 2011; Hennequin et al., 2014a; Savin et al., 2014). Such network models establish important proofs of the principle that neural circuit dynamics can give rise to sampling-based representations, and will be useful for making predictions about correlations on faster, within-trial timescales.

While the same stationary distribution can be attained by many different sampling algorithms, these will be different in their transient behaviors (so-called ‘‘burn-in’’) and non-equilibrium properties (i.e., whether and how they violate detailed balance), and so data about autocorrelations, including characteristic oscillations, fast timescale cross-correlations, and transients (Azouz and Gray, 1999; Ray and Maunsell, 2010), should reveal hallmarks of the specific sampling dynamics employed by the cortex (Hennequin et al., 2014a). For example, our preliminary results indicate that the stimulus-onset-related transients and the contrast-dependent oscillation frequency of V1 responses may be accounted for by a specific class of sampling-based neural circuit dynamics that is both computationally efficient and neurally plausible (Aitchison and Lengyel, 2014), in that it accommodates separate classes of excitatory and inhibitory neurons which most previous approaches eschewed (Buesing et al., 2011; Savin et al., 2014).

Sampling in Hierarchical Systems

Sampling-based representations lend themselves particularly naturally to self-consistent computations across multiple layers of a processing hierarchy ranging from low-level to high-level visual features, such as those found along the visual pathway (Lee and Mumford, 2003; Salakhutdinov and Hinton, 2012). Relating sampling in such hierarchically organized systems to neural variability along the cortical hierarchy should be able to capture various top-down effects in sensory processing that our simplified, non-hierarchical model could not address (Cohen and Maunsell, 2009; Kohn et al., 2009; Roelfsema et al., 2004). Indeed, recent results indicate that such a hierarchical sampling model can account for a variety of top-down task-related effects in visual cortical areas (Haefner et al., 2016). Moreover, our derivations for such a hierarchical extension not only reproduce all the main results of our simpler model, but they also predict that even images with equal contrast can evoke different amounts of response variability at both high and low levels of the hierarchy, depending on whether they afford higher-order percepts (Supplemental Experimental Procedures; Table S2). This is in line with recent experimental data comparing the sparseness and reliability of V1 responses to natural and phase-scrambled images (Froudarakis et al., 2014).

Note that hierarchical inference also obviates the need for an explicit, direct decoding of the posterior distribution from the samples, e.g., in the form of a histogram, as decision variables can be simultaneously inferred (and sampled from) together with lower-level variables. Moreover, both decision making and learning only require posteriors indirectly, through integrals of a cost function (Dayan and Abbott, 2005), thus implicitly implying a ‘‘smoothing’’ of samples. This smoothing mitigates the effects of the idiosyncratic ‘‘spiky’’ or ‘‘spaghetti-like’’ shape of sampling-based representations (e.g., in Figure 1C). More generally, non-linear effects in hierarchical inference can also explain how a relatively modest (less than a factor of 2) reduction in variance at the level of V1 following stimulus onset (Figure 3) can be

compatible with a drastic, orders-of-magnitude change in perceptual confidence (Supplemental Experimental Procedures; Figures S3E and S3F).

Sampling through Time

As inferences in our model are represented by sequentially generated samples at the rate of one new statistically independent sample every few tens (for membrane potentials) or hundreds of milliseconds (for spike counts), we expect this to limit the resolution of the representation of uncertainty. (Although, by using over-complete representations, in which many neurons effectively code for the same variable, even one sample of a population activity pattern may represent multiple samples of the relevant variables, such that the effective rate of sampling can be faster than expected from neural time constants; see, e.g., Savin and Deneve, 2014, and also Supplemental Experimental Procedures.) Indeed, such a gradual buildup of the representation of uncertainty over time within individual trials has been recently described (Lengyel et al., 2015). Moreover, it has been suggested that human-level performance in a range of behavioral tasks is indeed achievable by collecting a limited number of samples from a probability distribution given either static (P. Berkes et al., 2011b, COSYNE, conference; Vul et al., 2009) or dynamic stimuli (Levy et al., 2009). It has also been shown that specific patterns of perceptual variability in bi-stable percepts can be directly accounted for by sampling-based dynamics (Moreno-Bote et al., 2011). Our work complements these behavioral results by identifying the neural signatures of a sampling-based representation in V1, and demonstrates that the structure of neural variability and covariability provides useful clues for understanding the underlying probabilistic computations and representations utilized by the cortex.

EXPERIMENTAL PROCEDURES

The Gaussian Scale Mixture Model

We used a Gaussian scale mixture (GSM) model (Wainwright and Simoncelli, 2000) to define a generative model of image patches (Figure 1A). Each patch was represented by a vector of pixel values $\mathbf{x}$ and assumed to be generated by a scaled, linear combination of features plus additive Gaussian white noise (see also Equation 1),

$$\mathrm{P}(\mathbf{x} \mid \mathbf{y}, z) = \mathcal{N}\left(\mathbf{x}; z\,\mathbf{A}\,\mathbf{y}, \sigma_x^2\,\mathbf{I}\right), \qquad \text{(Equation 2)}$$

where $\mathbf{y}$ describes the activation of features in $\mathbf{A}$ for that image, z is an independent variable scaling the output of these features, and σ_x^2 is the variance of observation noise independently affecting the intensity of every pixel of the image. The multiplicative interaction between z and the basis functions captures two important aspects of natural images: first, that the effective contribution of each basis function (its activation level, $\mathbf{y}$, multiplied by z) is sparsely distributed, and second, that the magnitude of basis-function contributions within the same local image patch tends to be correlated (Schwartz and Simoncelli, 2001).

The prior of activations was a multivariate normal distribution with a mean of zero and covariance matrix $\mathbf{C}$,

$$\mathrm{P}(\mathbf{y}) = \mathcal{N}(\mathbf{y}; 0, \mathbf{C}), \qquad \text{(Equation 3)}$$

and the prior distribution of the scale variable, $\mathrm{P}(z)$, was a Gamma distribution with parameters k and θ.

The posterior distribution over feature activations could be obtained in a closed form for the scale variable z and, conditioning on z, also for the feature activations $\mathbf{y}$,

$$\mathrm{P}(z \mid \mathbf{x}) \propto \mathrm{P}(z)\mathcal{N}\left(\mathbf{x}; 0, z^2\mathbf{ACA}^{\mathrm{T}} + \sigma_x^2\,\mathbf{I}\right) \qquad \text{(Equation 4)}$$

and

$$\mathrm{P}(\mathbf{y} \mid z, \mathbf{x}) = \mathcal{N}(\mathbf{y}; \mu(z, \mathbf{x}), \Sigma(z)), \qquad \text{(Equation 5)}$$

where the posterior mean and covariance of feature activations is

$$\Sigma(z) = \left(\mathbf{C}^{-1} + \frac{z^2}{\sigma_x^2}\mathbf{A}^{\mathrm{T}}\mathbf{A}\right)^{-1} \text{ and } \mu(z, \mathbf{x}) = \frac{z}{\sigma_x^2}\Sigma(z)\mathbf{A}^{\mathrm{T}}\,\mathbf{x}.$$

As it was not necessary to represent the posterior distribution of z explicitly, we marginalized over this variable in order to express $\mathrm{P}(\mathbf{y} \mid \mathbf{x}) = \int \mathrm{d}z\,\mathrm{P}(z \mid \mathbf{x})\mathrm{P}(\mathbf{y} \mid z, \mathbf{x})$.

Membrane potentials (dimensionless), $\mathbf{u}$, were taken to represent a weakly non-linear function of visual feature activations $\mathbf{y}$ (Figure 1B, bottom):

$$u_i = \mathrm{sign}(y_i)|y_i|^{\alpha}. \qquad \text{(Equation 6)}$$

Firing rates were generated by first sampling membrane-potential values and then transforming them using a standard, rectified non-linearity (Carandini, 2004) (Figure 1B, middle):

$$r_i = m(u_i - u_{\mathrm{thresh}})_+^{\beta}. \qquad \text{(Equation 7)}$$

For sampling consecutive firing-rate values, we approximated autocorrelation timescales by regarding the firing rate of a cell to be constant within each 20 ms time bin and independently sampling across bins. Spike counts, $\mathbf{n}$, were generated simply by integrating instantaneous firing rates over time, starting from a random value distributed uniformly between zero and one (Figure 1B, top). Spike counts were computed over trial durations that matched those used in the corresponding experiments.

See Supplemental Experimental Procedures for a justification of model choices and more details of the model, including the setting of parameters, criteria used to select relevant experimental data to test the model, and procedures for analyzing neural responses in the model and in experimental data. Code for the model is available at https://github.com/gergoorban/sampling_in_gsm.

SUPPLEMENTAL INFORMATION

Supplemental Information includes Supplemental Experimental Procedures, six figures, and two tables and can be found with this article online at http://dx.doi.org/10.1016/j.neuron.2016.09.038.

AUTHOR CONTRIBUTIONS

M.L. conceived the theoretical framework. G.O. and M.L. developed the model and conducted the mathematical analyses. G.O. performed the numerical simulations. G.O., P.B., J.F., and M.L. discussed the results and wrote the manuscript.

ACKNOWLEDGMENTS

We thank R. Turner and G. Hennequin for useful discussions; D. Wolpert, R. Aslin, and A. Ecker for comments on a previous version of the manuscript; and especially A. Ecker, P. Berens, M. Bethge, and A. Tolias for making their data publicly available. This work was supported by an EU-FP7 Marie Curie Intra-European Fellowship, a Lendület Award of the Hungarian Academy of Sciences (G.O.), the Swartz Foundation (P.B. and J.F.), the Swiss National Science Foundation (P.B.), the NSF (J.F.), EU-FP7 Marie Curie CIG (J.F.), and the Wellcome Trust (M.L.).

Received: March 22, 2016
Revised: July 27, 2016
Accepted: September 6, 2016
Published: October 19, 2016

REFERENCES

Adelson, E.H., and Bergen, J.R. (1985). Spatiotemporal energy models for the perception of motion. J. Opt. Soc. Am. A *2*, 284–299.

Aitchison, L., Lengyel, M. (2014). The Hamiltonian brain. arXiv preprint 1407.0973v2.

Arieli, A., Sterkin, A., Grinvald, A., and Aertsen, A. (1996). Dynamics of ongoing activity: explanation of the large variability in evoked cortical responses. Science *273*, 1868–1871.

Azouz, R., and Gray, C.M. (1999). Cellular mechanisms contributing to response variability of cortical neurons in vivo. J. Neurosci. *19*, 2209–2223.

Berkes, P., Turner, R.E., and Sahani, M. (2009). A structured model of video reproduces primary visual cortical organisation. PLoS Comput. Biol. *5*, e1000495.

Berkes, P., Orbán, G., Lengyel, M., and Fiser, J. (2011a). Spontaneous cortical activity reveals hallmarks of an optimal internal model of the environment. Science *331*, 83–87.

Bonds, A.B. (1989). Role of inhibition in the specification of orientation selectivity of cells in the cat striate cortex. Vis. Neurosci. *2*, 41–55.

El Boustani, S., Marre, O., Béhuret, S., Baudot, P., Yger, P., Bal, T., Destexhe, A., and Frégnac, Y. (2009). Network-state modulation of power-law frequency-scaling in visual cortical neurons. PLoS Comput. Biol. *5*, e1000519.

Buesing, L., Bill, J., Nessler, B., and Maass, W. (2011). Neural dynamics as sampling: a model for stochastic computation in recurrent networks of spiking neurons. PLoS Comput. Biol. *7*, e1002211.

Carandini, M. (2004). Amplification of trial-to-trial response variability by neurons in visual cortex. PLoS Biol. *2*, E264.

Cavanaugh, J.R. (2001). Properties of the receptive field surround in macaque primary visual cortex. PhD thesis (Center for Neural Science, New York University).

Churchland, M.M., Yu, B.M., Cunningham, J.P., Sugrue, L.P., Cohen, M.R., Corrado, G.S., Newsome, W.T., Clark, A.M., Hosseini, P., Scott, B.B., et al. (2010). Stimulus onset quenches neural variability: a widespread cortical phenomenon. Nat. Neurosci. *13*, 369–378.

Churchland, M.M., Cunningham, J.P., Kaufman, M.T., Foster, J.D., Nuyujukian, P., Ryu, S.I., and Shenoy, K.V. (2012). Neural population dynamics during reaching. Nature *487*, 51–56.

Cohen, M.R., and Maunsell, J.H.R. (2009). Attention improves performance primarily by reducing interneuronal correlations. Nat. Neurosci. *12*, 1594–1600.

Dayan, P., and Abbott, L.F. (2005). Theoretical Neuroscience (MIT Press).

Deneve, S. (2008). Bayesian spiking neurons I: inference. Neural Comput. *20*, 91–117.

Driver, J., and Noesselt, T. (2008). Multisensory interplay reveals crossmodal influences on ''sensory-specific'' brain regions, neural responses, and judgments. Neuron *57*, 11–23.

Ecker, A.S., Berens, P., Keliris, G.A., Bethge, M., Logothetis, N.K., and Tolias, A.S. (2010). Decorrelated neuronal firing in cortical microcircuits. Science *327*, 584–587.

Ecker, A.S., Berens, P., Cotton, R.J., Subramaniyan, M., Denfield, G.H., Cadwell, C.R., Smirnakis, S.M., Bethge, M., and Tolias, A.S. (2014). State dependence of noise correlations in macaque primary visual cortex. Neuron *82*, 235–248.

Ernst, M.O., and Banks, M.S. (2002). Humans integrate visual and haptic information in a statistically optimal fashion. Nature *415*, 429–433.

Finn, I.M., Priebe, N.J., and Ferster, D. (2007). The emergence of contrast-invariant orientation tuning in simple cells of cat visual cortex. Neuron *54*, 137–152.

Fiser, J., Chiu, C., and Weliky, M. (2004). Small modulation of ongoing cortical dynamics by sensory input during natural vision. Nature *431*, 573–578.

Fiser, J., Berkes, P., Orbán, G., and Lengyel, M. (2010). Statistically optimal perception and learning: from behavior to neural representations. Trends Cogn. Sci. *14*, 119–130.

Fiser, J., Lengyel, M., Savin, C., Orbán, G., and Berkes, P. (2013). How (not) to assess the importance of correlations for the matching of spontaneous and evoked activity. arXiv preprint arXiv:1301.6554.

Froudarakis, E., Berens, P., Ecker, A.S., Cotton, R.J., Sinz, F.H., Yatsenko, D., Saggau, P., Bethge, M., and Tolias, A.S. (2014). Population code in mouse V1 facilitates readout of natural scenes through increased sparseness. Nat. Neurosci. *17*, 851–857.

Gelman, A., Carlin, J.B., Stern, H.S., Dunson, D.B., Vehtari, A., and Rubin, D.B. (2013). Bayesian Data Analysis, Third Edition (CRC Press).

Goris, R.L.T., Movshon, J.A., and Simoncelli, E.P. (2014). Partitioning neuronal variability. Nat. Neurosci. *17*, 858–865.

Gu, Y., Liu, S., Fetsch, C.R., Yang, Y., Fok, S., Sunkara, A., DeAngelis, G.C., and Angelaki, D.E. (2011). Perceptual learning reduces interneuronal correlations in macaque visual cortex. Neuron *71*, 750–761.

Haefner, R.M., Berkes, P., and Fiser, J. (2016). Perceptual decision-making as probabilistic inference by neural sampling. Neuron *90*, 649–660.

Haider, B., Krause, M.R., Duque, A., Yu, Y., Touryan, J., Mazer, J.A., and McCormick, D.A. (2010). Synaptic and network mechanisms of sparse and reliable visual cortical activity during nonclassical receptive field stimulation. Neuron *65*, 107–121.

Harris, K.D., and Thiele, A. (2011). Cortical state and attention. Nat. Rev. Neurosci. *12*, 509–523.

Helmholtz, H.L.F. (1962). Treatise on Physiological Optics (Dovwe).

Hennequin, G., Aitchison, L., and Lengyel, M. (2014a). Fast sampling-based inference in balanced neuronal networks. In Advances in Neural Information Processing Systems, *Volume 27*, Z. Ghahramani, M. Welling, C. Cortes, N.D. Lawrence, and K.Q. Weinberger, eds. (Curran Associates, Inc.), pp. 2240–2248.

Hennequin, G., Vogels, T.P., and Gerstner, W. (2014b). Optimal control of transient dynamics in balanced networks supports generation of complex movements. Neuron *82*, 1394–1406.

Henry, G.H., Bishop, P.O., Tupper, R.M., and Dreher, B. (1973). Orientation specificity and response variability of cells in the striate cortex. Vision Res. *13*, 1771–1779.

Hoyer, P., and Hyvarinen, A. (2003). Interpreting neural response variability as Monte Carlo sampling from the posterior. In Advances in Neural Information Processing Systems, *Volume 16*, S. Becker, S. Thrun, and K. Obermayer, eds. (MIT Press), pp. 293–300.

Karklin, Y., and Lewicki, M.S. (2009). Emergence of complex cell properties by learning to generalize in natural scenes. Nature *457*, 83–86.

Knill, D.C., and Richards, W. (1996). Perception as Bayesian Inference (Cambridge University Press).

Kohn, A., and Smith, M.A. (2005). Stimulus dependence of neuronal correlation in primary visual cortex of the macaque. J. Neurosci. *25*, 3661–3673.

Kohn, A., Zandvakili, A., and Smith, M.A. (2009). Correlations and brain states: from electrophysiology to functional imaging. Curr. Opin. Neurobiol. *19*, 434–438.

Lee, T.S., and Mumford, D. (2003). Hierarchical Bayesian inference in the visual cortex. J. Opt. Soc. Am. A Opt. Image Sci. Vis. *20*, 1434–1448.

Lengyel, M., Koblinger, Á., Popović, M., and Fiser, J. (2015). On the role of time in perceptual decision making. arXiv:1502.03135.

Levy, R., Reali, F., and Griffiths, T.L. (2009). Modeling the effects of memory on human online sentence processing with particle filters. In Advances in Neural Information Processing Systems, *Volume 21*, D. Koller, D. . Schuurmans, Y. Bengio, and L. Bottou, eds. (MIT Press), pp. 937–944.

Lin, I.-C., Okun, M., Carandini, M., and Harris, K.D. (2015). The nature of shared cortical variability. Neuron *87*, 644–656.

Ma, W.J., Beck, J.M., Latham, P.E., and Pouget, A. (2006). Bayesian inference with probabilistic population codes. Nat. Neurosci. *9*, 1432–1438.

Mante, V., Sussillo, D., Shenoy, K.V., and Newsome, W.T. (2013). Context-dependent computation by recurrent dynamics in prefrontal cortex. Nature *503*, 78–84.

Moreno-Bote, R., Knill, D.C., and Pouget, A. (2011). Bayesian sampling in visual perception. Proc. Natl. Acad. Sci. USA *108*, 12491–12496.

Moreno-Bote, R., Beck, J., Kanitscheider, I., Pitkow, X., Latham, P., and Pouget, A. (2014). Information-limiting correlations. Nat. Neurosci. *17*, 1410–1417.

Olshausen, B.A., and Field, D.J. (1996). Emergence of simple-cell receptive field properties by learning a sparse code for natural images. Nature *381*, 607–609.

Pearl, J. (1988). Probabilistic Reasoning in Intelligent Systems (Morgan Kaufmann).

Pouget, A., Beck, J.M., Ma, W.J., and Latham, P.E. (2013). Probabilistic brains: knowns and unknowns. Nat. Neurosci. *16*, 1170–1178.

Rao, R.P.N. (2004). Bayesian computation in recurrent neural circuits. Neural Comput. *16*, 1–38.

Rao, R.P.N., and Ballard, D.H. (1999). Predictive coding in the visual cortex: a functional interpretation of some extra-classical receptive-field effects. Nat. Neurosci. *2*, 79–87.

Ray, S., and Maunsell, J.H.R. (2010). Differences in gamma frequencies across visual cortex restrict their possible use in computation. Neuron *67*, 885–896.

Rigotti, M., Barak, O., Warden, M.R., Wang, X.-J., Daw, N.D., Miller, E.K., and Fusi, S. (2013). The importance of mixed selectivity in complex cognitive tasks. Nature *497*, 585–590.

Roelfsema, P.R., Lamme, V.A.F., and Spekreijse, H. (2004). Synchrony and covariation of firing rates in the primary visual cortex during contour grouping. Nat. Neurosci. *7*, 982–991.

Rubin, D.B., Van Hooser, S.D., and Miller, K.D. (2015). The stabilized supralinear network: a unifying circuit motif underlying multi-input integration in sensory cortex. Neuron *85*, 402–417.

Salakhutdinov, R., and Hinton, G. (2012). An efficient learning procedure for deep Boltzmann machines. Neural Comput. *24*, 1967–2006.

Savin, C., and Deneve, S. (2014). Spatio-temporal representations of uncertainty in spiking neural networks. In Advances in Neural Information Processing Systems, *Volume 27*, Z. Ghahramani, M. Welling, C. Cortes, N.D. Lawrence, and K.Q. Weinberger, eds. (Curran Associates, Inc.), pp. 2024–2032.

Savin, C., Dayan, P., and Lengyel, M. (2014). Optimal recall from bounded metaplastic synapses: predicting functional adaptations in hippocampal area CA3. PLoS Comput. Biol. *10*, e1003489.

Schwartz, O., and Simoncelli, E.P. (2001). Natural signal statistics and sensory gain control. Nat. Neurosci. *4*, 819–825.

Shadlen, M.N., and Newsome, W.T. (1998). The variable discharge of cortical neurons: implications for connectivity, computation, and information coding. J. Neurosci. *18*, 3870–3896.

Skottun, B.C., Bradley, A., Sclar, G., Ohzawa, I., and Freeman, R.D. (1987). The effects of contrast on visual orientation and spatial frequency discrimination: a comparison of single cells and behavior. J. Neurophysiol. *57*, 773–786.

Tolhurst, D.J., Movshon, J.A., and Dean, A.F. (1983). The statistical reliability of signals in single neurons in cat and monkey visual cortex. Vision Res. *23*, 775–785.

Tomko, G.J., and Crapper, D.R. (1974). Neuronal variability: non-stationary responses to identical visual stimuli. Brain Res. *79*, 405–418.

Vinje, W.E., and Gallant, J.L. (2000). Sparse coding and decorrelation in primary visual cortex during natural vision. Science *287*, 1273–1276.

Vogels, R., Spileers, W., and Orban, G.A. (1989). The response variability of striate cortical neurons in the behaving monkey. Exp. Brain Res. *77*, 432–436.

Vul, E., Goodman, N.D., and Griffiths, T.L. (2009). One and done? Optimal decisions from very few samples. In Proceedings of the 31st Annual Conference of the Cognitive Science Society, N. Taatgen and H. van Rijn, eds., pp. 66–72.

Wainwright, M.J., and Simoncelli, E.P. (2000). Scale mixtures of Gaussians and the statistics of natural images. In Advances in Neural Information Processing Systems, *Volume 12*, S.A. Solla, T.K. Leen, and K.-R. Muller, eds. (MIT Press), pp. 855–861.

Weiss, Y., Simoncelli, E.P., and Adelson, E.H. (2002). Motion illusions as optimal percepts. Nat. Neurosci. *5*, 598–604.

Zemel, R.S.S., Dayan, P., and Pouget, A. (1998). Probabilistic interpretation of population codes. Neural Comput. *10*, 403–430.

Neuron, Volume *92*

Supplemental Information

Neural Variability and Sampling-Based Probabilistic Representations in the Visual Cortex

Gergő Orbán, Pietro Berkes, József Fiser, and Máté Lengyel

Neural variability and sampling-based probabilistic representations in the visual cortex – Supplemental Experimental Procedures –

Gergő Orbán, Pietro Berkes, József Fiser, Máté Lengyel

Contents

1 Model details 2
2 Data set selection criteria 5
3 Statistical measures of neural activity 7
4 Change of variables: membrane potential- and firing rate-based representations 12
5 Relation to earlier representational models and reproducing data about average responses 13
6 Establishing relationships between different forms of correlations . 17
7 Hierarchical inference and top-down influences on response variability in V1 21
8 The relevance of stimulus-dependent changes in neural variability: decoding stimuli from spike trains 26

List of Figures

S1 Model parameters 37
S2 Contrast-invariance and non-classical receptive field effects in a representative model neuron 39
S3 Response variability in the model: parameter dependence and implications for perceptual confidence 41
S4 Response correlations 43
S5 Decoding performance of a linear and an optimal decoder 44
S6 Match between spontaneous (SA) and average evoked activity (aEA) distributions in the model depends on correlations 45

1 Model details

Gaussian scale mixture model. The Gaussian scale mixture model is defined in Eqs. 2-5 in Experimental Procedures. We have chosen this widely used model as our starting point not only because its variants capture essential aspects of the statistics of natural images and thus demonstrated cutting-edge performance in image compression and denoising (Wainwright & Simoncelli, 2000; Portilla & Simoncelli, 2000), but also because it has provided a normative account of both electrophysiological data about average firing rates in V1 (Olshausen & Field, 1996; Schwartz & Simoncelli, 2001), as well as about behavioral data on low-level vision (e.g. the tilt illusion; Schwartz et al. (2009)). While the true generative process of natural images, including high-level objects and attributes, is certainly more complex than the GSM model we are using here, the representation of these features also goes well beyond the level of V1. Therefore, we regard the GSM as an appropriate model to investigate stimulus-related changes in the distribution of V1 responses as long as the stimulus manipulations are well captured by the low-level visual features represented in V1.

In order to obtain a posterior that is not dependent on z, marginalization was performed by integrating over the posterior distribution of z, $\mathrm{P}(\mathbf{y}|\mathbf{x}) = \int \mathrm{P}(\mathbf{y}|z, \mathbf{x})$ $\mathrm{P}(z|\mathbf{x})\,\mathrm{d}z$ (with $\mathrm{P}(\mathbf{y}|z, \mathbf{x})$ given by Eq. 5 in Experimental Procedures). Marginalization was approximated as follows: a range of z around the maximum a *posteriori* (MAP) estimate was discretized and the posterior at the discretized values of was evaluated, thus the posterior of $\mathbf{y}$ effectively became a mixture of a finite number of Gaussians. The number of mixture elements was 50 during learning and 1 for inference, with the MAP value used for z. Using a mixture instead of the MAP value for inference did not qualitatively affect our results. Due to the lack of top-down influences in our underlying GSM model (see above), the posteriors we computed did not express higher-level forms of uncertainty, e.g. resulting from bistable percepts or ambiguity at the object-level. Nevertheless, the model still captured one of the most important forms of ambiguity at the level of V1: a certain local contrast level in the image was congruent with a continuum of hypotheses about (localized) basis function activations and global contrast level. As we show in the Results, this fundamental source of uncertainty has direct consequences on the forms of neural variability that are implied by a sampling-based representation of uncertainty.

Neural responses in the model. Membrane potentials, $\mathbf{u}$, were obtained by a weakly nonlinear transformation from feature activations, $\mathbf{y}$ (Eq. 6 in Experimental Procedures). This can also be understood as a generative model in which the latent variables are the membrane potentials, $\mathbf{u}$, themselves, but their prior is not a multivariate Gaussian anymore, as for $\mathbf{y}$ (Eq. 3 in Experimental Procedures), but instead a multivariate Gaussian mapped through this nonlinearity (see also Section 4). In contrast to the non-kurtotic normal prior distribution of $\mathbf{y}$, the prior of $\mathbf{u}$ becomes sparse at α values larger than 1 (**Fig. S3**).

Membrane potentials were generated by sampling $\mathbf{y}$ directly from the posterior (see above) and transforming them into samples of by using Eq. 6 in Experimental Procedures (**Fig. 1B**). This way, we did not address the question of how the dynamics of a neural circuit would obtain samples from $\mathbf{u}$ (but see Büsing et al., 2011; Grabska-Barwinska et al., 2013; Hennequin et al., 2014; Savin et al., 2014). Although, in general, samples generated by such dynamics will be correlated, samples separated by intervals beyond the time scale over which autocorrelations decay (20-50 ms for membrane potentials, Azouz & Gray (1999), and spike trains, Berkes et al. (2011b), in V1) will effectively be uncorrelated. Therefore, for membrane potentials, we compared our results, based on obtaining independent samples from the posterior, to data describing variability across trials which is well beyond the decorrelation time scale. In practice, the statistics of membrane potential responses (their mean, variance, and covariance) were computed by performing the corresponding integrals of Eq. 6 in Experimental Procedures over the posterior of $\mathbf{y}$ using numerical quadrature rather than using the samples to compute Monte Carlo integrals.

Spike counts were obtained by feeding membrane potentials through the firing rate nonlinearity (Eq. 7 in Experimental Procedures) and integrating these instantaneous firing rates over time and finally generating spikes at integer values of the integral. This corresponded to a simple deterministic (non-leaky integrate and fire) spike generation process in which a spike is taken to be emitted deterministically whenever the integrated input reaches a fix threshold (**Fig. 1B**). (Note that only spike counts and not the exact timing of spikes were analyzed in this study.) This procedure can be shown to generate spikes with exactly the mean firing rate given by r, such that spike count variability is determined almost entirely by firing rate variability (with minimal additional variability due to randomness in the initial condition). This is in contrast to an inhomogeneous Poisson process, which

would add a substantial amount of spiking noise, increasing Fano factors by one on top of the variability due to variations in firing rates.

Model parameters and inputs. All results were obtained with a single set of parameters (except where explicitly noted to demonstrate robustness to changes in parameters, **Figs. S2-S3**). For the GSM, we obtained the variance of the observation noise, σ_{x}^2 (Eq. 1), and the prior covariance matrix of visual features, $\mathbf{C}$ (Eq. 3 in Experimental Procedures), by fitting the model to pseudo-whitened 16-by-16 pixel natural image patches (van Hateren, 1992) using the expectation-maximisation (EM) algorithm (Dempster et al., 1977) (**Fig. S1B**). The E-step of the algorithm consisted of computing the posterior distributions as described above, while the M-step was subdivided into three partial alternating steps each changing one (set of) parameter(s) of the model: σ_{x}^2, and the norm and direction of $\mathbf{C}$. The rest of the parameters were fixed at preset values: $k = 2$ and $\theta = 2$ for the prior of the scale variable z (the specific values of these parameters did not affect our results); and the matrix of features, $\mathbf{A}$ (Eq. 2 in Experimental Procedures), was a bank of Gabor filters (Schwartz & Simoncelli, 2001) generated using four orientations and four spatial frequencies, each with a size of 1.9 times the length scale and at 1, 9, 16, or 36 different spatial locations (for increasing spatial frequency), so that a nearly complete (dimensionality: 248) basis set was formed (**Fig. S1A**). (Maximum likelihood learning of $\mathbf{A}$ simultaneously with $\mathbf{C}$ would be underconstrained because the predictive density only depends on their product $\mathbf{ACA}^\mathsf{T}$.) The best-fit value of σ_{x}^2 (noise), relative to the product of the average of the diagonal elements of the matrix $\mathbf{ACA}^\mathsf{T}$ and the expected value of z^2 (signal), was such that the model attributed a 1.8 signal-to-noise ratio to the training images.

The four remaining parameters of the model, determining the mapping of latent variables $\mathbf{y}$ to biological quantities (membrane potentials, firing rates, and spike counts), were determined based on experimental data. The exponent of the membrane potential nonlinearity (Eq. 6 in Experimental Procedures) was fitted to the orientation dependence of membrane potential variability (Finn et al., 2007) ($\alpha = 1.4$, see also Section 4). Following Carandini (2004), the exponent of the firing rate nonlinearity (Eq. 7 in Experimental Procedures) was set to be $\beta = 1.1$. As changes of the other two parameters of the firing rate nonlinearity, m and u_{thresh} (Eq. 7 in Experimental Procedures), could be compensated for by appropriate changes in the prior mean of $\mathbf{u}$ and the scale of $\mathbf{A}$, once those parameters were set (see above), we used these two parameters to fit the physiological range

of firing rate data: $m = 10\,\mathrm{Hz}$ and $u_{\mathrm{thresh}} = 1.9$. This both provided physiological firing rates (Carandini, 2004) in the model and set the level of spike count Fano factors (**Fig. 3D**, top) and variability (**Fig. 4D**, top) such that they matched experimentally measured levels (**Fig. 3C**, bottom, **4D**, bottom, see also **Fig. S3D**). (Note that no spike count correlation data was considered for these fits.)

Stimuli used to investigate response statistics of neurons in the simulations were chosen to match the statistics of stimuli used in the corresponding experiments. More specifically, the generation of natural image patches was identical to that used for generating training images (see above) (**Figs. 2, 5, 6, S6**). Grating images used either static (**Figs. 3E, 4B,D,E, 6** insets, **6b, S4C-E**) or drifting full-field sinusoidal gratings (**Figs. 3A-D, 4A,C, 6, S2, S3**) at 12 different orientations and 12 different phases. Random noise images were $2{\times}2$ binary block noise image patches (**Figs. 7B, S6**). Following Haider et al. (2010), natural image movie sequences (**Fig. 5**) were created by sampling a larger natural image along a linear trajectory. CRF and nCRF stimulation corresponded respectively to stimulating only the central 11 pixel-diameter part of the input, or all 16-by-16 pixels. In all these cases we simply modelled responses based on independent samples for each 20 ms time bin conditioned on the corresponding snapshot of the stimulus.

2 Data set selection criteria

We selected the experimental data sets we modeled based on two sets of criteria.

Neural data. Whenever possible, we used data sets in which membrane potentials were (also) recorded with their variance (or, as a proxy, their reliability) quantified, because these provided the most direct test of the theory. However, this had the disadvantage of almost always being recorded in anesthetised animals in which variability can show different patterns from awake animals (Ecker et al., 2014, see also below), and that with very few exceptions only one neuron is recorded at a time thus preventing the measurement of noise covariability. Therefore, we used spike count data recorded in the awake animal to demonstrate that the main results are also borne out in the awake state and specifically to analyse data on covariability which is particularly sensitive to effects of anesthesia (see below). For this, we compared to previous work in which changes in spike count variability – our main

interest (eg. change of Fano factor with contrast) – were analysed (Churchland et al., 2010). As most other published papers on spike counts did not perform such analyses, we also included previous work for which the original data was made publicly available (Ecker et al., 2010) or was our own (Berkes et al., 2011a) so that we could perform these analyses ourselves.

Stimulus manipulations. We chose data sets in which stimulus manipulations relevant for testing the main predictions of the model were carried out: orientation, contrast, aperture, natural vs. artificial stimulus ensembles. Wherever possible, we used data obtained with static stimuli because the generative process (the Gaussian scale mixture, GSM) underlying our model was defined for such inputs (although it is certainly possible to extend it to dynamic inputs). We made exceptions and used dynamic stimuli when such data were not available (membrane potential recordings and experiments investigating the effect of aperture), and for analyses involving signal correlations to improve the quality of their estimation (see Section 3).

Based on these criteria, the data sets used in the studies listed in Table S1 were the only appropriate ones.

Excluding anesthetised data from the analysis of correlations

For comparing correlations in our model to experimental data, we used only data recorded in awake animals. This is because commonly used forms of anesthesia can introduce coordinated, stimulus-independent fluctuations in the responses of cortical populations which can inflate both variances and correlations of neural responses (Ecker et al., 2014; Goris et al., 2014). Despite the effects of these fluctuations on the absolute magnitude of single neuron variability, as we show below they are not expected to qualitatively affect relative stimulus-dependent changes in variability, ie. increasing contrast should still decrease variance (**Fig. S4A**). Thus, it was possible to include anesthetized data for analyzing stimulus-dependent changes in variances and Fano factors in the preceding sections. Indeed, all our main predictions regarding single neuron variability were confirmed not only by anesthetized but also by awake recordings (see **Figs. 3-5** of the main text). In contrast, we show below that the same fluctuations, depending on their relative magnitude, can have more complex effects on changes in correlations (Goris et al.,

2014) whereby they can even revert the direction of correlation changes that would be seen in the awake animal in the absence of these fluctuations (**Fig. S4B**). For these reasons, we excluded anesthetized data from the analyses of correlations and joint pattern statistics (**Figs. 6-7** of the main text).

In order to estimate the effect of synchronized activity fluctuations on membrane potential variance and correlations, we constructed a simple model that describes the membrane potential of a pair of neurons as the sum of two factors: a baseline that is identical and shared across the two neurons and undergoes fluctuations with variance ω^2, and a neuron-specific 'private' component which has variance σ^2 in both neurons and (noise) correlation ρ across the two neurons. (A similar additive interaction between population-wide shared and single neuron-specific private variability has been found to provide a good fit to the overall variability in firing rates of V1 neurons in the awake macaque; Ecker et al., 2014.) With these assumptions, the overall variance of neural activities is the sum of the variances of the two contributing factors:

$$\tilde{\sigma}^2 = \sigma^2 + \omega^2 \tag{S1}$$

and the overall correlation between the two neurons, which includes the effects of synchronized baseline fluctuations, will be:

$$\tilde{\rho} = \frac{\rho\,\sigma^2 + \omega^2}{\sigma^2 + \omega^2} \tag{S2}$$

To see how our model's predictions transfer to this case, we consider that increasing contrast decreases private variance, σ^2, and the underlying correlation, ρ, but does not affect shared variance, ω^2. In this case, it is easy to see that single neuron variability, $\tilde{\sigma}^2$, will decrease irrespective of the magnitude of shared variance (**Fig. S4A**), ie. our main prediction carries over. In contrast, pairwise correlations, $\tilde{\rho}$, may decrease or increase, depending on the interaction of three factors: the magnitude of private variance reduction, correlation reduction, and the shared noise variance (**Fig. S4B**).

3 Statistical measures of neural activity

In order to ensure a fair comparison, responses generated by the model were analyzed in the same way as the corresponding experimental data (either already

published or our own analysis see below). The size of the model network was chosen based on computational considerations. For panels showing analysis of model results either all cells from the network were used, or cells were included following the same criteria as those used for the inclusion of cells in the corresponding experiments. Number of "animals" in **Fig. 7** were the same as in the corresponding experiments (Berkes et al., 2011a). Statistical tests used are accepted as standard for the given purposes and were the same as those used in the experiments (wherever applicable). We used parametric tests wherever sample size justified such a choice, otherwise the appropriate non-parametric tests were chosen. Tests with equal or unequal variance assumption were used depending on the characteristics of the data. All tests reported are two-tailed.

Fano factor analysis was constrained to neurons whose spike counts exceeded 0 in the analyzed stimulus conditions. Comparison of Fano factors between spontaneous and evoked activity, or across different contrast levels (**Figs. 3D-E** and **4B**) was based on mean-matched Fano factors computed following the methods in Churchland et al. (2010). Specifically, each neuron contributed one datum (point on a spike count variance vs. mean scatter plot) for each stimulus orientation, the "population" of these data points was subsampled such that the distribution of spike count means across the population was matched under the different conditions being compared, and then the Fano factor in each condition was measured as the slope of the regression line relating spike count variances to means in the subsampled population. This regression was weighted by (the inverse of) the variance of the spike count variance estimate of each datum. This procedure ensured that differences in Fano factors could not be attributed simply to changes in spike count means, or a reduction in spiking process noise due to the spike train-regularizing effect of higher mean rates, and instead reflected changes in the variability of underlying firing rates. For comparing Fano factors across orientations (**Fig. 4E**) no mean matching was used as it would have reduced the number of trials too drastically (by requiring a match of spike count mean distributions across all orientations) and because no mean matching made it easier to reject the null hypothesis that there was no difference in Fano factors (which in this case we sought to confirm). For all comparisons, the significance of the difference of Fano factors was computed based on the sampling distributions implied by the 95% confidence intervals of the regression yielding the Fano factors.

Following Vinje & Gallant (2000) and Haider et al. (2010), lifetime sparseness (**Fig. 5B**) was computed as

$$S = 1 - \frac{\left(\sum_i r_i/N\right)^2}{\sum_i r_i^2/N} \tag{S3}$$

where r_i was the firing rate of a neuron averaged across 500 trials for image frame i, and $N = 100$ was the number of frames. Sparseness analysis was constrained to neurons whose receptive fields overlapped with the smaller aperture in which the stimulus was presented (n=54). Reliability of membrane potential responses for the same set of neurons (**Fig. 5C**) was assessed by calculating the cross correlation of membrane potential sequences in each of 124,750 possible pairs of 500 trials and averaging this measure across trial pairs.

Decorrelation of population responses (**Fig. 5D**) was assessed as in Vinje & Gallant (2000). We calculated peri-stimulus time histograms (PSTHs) for neurons for 100 20-ms frames of a natural image sequence, and represented each PSTH as a vector with 100 elements. Dissimilarity of the responses of a pair of neurons was then quantified as the separation angle between their PSTH vectors in this 100-dimensional space:

$$\vartheta_{ij} = \arccos \frac{\mathbf{PSTH}_i^{\mathsf{T}}\,\mathbf{PSTH}_j}{||\mathbf{PSTH}_i||\,||\mathbf{PSTH}_j||} \tag{S4}$$

The distribution over separation angles provided a measure of signal correlations in the population response under different conditions (lower angles corresponding to higher correlations).

Noise and spontaneous correlations were calculated from z-scored spike counts in a 400-ms window. The time scale of obtaining independent samples from the posterior (20 ms in our simulations) might seem to provide a more natural time scale for computing correlations but measurement noise due to very low spike counts dominates for such short time windows and so we used longer time windows that were also compatible with those used in previously published experimental work. Trials with an absolute z-score larger than 3 were discarded from the analysis. For a cell pair to be included in the analysis, the mean firing rate (across all orientations) of both neurons needed to exceed a threshold of 0.5 Hz. As we wanted to avoid the confounding effects of low firing rates on noise correlations (de La Rocha et al., 2007), we only included trials at the stimulus orientation

for which the pair had maximal geometric mean firing rate. Signal correlations were also analyzed in 400-ms windows, excluding trials with an absolute z-score greater than 3, and cell pairs in which the mean firing rate of both cells did not exceed 0.5 Hz.

For the analysis of Kullback-Leibler (KL) divergence (**Fig. 7**), as the experimental data consisted of multiunit spike trains (Berkes et al., 2011a), we created 16 multiunit spike trains by combining the spike trains of 4 randomly selected (without replacement) model neurons for each multiunit. (Similar results were obtained by using 16 randomly selected individual model neurons for the analysis, see **Fig. S6**.) Different animals were simulated by randomizing over the assignment of model neurons to multiunits (n=20), and drawing a different random sample of membrane potentials (and hence spike counts).

KL divergence was computed between the average evoked and spontaneous activity distribution following the equation:

$$\mathrm{KL}\left[\mathrm{aEA}\,||\,\mathrm{SA}\right] = \sum_{\mathbf{n}_{0/1}} \mathrm{P}_{\mathrm{aEA}}\big(\mathbf{n}_{0/1}\big) \log_2 \frac{\mathrm{P}_{\mathrm{aEA}}\big(\mathbf{n}_{0/1}\big)}{\mathrm{P}_{\mathrm{SA}}\big(\mathbf{n}_{0/1}\big)} \qquad \text{(S5)}$$

where $\mathbf{n}_{0/1}$ is the population spike count vector of the multiunits binarized in 2 ms time bins. The average evoked activity distribution

$$\mathrm{P}_{\mathrm{aEA}}\big(\mathbf{n}_{0/1}\big) = \int \mathrm{P}\big(\mathbf{n}_{0/1}|\mathbf{x}\big)\ \mathrm{P}(\mathbf{x})\,\mathrm{d}\mathbf{x} \qquad \text{(S6)}$$

is the distribution of responses given to stimuli coming from a particular stimulus statistics, $\mathrm{P}(\mathbf{x})$. The spontaneous activity distribution $\mathrm{P}_{\mathrm{SA}}\big(\mathbf{n}_{0/1}\big)$ was based on the prior of visual features, $\mathrm{P}(\mathbf{y})$ (Eq. 3 in Experimental Procedures), or equivalently, using responses to a blank stimulus, $\mathrm{P}\big(\mathbf{n}_{0/1}|\mathbf{x}=0\big)$ (see **Fig. 2**).

In practice $\mathrm{P}_{\mathrm{aEA}}\big(\mathbf{n}_{0/1}\big)$, (Eq. S6) was computed as a histogram of responses, by sampling 600 stimuli from $\mathrm{P}(\mathbf{x})$ (i.e. randomly selected natural image patches or gratings) and for each stimulus sampling 1200 responses from the response distribution of the model, $\mathrm{P}\big(\mathbf{n}_{0/1}|\mathbf{x}\big)$ (Eqs. 5-7 in Experimental Procedures, followed by binarization), yielding a total of 720,000 sample responses. Similarly, $\mathrm{P}_{\mathrm{SA}}\big(\mathbf{n}_{0/1}\big)$ was also computed as a histogram, using the same number of sample responses. The KL divergence between the two response histograms (Eq. S5) was estimated using the methods described in Berkes et al. (2011a).

Analysis of neural variability in awake monkey recordings. In short, single unit recordings from V1 neurons were analyzed in response to static gratings with 8 orientations and two contrast levels, or moving grating images with 16 directions and a single contrast level (Ecker et al., 2010). Data were stored as spike counts in 10-ms time bins. According to the standards of the original analysis methods, only units with low contaminations (<0.05) were used in all our analyses.

For the analyses of the stimulus-dependence of neural variability (Fano factors: **Figs. 3E**, and **4B,E**), we used the static grating data set because it included several contrast levels (see above). We analyzed 100-ms segments of the neural response, preceding or following stimulus onset by 50 ms respectively for spontaneous and evoked activity. (For measuring correlations, 400-ms segments were used, see also above.) In this data set, each recording session, yielding 7-22 units, included a lower and a higher contrast condition, but the actual contrast levels in these conditions varied across sessions. To increase statistical power, we pooled data across sessions and split the data into low and high contrast conditions at a contrast level of 20% (which resulted in approximately the same number of sessions being analyzed in the two contrast conditions).

For the analysis of the orientation-dependence of Fano factors (**Fig. 4E**), the preferred orientation of units was determined by fitting Gaussian tuning curves to their orientation-dependent firing rates during stimulus presentation in a 400-ms window. The maximum of the fitted tuning curve was used as the preferred orientation of each cell and stimulus orientations were measured relative to this preferred orientation.

For the analyses involving signal correlations (**Fig. 6**) we used the moving gratings data set to compute all three types of correlations (signal, noise, and spontaneous) as this data set included more orientations and thus a more robust measure of signal correlations, and also because it yielded higher average firing rates and thus a smaller potential confounding effect of low firing rates (see also above).

All other steps of the analyses proceeded as described in the previous section to ensure uniformity of analysis for model and experimental data.

4 Change of variables: membrane potential- and firing rate-based representations

We have chosen to define the GSM in terms of abstract latent variables (representing basis function activations), $\mathbf{u}$, and then to obtain membrane potentials $\mathbf{u}$ as representing nonlinearly warped versions of $\mathbf{y}$, $u_i = f_{\mathrm{u}}(y_i)$, and firing rates $\mathbf{r}$ through a further rectified nonlinear mapping of $\mathbf{u}$, $r_i = f_{\mathrm{r}}(u_i)$ (Eqs. 6-7 in Experimental Procedures and **Fig. 1B**). The question may arise as to why we did not choose instead a generative model in which membrane potentials or instantaneous firing rates directly represent latent variables? Our choice was motivated mainly by mathematical convenience: this way, the prior distribution of our latent variables could be kept a simple multivariate normal (Eq. 3 in Experimental Procedures), while neither membrane potentials (see below) nor firing rates are normally distributed *in vivo*. Nevertheless, note that our formulation of the model is formally strictly equivalent to a model in which membrane potentials or instantaneous firing rates represent latent variables directly. In that model, the prior and the likelihood are simply the prior and the likelihood used in our model ($\mathrm{P}(\mathbf{y})$ and $\mathrm{P}(\mathbf{x}|\mathbf{y}, z)$, Eqs. 2-3 in Experimental Procedures) appropriately transformed through f_{u} (and f_{r}):

$$\mathrm{P}(\mathbf{u}) = \int \delta(\mathbf{u} - \mathbf{f}_{\mathrm{u}}(\mathbf{y}))\ \mathrm{P}(\mathbf{y})\, \mathrm{d}\mathbf{y} \tag{S7}$$

$$\mathrm{P}(\mathbf{x}|\mathbf{u}, z) = \frac{1}{\mathrm{P}(\mathbf{u})} \int \delta(\mathbf{u} - \mathbf{f}_{\mathrm{u}}(\mathbf{y}))\ \mathrm{P}(\mathbf{x}|\mathbf{y}, z)\ \mathrm{P}(\mathbf{y})\, \mathrm{d}\mathbf{y} \tag{S8}$$

or

$$\mathrm{P}(\mathbf{r}) = \int \delta(\mathbf{r} - \mathbf{f}_{\mathrm{r}}(\mathbf{f}_{\mathrm{u}}(\mathbf{y})))\ \mathrm{P}(\mathbf{y})\, \mathrm{d}\mathbf{y} \tag{S9}$$

$$\mathrm{P}(\mathbf{x}|\mathbf{r}, z) = \frac{1}{\mathrm{P}(\mathbf{r})} \int \delta(\mathbf{r} - \mathbf{f}_{\mathrm{r}}(\mathbf{f}_{\mathrm{u}}(\mathbf{y})))\ \mathrm{P}(\mathbf{x}|\mathbf{y}, z)\ \mathrm{P}(\mathbf{y})\, \mathrm{d}\mathbf{y} \tag{S10}$$

It is easy to see that both the predictive distribution over images, $\mathrm{P}(\mathbf{x})$, and the posterior distributions, $\mathrm{P}(\mathbf{u}|\mathbf{x})$ or $\mathrm{P}(\mathbf{r}|\mathbf{x})$, in that model are equivalent to those in our version. In other words, both the statistical justification (an internal model of natural image statistics) and the biological predictions (for neural responses) of our model remain the same. Including a separate membrane potential and firing rate step in our model also had the advantage that we could simultaneously test our model with both kinds of data.

Sparsely distributed membrane potential activations and the dependence of variability on orientation

One particular consequence of the non-linear mapping from $\mathbf{y}$ to $\mathbf{u}$ is a difference in their prior distributions (see also above). Specifically, the membrane potential of neurons in sensory cortices during spontaneous activity (corresponding to the prior, see the main text) has often been described as sparsely distributed (DeWeese & Zador, 2006; Okun et al., 2009). In contrast, the Gaussian scale mixture (GSM) model used in our work defines a normally distributed prior for the basis function activations which is characterized by zero sparseness. Thus, we used a weak power-law nonlinearity (**Fig. S3A**) to obtain membrane potential responses from basis function activations (Eq. 6 in Experimental Procedures) so that the membrane potential distribution under spontaneous activity became weakly kurtotic (**Fig. S3B**, excess kurtosis $\simeq 2.1$).

The same power-law nonlinearity also has another consequence: it amplifies high-amplitude membrane potential responses in both their mean and variance. This is because both the nonlinearity itself and its derivative are monotonically increasing (in absolute value) as the basis function activation is increasingly offset from zero. Therefore, this nonlinearity also predicts, in line with experimental data (Finn et al., 2007), that the variance of membrane potential responses to stimuli with preferred orientations will be proportionately slightly greater than those evoked by non-preferred stimuli (**Fig. 4C** of the main text). Higher membrane potential variance for low contrast stimulus was consistent across different levels of α and the orientation-dependence of the variance became more pronounced with higher levels of the parameter (**Fig. S3C**).

5 Relation to earlier representational models and reproducing data about average responses

Most previous representational models of V1 did not consider representing the full posterior, and instead proposed that neural activities encode a single combination of features (Olshausen & Field, 1996; Schwartz & Simoncelli, 2001; Karklin & Lewicki, 2009; Rao & Ballard, 1999). This point estimate can often be shown to correspond to the maximum probability under the posterior (*maximum a posteriori*, MAP) of a corresponding generative model (some variant of the GSM,

Richard Turner, personal communication, see also Olshausen, 1996). Thus for any input, these models assigned a single deterministic response which were taken as modelling the average response of the neuron to that stimulus as determined by its tuning curve and other receptive field properties. Indeed, these models showed prominent success at accounting for simple and complex cell receptive fields and tuning curves (Olshausen & Field, 1996; Schwartz & Simoncelli, 2001; Karklin & Lewicki, 2009).

In contrast, neural activities in our model represented uncertainty by encoding randomly sampled feature combinations under the posterior distribution. In turn, we used mean responses in our model, representing the mean of the posterior, to map receptive field properties (in close analogy to data analysis methods applied to experimental data). Nevertheless, for a reasonably unimodal and symmetric posterior distribution, the mean of the posterior will be close to its maximum. Therefore, as expected based on the formal relationship between the GSM and the generative models underlying previous work, our model replicated the success of earlier models at accounting for the classical and non-classical receptive field properties of V1 simple cells (**Fig. S2**). In particular, as the linear bases of the GSM consisted of oriented Gabor filters, neurons in our model showed clear orientation-tuning (see also section on *Key features* in the main text). Furthermore, the width of these orientation tuning curves was also contrast-invariant, and the cells also showed prominent non-classical receptive field effects, as expected based on earlier results (Schwartz & Simoncelli, 2001; Anderson et al., 2000; Finn et al., 2007), and as we explain below.

Contrast-invariant tuning

Both the membrane potential (especially at higher contrast levels) and the firing rate tuning curves of our model neurons showed contrast-invariant width (**Fig. S2A-B**). The former can be understood as a consequence of membrane potential responses in our model being a probabilistic interpretation of the model of Schwartz & Simoncelli (2001) – in which neural responses can be shown to correspond to the posterior mode (or mean, see above) of a GSM, at least approximately. Neural responses in Schwartz & Simoncelli (2001) show contrast invariant tuning curves due to divisive normalisation by a term that depends (quadratically) on a linearly filtered version of the input. Similarly, the posterior mean in our

model (Eq. 5 in Experimental Procedures) can approximately (at high contrasts) be written as $\boldsymbol{\mu} \simeq \mathbf{A}^{+}\mathbf{x}/\bar{z}$, where $\mathbf{A}^{+} = \left(\mathbf{A}\,\mathbf{A}^{\mathsf{T}}\right)^{-1}\mathbf{A}^{\mathsf{T}}$ is the (left) pseudo-inverse of $\mathbf{A}$, the bank of basis functions in our model, $\mathbf{x}$ is the input image, and $\bar{z} \propto \sqrt{\mathbf{x}\,\left(\mathbf{A}\,\mathbf{C}\,\mathbf{A}^{\mathsf{T}}\right)^{-1}\mathbf{x}^{\mathsf{T}}} \simeq \mathrm{argmax}_z\,\mathrm{P}(\mathbf{x}|z)$ is the (approximate) MAP estimate of the scaling variable, z, given the image (for the complete or overcomplete case). It is this division by $\bar{z}$, which itself depends (quadratically) on the input, that implements divisive normalization in our model, thus leading to the (approximate) contrast invariance of the posterior mean. As membrane potentials are directly related to $\mathbf{y}$ (of which $\boldsymbol{\mu}$ is the mean) through a weak nonlinearity (Eq. 6 in Experimental Procedures and **Fig. 1B**), this contrast-invariance is inherited by (average) membrane potential responses in our model (**Fig. S2A**, **C** gray line), in line with experimental data showing approximate contrast-invariance in the membrane potential responses of many V1 cells (Finn et al., 2007).

Importantly, even if membrane potential tuning curves had perfectly contrast-invariant width, this would not automatically result in contrast-invariant firing rate tuning curves, as the transformation of membrane potentials to firing rates involves rectification which could be expected to give rise to the “iceberg” effect, whereby an increasingly larger fraction of the membrane potential tuning curve exceeds the threshold of rectification as contrast is increased (Anderson et al., 2000). Thus, this “iceberg” effect would lead to a widening of the firing rate tuning curve and consequently a lack of contrast invariance (**Fig. S2C**, black line). This effect would be further exacerbated by the fact that membrane potential tuning curves themselves are already not perfectly contrast invariant and thus can show a slight increase in width with contrast (**Fig. S2C**, gray line, low contrasts).

The argument above for the iceberg effect assumed that the rectifying firing rate nonlinearity acted directly on the membrane potential tuning curves, which are defined by *trial-average* membrane potential responses. However, the firing rate nonlinearity acts on *instantaneous* membrane potentials (at a ~ 30 ms timescale, Priebe et al., 2004), which, crucially, are also affected by (noise) variability which in turn scales with contrast. This variability acts to restore the contrast invariance of firing rate tuning curves, as has been shown previously by Anderson et al. (2000) and Finn et al. (2007), whose data on membrane potential variability our model indeed reproduces (**Fig. 4** of the main text) and whose argument for contrast invariance we thus briefly recapitulate below (for more details see their papers).

Briefly, the crucial feature of contrast invariance is that suboptimally oriented stimuli at high contrast do not cause higher activity than an optimally oriented stimulus at low contrast. Crucially, in our model, membrane potential responses showed significant amounts of noise variability, and increasing stimulus contrast not only increased signal variability, but at the same time it also reduced this noise variability (**Fig. 2** of the main text, see also **Fig. S2A**, inset). This was in agreement with experimental data on the membrane potential statistics of V1 simple cells in the anesthetized cat (Finn et al., 2007).* These properties of noise variability prevented the iceberg effect in two ways. First, the additional variability smoothes out the "effective" firing rate nonlinearity, that maps from average membrane potentials to firing rates, such that its hard threshold disappears and so even stimuli for which the mean response falls below threshold can occasionally evoke spikes. Thus, simple additional variability, without contrast-dependence, will already restore contrast-invariance in firing rate tuning curves as long as membrane potential tuning curves themselves are contrast invariant (**Fig. S2C**, blue lines, high contrasts) (Anderson et al., 2000; Finn et al., 2007).

However, when membrane potential tuning curves were not contrast invariant (**Fig. S2C**, gray line, low contrasts), and in particular showed a contrast-dependent offset (**Fig. S2A**, low contrasts) this fixed amount of additional variability was insufficient to produce contrast invariance in firing rates (**Fig. S2C**, blue lines, low contrasts). In this case, the modulation of noise variability by contrast became important: the concomitant decrease in noise variance with increasing signal variance at higher contrasts meant that at suboptimal orientations a smaller fraction of the membrane potential distribution exceeded threshold, thus counteracting the iceberg effect and keeping the width of the tuning curves contrast invariant across all contrast levels (**Fig. S2B** and **C**, red line) (Finn et al., 2007). These effects were also robust to changes in the parameters of the firing rate nonlinearity within a reasonably broad range ($\pm$30-40%, including cases when the nonlinearity is sublinear rather than supralinear) (**Fig. S2D**-**E**). Therefore, despite the failure of previous attempts to reconcile sampling-based probabilistic representations with contrast invariant tuning curves (Pouget et al., 2013), our results indicate that contrast invariant tuning curves arise as a direct and robust consequence of the opposing

*Interestingly, these opposing changes in signal and noise variability with contrast were incompatible with a potentially simpler (linear) alternative account, according to which both signal and noise variability would originate from the same form of contrast-dependent variability in the input (Moreno-Bote et al., 2014). Such linear models can be extended to include normalization or adaptation, which could enable them to also exhibit mean-independent changes in noise variability.

changes in signal and noise variability of membrane potentials that are intrinsic to our model.

Non-classical receptive field effects

Nonlinear behavior of mean simple cell responses arises in the model when stimuli are presented in the nonclassical receptive field (nCRF) of the neuron (**Fig. S2F-H**). As we explain in the mean text, pixels that were not part of the "projective field" (Gabor basis function) that a neuron represented (the corresponding element of the $\mathbf{A}$ matrix was zero) could still influence the activity of this neuron due to the fundamental explaining away effect of probabilistic inference. There were two forms of explaining away in the model. First, there was explaining away via inferences in z (also explained in the main text, and illustrated in **Fig. 2** there, as well as above wrt. contrast invariance). Second, explaining away also took place via the posterior covariance matrix, $\mathbf{\Sigma}$, as the posterior mean was proportional to a product between the activities that would have resulted from a simple linear filtering of the image with the projective fields ($\mathbf{A}^\mathsf{T}\,\mathbf{x}$) and $\mathbf{\Sigma}$ (Eq. 5 in Experimental Procedures). Explaining away via z generally resulted in suppression by nCRF stimuli (**Fig. S2F-G**) simply because it increased the effective contrast-content of the image (as when aperture was increased, see corresponding section of the main text, and **Fig. 5**). This decrease could be rescued by explaining away via $\mathbf{\Sigma}$ when the nCRF stimulus was appropriately aligned with the projective fields of neurons which had positive noise correlations with the "recorded" neuron (**Fig. S2H**).

6 Establishing relationships between different forms of correlations

In order to establish a relationship for how signal, spontaneous, and noise correlations are related, we demonstrate how learning, inference, and stimulus statistics affect these quantities. First, we establish the link between signal correlations and correlations during spontaneous activity. Next, we derive a relationship between signal correlations and noise correlations. Although the derivations below regard the latent variables of the GSM, $\mathbf{y}$, we also show numerically that these results

carry over to membrane potentials, $\mathbf{u}$ (**Fig. 2** of the main text, and **Fig. S4C**), or firing rates (spike counts), $\mathbf{r}$ (**Fig. 6** of the main text).

Relationship between spontaneous and signal correlations

An important consequence of the adaptation of any well-calibrated internal model to the statistics of input stimuli can be revealed by expressing the prior distribution as the posterior distribution marginalized over the distribution of stimuli (Gelman et al., 2013; Berkes et al., 2011a):

$$\mathrm{P}(\mathbf{y}) = \int \mathrm{P}(\mathbf{y}|\mathbf{x})\ \mathrm{P}(\mathbf{x})\,\mathrm{d}\mathbf{x} \simeq \int \mathrm{P}(\mathbf{y}|\mathbf{x})\ \mathrm{P}^{*}(\mathbf{x}) \tag{S11}$$

where the average on the right is taken over the ensemble of natural images, $\mathrm{P}^{*}(\mathbf{x})$, to which the model has previously been adapted (see Section 1). As a result, a similar relationship holds for the first and second moments of responses:

$$\mathrm{E}[y_i] \simeq \mathrm{E}_{\mathbf{x}}[\mathrm{E}[y_i|\mathbf{x}]] \tag{S12}$$

$$\mathrm{E}[y_i\, y_j] \simeq \mathrm{E}_{\mathbf{x}}[\mathrm{E}[y_i\, y_j|\mathbf{x}]] \tag{S13}$$

Using the mapping of spontaneous activity to the prior distribution and stimulus-evoked activity to the posterior distribution (see main text and **Fig. 2**) and the above link between the prior distribution, the average posterior distribution, and the image statistics (Eqs. S12-S13), we can decompose the response covariance during spontaneous activity to a sum of the average noise covariance and the signal covariance:

$$\begin{aligned}
\overbrace{\mathrm{Cov}[y_i, y_j]}^{\text{cov. during spont. act.}} &= \mathrm{E}[y_i\, y_j] - \mathrm{E}[y_i]\ \mathrm{E}[y_j] \\
&= \mathrm{E}_{\mathbf{x}}[\mathrm{E}[y_i\, y_j|\mathbf{x}]] - \mathrm{E}_{\mathbf{x}}[\mathrm{E}[y_i|\mathbf{x}]]\ \mathrm{E}_{\mathbf{x}}[\mathrm{E}[y_j|\mathbf{x}]] \\
&= \mathrm{E}_{\mathbf{x}}[\mathrm{Cov}[y_i, y_j|\mathbf{x}]] + \mathrm{E}_{\mathbf{x}}[\mathrm{E}[y_i|\mathbf{x}]\ \mathrm{E}[y_j|\mathbf{x}]] - \mathrm{E}_{\mathbf{x}}[\mathrm{E}[y_i|\mathbf{x}]]\ \mathrm{E}_{\mathbf{x}}[\mathrm{E}[y_j|\mathbf{x}]] \\
&= \underbrace{\mathrm{E}_{\mathbf{x}}[\mathrm{Cov}[y_i, y_j|\mathbf{x}]]}_{\text{avg. noise cov.}} + \underbrace{\mathrm{Cov}_{\mathbf{x}}[\mathrm{E}[y_i|\mathbf{x}]\,, \mathrm{E}[y_j|\mathbf{x}]]}_{\text{signal cov.}}
\end{aligned} \tag{S14}$$

Inferring basis function activations from a high-contrast image results in a posterior with significantly smaller (co)variances than those in the prior (see **Figs. 2-3**)

and so the second term in Eq. S14 dominates, that is, spontaneous covariances are largely determined by signal covariances (**Fig. 2D**). As a consequence, correlations during spontaneous activity (which can be expressed as a weighted sum of signal and noise correlations, weighted by their respective total variances) will closely resemble signal correlations (**Fig. S4C**):

$$\underbrace{\mathrm{Corr}[y_i, y_j]}_{\text{corr. during spont. act.}} \simeq \underbrace{\mathrm{Corr}_{\mathbf{x}}[\mathrm{E}[y_i|\mathbf{x}]\,,\mathrm{E}[y_i|\mathbf{x}]]}_{\text{signal corr.}} \tag{S15}$$

Although the above argument strictly holds in the case when signal correlations are measured using natural images, it also provides a good fit when grating stimuli are used instead, even (**Fig. S4C**, green vs. blue).

Relationship between signal and noise correlations

Approximating inference over $\mathbf{y}$ being conditioned on the MAP estimate of z, $\bar{z}$ (rather than integrating out the full posterior over z, $\mathrm{P}(z|\mathbf{x})$), as we did in Section 5, we can write the posterior mean of $\mathbf{y}$ (Eq. 5 in Experimental Procedures) as:

$$\mathrm{E}[\mathbf{y}|\mathbf{x}] \simeq \boldsymbol{\mu}(\mathbf{x}, \bar{z}) = \frac{\bar{z}}{\sigma_{\mathrm{x}}^2} \boldsymbol{\Sigma}(\bar{z})\ \mathbf{A}^{\mathsf{T}}\,\mathbf{x} \tag{S16}$$

Then, relying on the fact that whitened images are used during training and testing, we assume that $\mathrm{Cov}[\mathbf{x}] \sim \mathbf{I}$. Based on these assumptions, Eq. S14 can be rewritten (again ignoring the contribution of noise covariances as above) as

$$\begin{aligned}\mathrm{Cov}[\mathbf{y}] &\approx \mathrm{E}_{\mathbf{x}}\Big[\boldsymbol{\mu}(\mathbf{x}, \bar{z})\ \boldsymbol{\mu}(\mathbf{x}, \bar{z})^{\mathsf{T}}\Big] \propto \boldsymbol{\Sigma}\,\mathbf{A}^{\mathsf{T}}\,\mathrm{E}\big[\mathbf{x}\,\mathbf{x}^{\mathsf{T}}\big]\ \mathbf{A}\,\boldsymbol{\Sigma} \\ &\propto \boldsymbol{\Sigma}\,\mathbf{A}^{\mathsf{T}}\,\mathbf{A}\,\boldsymbol{\Sigma}\end{aligned} \tag{S17}$$

At high contrast levels, the posterior (noise) covariance (Eq. 5 in Experimental Procedures) can be approximated by

$$\mathrm{Cov}[\mathbf{y}|\mathbf{x}] = \boldsymbol{\Sigma} \propto \left(\mathbf{A}^{\mathsf{T}}\,\mathbf{A}\right)^{-1} \tag{S18}$$

and thus Eq. S17, the expression for prior (spontaneous) correlations, can be simplified as

$$\mathrm{Cov}[\mathbf{y}] \propto \left(\mathbf{A}^{\mathsf{T}}\,\mathbf{A}\right)^{-1} \tag{S19}$$

This establishes the similarity of spontaneous (prior, Eq. S19) and noise (posterior, Eq. S18) correlations. Taken together with the similarity of signal and spontaneous correlations we established in the previous section (Eq. S15), this implies (by transitivity) the similarity of signal and noise correlations that can be tested both in the model and in experiments (**Fig. 6**).

Redundancy and noise correlations in overcomplete models

As we note in the Discussion of the main text, overcomplete representations, in which several latent variables represent similar (or identical) features (such that the corresponding columns of $\mathbf{A}$ are strongly non-orthogonal), may be helpful in increasing the effective number of samples in each time step. This is because in such an overcomplete system, having one sample from each of these redundant variables amounts to having several samples from a variable that represents the single shared feature. The caveat with this argument is that it relies on the assumption that the *posterior distribution* over redundant variables remains sufficiently uncorrelated – as getting many strongly correlated samples would still not be better than getting just a single sample. Yet, intuitively, one would expect explaining away to produce strong (negative) posterior (or noise) correlations between such redundant variables (eg. arising from mutual suppression between neurons representing these variables). However, this intuition really refers to the *likelihood*, so for it to carry over to the *posterior*, it in turn relies on the assumption that the *prior distribution* of redundant variables is sufficiently broad and / or uncorrelated. Crucially, the prior distribution of an overcomplete system is underconstrained by the data, so that different prior distributions will be equally good for modelling the data. (For example, for models in which the likelihood is linear, as in our GSM model, Eqs. 1-2 of the main text, the prior covariance can be anything within the appropriate null space of the feature matrix, $\mathbf{A}$). This leaves a number of degrees of freedom in the prior that could in principle be tuned to minimise the (average) correlation (or statistical dependence) of posteriors. Whether, and to what degree, such optimisation for posterior independence is possible in overcomplete systems will be an important and interesting question for future research.

7 Hierarchical inference and top-down influences on response variability in V1

The model presented in the paper aims to account for the main bottom-up determinants (i.e. signal-driven sources) of the response variability of V1 simple cells. Nevertheless, our approach can also be extended to include higher-order visual cortical areas, and in particular top-down influences on variability as statistical inference and sampling provide a self-consistent way to interpret the activities of neurons, and their interactions, at all levels of the cortical hierarchy.

Here we demonstrate that our main results on bottom-up driven changes in variability hold even in such a hierarchical model and are not simply a special case of the simple, fundamentally linear-Gaussian nature of the GSM model (at a fixed contrast level). We formulate interactions of V1 simple cell activity (or the latent variables represented thereof), $\mathbf{y}$, and higher-level (e.g. V2) neurons, $\mathbf{h}$, such that the prior over $\mathbf{y}$ depends on $\mathbf{h}$, $\mathrm{P}(\mathbf{y}|\mathbf{h})$, in potentially complex ways, rather than being a simple Gaussian. In this case, the posterior over V1-level variables given an input image $\mathbf{x}$ can be written as (c.f. Eq. 5 in Experimental Procedures):

$$\mathrm{P}(\mathbf{y}|\mathbf{x}) = \int \mathrm{P}(\mathbf{y}|\mathbf{h}, \mathbf{x})\ \mathrm{P}(\mathbf{h}|\mathbf{x})\,\mathrm{d}\mathbf{h} \tag{S20}$$

Thus, inference of $\mathbf{y}$ upon observing stimulus $\mathbf{x}$ now involves marginalization over higher-level activities. Without loss of generality, we write the expected value of $\mathbf{y}$ given $\mathbf{x}$ and $\mathbf{h}$ as a sum of two terms:

$$\mathrm{E}[\mathbf{y}|\mathbf{h}, \mathbf{x}] = \mathrm{E}[\mathbf{y}|\mathbf{h}] + \delta\overline{\mathbf{y}}(\mathbf{h}, \mathbf{x}) \tag{S21}$$

where the first term is a stimulus-independent (but $\mathbf{h}$-dependent) component and the second term summarizes the influence of the stimulus. Using the rule of total covariance we can write the response (noise) covariance as:

$$\mathrm{Cov}[\mathbf{y}|\mathbf{x}] = \mathrm{E}_{\mathbf{h}|\mathbf{x}}[\mathrm{Cov}[\mathbf{y}|\mathbf{h}, \mathbf{x}]] + \mathrm{Cov}_{\mathbf{h}|\mathbf{x}}[\mathrm{E}[\mathbf{y}|\mathbf{h}, \mathbf{x}]] \tag{S22}$$

Given these preliminaries, we now consider the case of a stimulus (e.g. a grating) that does not elicit a concrete higher order percept, such that the posterior over higher-order variables, $\mathbf{h}$, remains similar to the prior:

$$\mathrm{P}(\mathbf{h}|\mathbf{x}) \simeq \mathrm{P}(\mathbf{h}) \tag{S23}$$

This allows us to rewrite the posterior covariance of $\mathbf{y}$ (Eq. S22) as

$$\mathrm{Cov}[\mathbf{y}|\mathbf{x}] \simeq \mathrm{E}_{\mathbf{h}}[\mathrm{Cov}[\mathbf{y}|\mathbf{h},\mathbf{x}]] + \mathrm{Cov}_{\mathbf{h}}[\mathrm{E}[\mathbf{y}|\mathbf{h},\mathbf{x}]] \tag{S24}$$

In general, we expect the sum in Eq. S24 to be dominated by the second term as the amount of variation in (average) V1 activations due to changes in $\mathbf{h}$, $\mathrm{Cov}_{\mathbf{h}}[\mathrm{E}[\mathbf{y}|\mathbf{h},\mathbf{x}]]$, is expected to be much larger than the residual (noise) covariance when both $\mathbf{h}$ and $\mathbf{x}$ are fixed, $\mathrm{E}_{\mathbf{h}}[\mathrm{Cov}[\mathbf{y}|\mathbf{h},\mathbf{x}]]$. This will certainly hold when higher order percepts can elicit reliable responses even in the absence of the actual low-level visual feature that defines the classical receptive field and tuning curve of a cell, as is the case for V1 neurons that respond to illusory contours (Grosof et al., 1993; Lee & Nguyen, 2001) and perceived brightness (Rossi et al., 1996). Thus we can write that

$$\mathrm{Cov}[\mathbf{y}|\mathbf{x}] \mathrel{\tilde{\propto}} \mathrm{Cov}_{\mathbf{h}}[\mathrm{E}[\mathbf{y}|\mathbf{h},\mathbf{x}]] \tag{S25}$$

Next, applying Eq. S21 to S25 yields

$$\begin{aligned}\mathrm{Cov}[\mathbf{y}|\mathbf{x}] &\mathrel{\tilde{\propto}} \mathrm{Cov}_{\mathbf{h}}[\mathrm{E}[\mathbf{y}|\mathbf{h}]] + \mathrm{Cov}_{\mathbf{h}}[\delta\overline{\mathbf{y}}(\mathbf{h},\mathbf{x})] + \\ &\quad + 2\,\mathrm{Cov}_{\mathbf{h}}[\mathrm{E}[\mathbf{y}|\mathbf{h}]\,, \delta\overline{\mathbf{y}}(\mathbf{h},\mathbf{x})] \qquad \text{(S26)} \\ &\mathrel{\tilde{\propto}} \mathrm{Cov}_{\mathbf{h}}[\mathrm{E}[\mathbf{y}|\mathbf{h}]] \qquad \text{(S27)}\end{aligned}$$

where we made the additional but reasonable assumption that $\mathbf{x}$ elicits consistent changes in (the average) $\mathbf{y}$ across the range of possible settings of $\mathbf{h}$, and so the corresponding covariances (the last two terms in Eq. S26) can be neglected.

We also note that the prior (ie. spontaneous) covariance (of V1-level variables) in this model can be written simply as

$$\mathrm{Cov}[\mathbf{y}] = \mathrm{E}_{\mathbf{h}}[\mathrm{Cov}[\mathbf{y}|\mathbf{h}]] + \mathrm{Cov}_{\mathbf{h}}[\mathrm{E}[\mathbf{y}|\mathbf{h}]] \tag{S28}$$

Again, under the same conditions as above (higher order percepts can elicit reliable responses even in the absence of the appropriate low-level visual feature), we expect this sum to be dominated by the second term, and so we can write

$$\mathrm{Cov}[\mathbf{y}] \mathrel{\tilde{\propto}} \mathrm{Cov}_{\mathbf{h}}[\mathrm{E}[\mathbf{y}|\mathbf{h}]] \tag{S29}$$

Thus, we can conclude from Eq. S27 that when the stimulus carries limited information about higher-level inferences, such as in the case of simplistic artificial

stimuli, the noise correlation structure is largely independent of the stimulus.[†] Comparing Eq. S27 and S29 also shows that, just as in the GSM, in this case prior (spontaneous) and posterior (noise) correlations are also expected to be similar. In addition, note that the arguments for why spontaneous activity distribution (and thus correlations) will match average evoked activity distributions (and thus correlations), and that spontaneous correlations will be dominated by noise correlations (Section 6) are general and do not make use of the specifics of the GSM. This also means that signal and noise correlations will also be similar in this hierarchical model for non-naturalistic stimuli.

For more naturalistic stimuli, the stimulus will afford higher level inferences and constrain the posterior over $\mathbf{h}$ such that its posterior is no longer equivalent to its prior, $\mathrm{P}(\mathbf{h}|\mathbf{x}) \neq \mathrm{P}(\mathbf{h})$ and instead becomes highly constrained. In this case, the two terms in Eq. S24 are averages and covariances over $\mathrm{P}(\mathbf{h}|\mathbf{x})$, not $\mathrm{P}(\mathbf{h})$, and in particular the covariance over $\mathrm{P}(\mathbf{h}|\mathbf{x})$ becomes much smaller. This predicts that noise (co)variability in V1 responses ($\mathbf{y}$) will be reduced for natural stimuli, which agrees with the finding that natural images evoke more reliable and sparser responses than their phase-scrambled counterparts (which do not afford higher-order percepts) (Froudarakis et al., 2014). As a special case, this also implies a hierarchical version of the prediction, tested in the main text (**Figs. 3-5**), that increasing contrast or stimulus aperture (presumably better allowing higher-order percepts to arise) leads to decreased variability and increased reliability, sparseness, and decorrelation, and that these effects should be particularly prominent when using natural stimuli (Haider et al., 2010; Vinje & Gallant, 2000).

Table S2 summarises these results on how hierarchical inference accounts for all the main effects obtained by using a GSM elsewhere in the paper.

[†]As a sanity check, note that using the same assumptions, the response mean is – quite appropriately – *not* independent of the stimulus:

$$\begin{aligned} \mathrm{E}[\mathbf{y}|\mathbf{x}] &= \mathrm{E}_{\mathbf{h}|\mathbf{x}}[\mathrm{E}[\mathbf{y}|\mathbf{h},\mathbf{x}]] \simeq \mathrm{E}_{\mathbf{h}}[\mathrm{E}[\mathbf{y}|\mathbf{h},\mathbf{x}]] \\ &= \mathrm{E}_{\mathbf{h}}[\mathrm{E}[\mathbf{y}|\mathbf{h}]] + \mathrm{E}_{\mathbf{h}}[\delta\overline{\mathbf{y}}(\mathbf{h},\mathbf{x})] = \mathrm{E}[\mathbf{y}] + \mathrm{E}_{\mathbf{h}}[\delta\overline{\mathbf{y}}(\mathbf{h},\mathbf{x})] \end{aligned} \tag{S30}$$

and there is no reason to assume that the expectation (rather than the variance) of $\delta\overline{\mathbf{y}}(\mathbf{h},\mathbf{x})$ is small (relative to the prior mean of $\mathbf{y}$, $\mathrm{E}[\mathbf{y}]$).

Nonlinear relationship between low-level and high-level confidence

While our model only predicted (in line with experimental data) a relatively modest (less than a factor of 2) reduction in (membrane potential) variance at the level of V1 at the onset of a high contrast stimulus (**Fig. 3**), perceptual confidence typically undergoes a drastic, orders-of-magnitude change under the same condition. This seeming contradiction can be reconciled by considering nonlinear effects that are ubiquitous in inference under hierarchical models (in which perceptual confidence corresponds to inferences over higher-level variables). The key insight is that the variance of the posterior over a higher-level variable will in general depend not only on the variance of a low level posterior but also on its mean.

As a toy example, consider a generative model[‡] with a single, binary higher level variable $h = \pm 1$ (related to eg. whether one or another stimulus is present in a two-alternative forced choice perceptual decision making task) which is related to V1-level variables through a simple (multivariate) normal likelihood, such as:

$$\mathrm{P}(h) = \mathrm{Bernoulli}\left(\frac{h+1}{2}; \frac{1}{2}\right) \tag{S31}$$

$$\mathrm{P}(\mathbf{y}|h) = \mathcal{N}\left(\mathbf{y}; \frac{\mathbf{a}\,h}{2}, \sigma_{\mathrm{y}}^2\,\mathbf{I}\right) \tag{S32}$$

In this model, the conditional probability of $h = +1$ for a given $\mathbf{y}$ can be written as

$$\mathrm{P}(h = +1|\mathbf{y}) = \mathrm{Sigmoid}(f)\,, \qquad \text{with } f = \frac{\mathbf{a}^{\mathsf{T}}\,\mathbf{y}}{\sigma_{\mathrm{y}}^2} \tag{S33}$$

where $\mathrm{Sigmoid}(f) = 1/\left(1 + e^{-f}\right)$ is the standard logistic sigmoid function (**Fig. S3E**, middle, red curve). Finally, the posterior over h given an input image $\mathbf{x}$ can be written by marginalising out the posterior over $\mathbf{y}$:

$$\mathrm{P}(h = +1|\mathbf{x}) = \int \mathrm{P}(h = +1|\mathbf{y})\ \mathrm{P}(\mathbf{y}|\mathbf{x})\,\mathrm{d}\mathbf{y} \tag{S34}$$

Note that this marginalisation really only depends on the relevant projection of $\mathbf{y}$, that is f, and the projected posterior will usually be approximately normal

[‡]This model is a simplified special case of the model we consider for decoding a multinomial orientation variable from V1 responses in Section 8.

(due to the central limit theorem, if $\mathbf{y}$ is sufficiently high dimensional – and for the GSM also because the individual elements of $\mathbf{y}$ are already approximately normally distributed even under the posterior – **Fig. S3E**, middle, blue and brown Gaussians). Thus, Eq. S34 can be rewritten as

$$\mathrm{P}(h = +1|\mathbf{x}) \simeq \int \mathrm{Sigmoid}(f)\ \mathcal{N}\big(f; \mu_\mathrm{f}, \sigma_\mathrm{f}^2\big)\ \mathrm{d}f \tag{S35}$$

$$\text{where } \mu_\mathrm{f} = \mathbf{a}^\mathsf{T}\ \boldsymbol{\mu}_\mathrm{y}/\sigma_\mathrm{y}^2, \text{ and } \sigma_\mathrm{f}^2 = \mathbf{a}^\mathsf{T}\ \boldsymbol{\Sigma}_\mathrm{y}\ \mathbf{a}/\sigma_\mathrm{y}^4 \tag{S36}$$

and $\boldsymbol{\mu}_\mathrm{y}$ and $\boldsymbol{\Sigma}_\mathrm{y}$ are the posterior mean and covariance of $\mathbf{y}$. In general (as in our non-hierarchical GSM model), there are at least two aspects of the $\mathbf{y}$-posterior (and its f-projection) that change when contrast goes to zero: its (co)variance grows, and its mean goes towards zero (see main text and **Fig. 2**). Crucially, the h-posterior, $\mathrm{P}(h = +1|\mathbf{x})$, and thus its variance depends on *both* these statistics, not only the change in the variance of $\mathbf{y}$ (or f). Our goal is to understand how changes in the variance of low-level variables, α_y^2, relate to changes in the variance of the high-level variable, α_h^2, in this model.

For an illustration, we consider two cases, before stimulus onset, and after the onset of a stimulus which is such that the posterior mean of $\mathbf{y}$ is just as expected for the $+1$ stimulus, i.e. $\boldsymbol{\mu}_\mathrm{y} = \mathbf{a}$ (**Fig. S3E**, bottom, blue vs. brown distributions). Thus, the f-posterior will have a mean $\mu_\mathrm{f} = 0$ and $\mu_\mathrm{f} = \mu_\mathrm{f}^+ = \mathbf{a}^\mathsf{T}\,\mathbf{a}/\sigma_\mathrm{y}^2$ and variance $\sigma_\mathrm{f}^2 = \sigma_\mathrm{f0}^2$ and $\sigma_\mathrm{f}^2 = \sigma_\mathrm{f0}^2/\alpha_\mathrm{y}^2$, before and after stimulus onset, respectively, where $\alpha_\mathrm{y}^2 > 1$ is the relative reduction of $\mathbf{y}$-variance at stimulus onset (**Fig. S3E**, middle, blue vs. brown Gaussians). Note that as f is a linear projection of $\mathbf{y}$, the variance of its (normally distributed) posterior will also be reduced by the same factor, α_y^2. As before stimulus onset, the mean of f is sitting at exactly $\mu_\mathrm{f} = 0$, which is the classification boundary for h in f-space (**Fig. S3E**, straight red line), and its distribution is symmetric around it (being approximately normal), its probability mass on the two sides of the boundary will be equal, and so the posterior distribution of h is uniform. Thus, before stimulus onset, the posterior variance of h is maximal, i.e. it is equal to 1 (**Fig. S3E**, top, blue). After stimulus onset, the h-posterior is a Bernoulli variable with probability (**Fig. S3E**, top, brown):

$$\mathrm{P}(h = +1|\mathbf{y}) = \int \mathrm{Sigmoid}(f)\ \mathcal{N}\big(f; \mu_\mathrm{f}^+, \sigma_\mathrm{f0}^2/\alpha_\mathrm{y}^2\big)\ \mathrm{d}f \tag{S37}$$

from which the relative reduction in the variance of h can be obtained as

$$\alpha_\mathrm{h}^2 = \frac{1}{4\,\mathrm{P}(h = +1|\mathbf{y})\ [1 - \mathrm{P}(h = +1|\mathbf{y})]} \tag{S38}$$

In sum, Eqs. S37-S38 establish the dependence of α_h^2 on α_y^2 and identify the relevant parameters of this dependence as μ_f^+ ('signal') and σ_f0^2 ('noise', **Fig. S3F**). As we can see, there is a nonlinear interplay between the effect of the mean and variance of the $\mathbf{y}$-posterior on inferences about h, such that especially for high signal and low noise, the reduction in h-variance, α_h^2, can be orders of magnitude greater than the reduction in $\mathbf{y}$-variance, α_y^2. In particular, note that even when there is no reduction in $\mathbf{y}$-variance ($\alpha_\mathrm{y}^2 = 1$), there can still be substantial reduction in h-variance ($\alpha_\mathrm{h}^2 \gg 1$).

Finally, we expect (as long as the $\mathbf{y}$ posterior only has finite-range correlations, as in our GSM model) both μ_f^+ and σ_f0^2 to scale linearly with N (the number of V1-level variables on which the perceptual decision depends). Thus, in the limit of large N, the posterior over f goes to a delta distribution which simplifies Eq. S37 to

$$\mathrm{P}(h = +1|\mathbf{x}) \simeq \mathrm{Sigmoid}\big(\mu_\mathrm{f}^+\big) \tag{S39}$$

and so plugging Eq. S39 into Eq. S38 reveals that $\alpha_\mathrm{h}^2 \propto e^N$. In other words, the reduction in h-variance can be arbitrarily large (with sufficiently high N) irrespective of the value of α_y^2.

8 The relevance of stimulus-dependent changes in neural variability: decoding stimuli from spike trains

Our sampling-based model encodes the posterior distribution over visual features such that Fano factors and noise correlations of V1 responses depend on particular attributes of the stimulus (Figs. 4-6). This is in contrast with other probabilistic encoding schemes that do not predict such stimulus-dependent modulations, and in fact specifically assume their absence (Ma et al., 2006). Although experimental data do show the modulations that our theory predicts (Churchland et al., 2010; Ecker et al., 2010; Finn et al., 2007; Haider et al., 2010; Carandini, 2004; Berkes et al., 2011a) (**Figs. 4-6**), it is difficult to assert whether these are actually relevant for neural computations or they can be regarded epiphenomenal. One prominent approach to make inroads in this issue employs a decoding framework: it is based on measuring how well the stimulus can be inferred from (simulated or experimentally measured) neural responses using different decoder algorithms (Averbeck et al., 2006; Pillow et al., 2008). The relevance of a particular aspect of

the neural code, such as the stimulus-dependence of (co)variability in our model, can then be assessed by comparing a decoder which takes that aspect into account with one that ignores it: if the two decoders perform similarly then the given aspect of the neural code can be deemed irrelevant, if they do not then it is likely functionally relevant.

To assess the functional relevance of stimulus-dependent changes in variability in our model, we compared the performance of two algorithms decoding the stimulus from spike counts generated by the model (**Fig. S5**). The first was a linear decoder, which ignored these variability modulations (Berens et al., 2012; Ma, 2010) but was otherwise optimised for inferring the stimulus. The second was an optimal decoder, which took the variability modulations into account in a formally optimal way by explicitly inverting the probabilistic process that led from stimulus to spike counts in the model (**Fig. 1B**, Experimental Procedures, see also the end of this section for the mathematical definition of the two decoders). Spike counts were measured in 20 ms windows from 42 randomly chosen neurons in response to static gratings that varied in four attributes: orientation (12 different values), phase (12), contrast levels (7), and aperture size (7). In a given decoding task, two of these attributes remained fixed and known to the decoders, while the other two were unknown and varied from trial to trial. Of the two unknown attributes, one needed to be decoded while the other disregarded (which thus acted as a nuisance parameter that needed to be marginalised out). Performance of the decoders was assessed by the fraction of trials on which they assigned the highest probability to the correct value of the stimulus attribute that needed to be decoded.

When orientation had to be decoded at unknown contrasts, the linear and optimal decoders performed similarly (**Fig. S5A**). However, when orientation had to be decoded at known contrast levels but at unknown phases, or when stimulus phase had to be decoded with orientation unknown, the optimal decoder substantially outperformed the linear decoder at high contrast levels (**Fig. S5B-C**). There was a similar difference between the two decoders at high contrast levels for orientation decoding with unknown aperture sizes (**Fig. S5D**). Training individual linear decoders for distinct (discretized) values of the nuisance parameters abolished the advantage of the optimal decoder (data not shown), indicating that knowledge of nuisance parameters would ameliorate their effect on a linear decoder.

These results indicate that our sampling-based population code cannot be generally decoded, and thus interpreted, as a linear invariant PPC (Ma et al., 2006).

While it may still be possible to decode our network as a more general linear (non-invariant) PPC, there is currently no theory for how such a PPC could be instantiated in cortical circuits[§]. In sum, the variability modulations in our model that we tested against experimental data (**Figs. 4-6**) are functionally relevant in a variety of scenarios, making sampling-based representations fundamentally distinct from other probabilistic encoding schemes, such as probabilistic population codes for which linear decoding is optimal (Ma et al., 2006).

Although previous decoding-based analyses of experimental data suggested that linear decoders might be sufficient to achieve optimal decoding performance (Graf et al., 2011; Berens et al., 2012), they only considered simple decoding tasks in which one particular attribute, orientation, had to be decoded either without any nuisance parameters, or only contrast as a nuisance parameter – in which case we also found linear decoding to be near-optimal (**Fig. S5A**). (Note that using shifting rather than static gratings of stimuli, as was done in Graf et al., 2011, is not equivalent to requiring the decoder to marginalise over phase.) However, our results suggest that a sound experimental test of the importance of response variability modulations should include more diverse and challenging decoding tasks employing a richer set of possible combinations for which stimulus attributes need to be decoded and which others marginalised. We predict that once such tasks are considered, linear decoding of population responses will significantly fall short of being optimal.

Definition of decoders

The linear decoder assessed the probability of a stimulus attribute, θ, given a vector of spike counts in the population, $\mathbf{n}$, as the following:

$$\mathrm{P}(\theta|\mathbf{n}) = \frac{\exp\left(\mathbf{w}_\theta^\mathsf{T}\mathbf{n} + \eta_\theta\right)}{\exp\left(\sum_{\theta'}\mathbf{w}_{\theta'}^\mathsf{T}\mathbf{n} + \eta_{\theta'}\right)} \tag{S40}$$

where $\mathbf{w}_\theta$ and η_θ are a vector of decoder weights and a scalar bias associated to the particular value of the stimulus attribute, and the denominator provides a normalization of the probability. The decoder was trained (its parameters, $\mathbf{w}_\theta$ and η_θ, optimized) on a set of stimulus-response pairs corresponding to the specific

[§]Although it might be possible to extend the approach of Beck et al. (2011) to this particular problem.

decoding task. The training data consisted of 2400 repetitions of each value of the decoded stimulus attribute while the nuisance parameter was varied randomly and uniformly and the other parameters were kept fixed. (Note that this included multiple presentations of each stimulus which was necessary because model responses were stochastic.) Testing was performed in cross validation, i.e. using a data set of responses that were separate from that used for training.

The optimal decoder used the generative model to compute the probability of a particular stimulus attribute given the spike counts of model neurons. The generative model defined the posterior distribution over visual feature activations, $\mathbf{y}$ given the stimulus $\mathbf{x}$ (Eqs. 2-5 in Experimental Procedures), and feature activations were encoded deterministically in membrane potentials and, in turn, firing rates $\mathbf{r}$ (Eqs. 6-7 in Experimental Procedures). As a decoding window of $T = 20\,\mathrm{ms}$ was used in our simulations, the spike counts recorded, $\mathbf{n}$, represented a single sample from the posterior, and thus the conditional probability of observing spike counts $\mathbf{n}$ could be formalised using a multivariate Bernoulli distribution (over $\lfloor r_i T \rfloor$ and $\lfloor r_i T \rfloor + 1$):

$$\mathrm{P}(\mathbf{n}|\mathbf{r}) = \prod_i \mathrm{P}(n_i|r_i) \tag{S41}$$

$$\mathrm{P}(n_i|r_i) = \begin{cases} \mathrm{Bernoulli}(n_i - \lfloor r_i T \rfloor; r_i - \lfloor r_i T \rfloor) & n_i - \lfloor r_i T \rfloor \in \{0, 1\} \\ 0 & \text{otherwise} \end{cases} \tag{S42}$$

As only $\mathbf{x}$ and $\mathbf{n}$ but not $\mathbf{y}$ (or z) were directly known to the decoder, $\mathbf{y}$ (and z) needed to be marginalized out to obtain the likelihood of a particular stimulus:

$$\mathrm{P}(\mathbf{n}|\mathbf{x}) = \int \mathrm{P}(\mathbf{n}|\mathbf{r}(\mathbf{y}))\ \mathrm{P}(\mathbf{y}|\mathbf{x}, z)\ \mathrm{P}(z|\mathbf{x})\ d\mathbf{y}\, dz \tag{S43}$$

Computing the multi-dimensional integral in Eq. S43 is intractable, therefore we performed a Monte Carlo integral to approximate the true value by obtaining samples from $\mathrm{P}(z|\mathbf{x})$ and $\mathrm{P}(\mathbf{y}|\mathbf{x}, z)$. Finally, the particular stimulus attribute of interest can be decoded using the (uniform) prior over stimulus attributes, $\mathrm{P}(\theta)$, and the distribution $\mathrm{P}(\mathbf{x}|\theta)$, which is uniform over the discrete and finite set of stimuli which are obtained by keeping this attribute fixed while varying the nuisance attribute of the stimulus:

$$\mathrm{P}(\theta|\mathbf{n}) \propto \mathrm{P}(\theta) \sum_{\mathbf{x}} \mathrm{P}(\mathbf{n}|\mathbf{x})\ \mathrm{P}(\mathbf{x}|\theta) \tag{S44}$$

As only a small number of stimuli were compatible with any given value of θ, the sum in Eq. S44 was evaluated by simple enumeration. Note that the optimal decoder did not have free parameters and thus it did not need a separate training phase.

References

Anderson, J. S., Lampl, I., Gillespie, D. C., & Ferster, D. (2000). The contribution of noise to contrast invariance of orientation tuning in cat visual cortex. *Science*, *290*(5498), 1968–1972.

Averbeck, B. B., Latham, P. E., & Pouget, A. (2006). Neural correlations, population coding and computation. *Nature Reviews Neuroscience*, *7*(5), 358–366.

Azouz, R., & Gray, C. M. (1999). Cellular mechanisms contributing to response variability of cortical neurons in vivo. *Journal of Neuroscience*, *19*(6), 2209–2223.

Beck, J. M., Latham, P. E., & Pouget, A. (2011). Marginalization in Neural Circuits with Divisive Normalization. *Journal of Neuroscience*, *31*, 15310–15319.

Berens, P., Ecker, A. S., Cotton, R. J., Ma, W. J., Bethge, M., & Tolias, A. S. (2012). A fast and simple population code for orientation in primate V1. *Journal of Neuroscience*, *32*(31), 10618–10626.

Berkes, P., Orbán, G., Lengyel, M., & Fiser, J. (2011a). Spontaneous cortical activity reveals hallmarks of an optimal internal model of the environment. *Science*, *331*(6013), 83–87.

Berkes, P., Turner, R. E., & Fiser, J. (2011b). The army of one (sample): the characteristics of sampling-based probabilistic neural representations. In *Frontiers in Neuroscience*.

Büsing, L., Bill, J., Nessler, B., & Maass, W. (2011). Neural dynamics as sampling: a model for stochastic computation in recurrent networks of spiking neurons. *PLoS Computational Biology*, *7*(11), e1002211.

Carandini, M. (2004). Amplification of trial-to-trial response variability by neurons in visual cortex. *PLoS Biology*, *2*(9), E264.

Churchland, M. M., Yu, B. M., Cunningham, J. P., Sugrue, L. P., Cohen, M. R., Corrado, G. S., Newsome, W. T., Clark, A. M., Hosseini, P., Scott, B. B., Bradley, D. C., Smith, M. A., Kohn, A., Movshon, J. A., Armstrong, K. M., Moore, T., Chang, S. W., Snyder, L. H., Lisberger, S. G., Priebe, N. J., Finn, I. M., Ferster, D., Ryu, S. I., Santhanam, G., Sahani, M., & Shenoy, K. V. (2010). Stimulus onset quenches neural variability: a widespread cortical phenomenon. *Nature Neuroscience*, *13*(3), 369–378.

de La Rocha, J., Doiron, B., Shea-Brown, E., Josić, K., & Reyes, A. (2007). Correlation between neural spike trains increases with firing rate. *Nature*, *448*(7155), 802–806.

Dempster, A. P., Laird, N. M., & Rubin, D. B. (1977). Maximum likelihood from incomplete data via the EM algorithm. *Journal of the Royal Statistical Society. Series B (Methodological)*, *39*, 1–38.

DeWeese, M. R., & Zador, A. M. (2006). Non-Gaussian membrane potential dynamics imply sparse, synchronous activity in auditory cortex. *Journal of Neuroscience*, *26*(47), 12206–12218.

Ecker, A. S., Berens, P., Cotton, R. J., Subramaniyan, M., Denfield, G. H., Cadwell, C. R., Smirnakis, S. M., Bethge, M., & Tolias, A. S. (2014). State dependence of noise correlations in macaque primary visual cortex. *Neuron*, *82*(1), 235–248.

Ecker, A. S., Berens, P., Keliris, G. A., Bethge, M., Logothetis, N. K., & Tolias, A. S. (2010). Decorrelated neuronal firing in cortical microcircuits. *Science*, *327*(5965), 584–587.

Finn, I. M., Priebe, N. J., & Ferster, D. (2007). The emergence of contrast-invariant orientation tuning in simple cells of cat visual cortex. *Neuron*, *54*(1), 137–152.

Froudarakis, E., Berens, P., Ecker, A. S., Cotton, R. J., Sinz, F. H., Yatsenko, D., Saggau, P., Bethge, M., & Tolias, A. S. (2014). Population code in mouse V1 facilitates readout of natural scenes through increased sparseness. *Nature Neuroscience*, *17*(6), 851–857.

Gelman, A., Carlin, J. B., Stern, H. S., Dunson, D. B., Vehtari, A., & Rubin, D. B. (2013). *Bayesian Data Analysis*. CRC Press, 3rd ed.

Goris, R. L. T., Movshon, J. A., & Simoncelli, E. P. (2014). Partitioning neuronal variability. *Nature Neuroscience*, *17*(6), 858–865.

Grabska-Barwinska, A., Beck, J., Pouget, A., & Latham, P. (2013). Demixing odors - fast inference in olfaction. In C. J. C. Burges, L. Bottou, M. Welling, Z. Ghahramani, & K. Q. Weinberger (Eds.) *Advances in Neural Information Processing Systems 26*, (pp. 1968–1976). Curran Associates, Inc.

Graf, A. B. A., Kohn, A., Jazayeri, M., & Movshon, J. A. (2011). Decoding the activity of neuronal populations in macaque primary visual cortex. *Nature Neuroscience*, *14*(2), 239–245.

Grosof, D. H., Shapley, R. M., & Hawken, M. J. (1993). Macaque V1 neurons can signal 'illusory' contours. *Nature*, *365*(6446), 550–552.

Haider, B., Häusser, M., & Carandini, M. (2013). Inhibition dominates sensory responses in the awake cortex. *Nature*, *493*(7430).

Haider, B., Krause, M. R., Duque, A., Yu, Y., Touryan, J., Mazer, J. A., & McCormick, D. A. (2010). Synaptic and network mechanisms of sparse and reliable visual cortical activity during nonclassical receptive field stimulation. *Neuron*, *65*(1), 107–121.

Hennequin, G., Aitchison, L., & Lengyel, M. (2014). Fast Sampling-Based Inference in Balanced Neuronal Networks. In Z. Ghahramani, M. Welling, C. Cortes, N. D. Lawrence, & K. Q. Weinberger (Eds.) *Advances in Neural Information Processing Systems 27*, (pp. 2240–2248). Curran Associates, Inc.

Karklin, Y., & Lewicki, M. S. (2009). Emergence of complex cell properties by learning to generalize in natural scenes. *Nature*, *457*(7225), 83–86.

Kohn, A., & Smith, M. A. (2005). Stimulus dependence of neuronal correlation in primary visual cortex of the macaque. *Journal of Neuroscience*, *25*(14), 3661–3673.

Lee, T. S., & Nguyen, M. (2001). Dynamics of subjective contour formation in the early visual cortex. *Proceedings of the National Academy of Sciences of the United States of America*, *98*(4), 1907–1911.

Ma, W. J. (2010). Signal detection theory, uncertainty, and Poisson-like population codes. *Vision Research*, *50*(22), 2308–2319.

Ma, W. J., Beck, J. M., Latham, P. E., & Pouget, A. (2006). Bayesian inference with probabilistic population codes. *Nature Neuroscience*, *9*(11), 1432–1438.

Mante, V., Frazor, R. A., Bonin, V., Geisler, W. S., & Carandini, M. (2005). Independence of luminance and contrast in natural scenes and in the early visual system. *Nature Neuroscience*, *8*(12), 1690–1697.

Moreno-Bote, R., Beck, J., Kanitscheider, I., Pitkow, X., Latham, P., & Pouget, A. (2014). Information-limiting correlations. *Nature Neuroscience*, *17*(10), 1410–1417.

Okun, M., Mokeichev, A., Katz, Y., & Lampl, I. (2009). On the dynamics of synaptic inputs during ongoing activity in the cortex. In K. Josić, M. A. Matias, J. Rubin, & R. Romo (Eds.) *Coherent Behavior in Neuronal Networks*, (pp. 1–16). Springer.

Olshausen, B. A. (1996). Learning linear, sparse, factorial codes. Tech. Rep. AIM-1580, Artificial Intelligence Laboratory, Massachusetts Institute of Technology.

Olshausen, B. A., & Field, D. J. (1996). Emergence of simple-cell receptive field properties by learning a sparse code for natural images. *Nature*, *381*(6583), 607–609.

Pillow, J. W., Shlens, J., Paninski, L., Sher, A., Litke, A. M., Chichilnisky, E. J., & Simoncelli, E. P. (2008). Spatio-temporal correlations and visual signalling in a complete neuronal population. *Nature*, *454*(7207), 995–999.

Portilla, J., & Simoncelli, E. P. (2000). A parametric texture model based on joint statistics of complex wavelet coefficients. *International Journal of Computer Vision*, *40*(1), 49–70.

Pouget, A., Beck, J. M., Ma, W. J., & Latham, P. E. (2013). Probabilistic brains: knowns and unknowns. *Nature Neuroscience*, *16*(9), 1170–1178.

Priebe, N. J., Mechler, F., Carandini, M., & Ferster, D. (2004). The contribution of spike threshold to the dichotomy of cortical simple and complex cells. *Nature Neuroscience*, *7*(10), 1113–1122.

Rao, R. P. N., & Ballard, D. H. (1999). Predictive coding in the visual cortex: a functional interpretation of some extra-classical receptive-field effects. *Nature Neuroscience*, *2*(1), 79–87.

Rossi, A. F., Rittenhouse, C. D., & Paradiso, M. A. (1996). The representation of brightness in primary visual cortex. *Science*, *273*(5278), 1104–1107.

Savin, C., Dayan, P., & Lengyel, M. (2014). Optimal recall from bounded metaplastic synapses: predicting functional adaptations in hippocampal area CA3. *PLoS Computational Biology*, *10*(2), e1003489.

Schwartz, O., Sejnowski, T. J., & Dayan, P. (2009). Perceptual organization in the tilt illusion. *Journal of Vision*, *9*(4), 19.1–20.

Schwartz, O., & Simoncelli, E. P. (2001). Natural signal statistics and sensory gain control. *Nature Neuroscience*, *4*(8), 819–825.

van Hateren, J. H. (1992). Real and optimal neural images in early vision. *Nature*, *360*(6399), 68–70.

Vinje, W. E., & Gallant, J. L. (2000). Sparse coding and decorrelation in primary visual cortex during natural vision. *Science*, *287*(5456), 1273–1276.

Wainwright, M. J., & Simoncelli, E. P. (2000). Scale mixtures of Gaussians and the statistics of natural images. In S. A. Solla, T. K. Leen, & K.-R. Muller (Eds.) *Advances in Neural Information Processing Systems 12*, (pp. 855–861). MIT Press, Cambridge, MA.

figure	reference	species	condition	recording
3A-C	Finn et al. (2007) (as analysed in Churchland et al., 2010)	cat	anesthesia	intracellular single cell
3D	Kohn & Smith (2005) (as analysed in Churchland et al., 2010)	macaque	anesthesia	extracellular multi-electrode single unit
4A,C	Finn et al. (2007)	cat	anesthesia	intracellular single cell
3E, 4B,D,E, 6	Ecker et al. (2010)	macaque	awake	extracellular multi-electrode single unit
5A,B,C	Haider et al. (2010)	cat	anesthesia	intracellular single cell
5D	Vinje & Gallant (2000)	macaque	awake	extracellular multi-electrode multiunit
7	Berkes et al. (2011a)	ferret	awake	extracellular multi-electrode multiunit

Table S1. Related to Figs. 3–7. Summary of experimental data to which model results and predictions were compared. See data selection criteria in Section 2.

result	GSM	hierarchical model
stimulus onset quenches variability	✓(Fig. 3)	✓
contrast decreases variability	✓(Fig. 4)	✓
aperture increases reliability, sparseness, and decorrelation	✓(Fig. 5)	✓ esp. for natural stimuli
natural stimuli increases reliability, sparseness, and decorrelation	×	✓
noise correlations do not depend on the stimulus	✓(Fig. 2)	for non-natural stimuli
spontaneous correlations are similar to signal correlations	✓(Fig. 6)	✓
noise correlations are similar to signal correlations	✓(Fig. 6)	for non-natural stimuli
spontaneous and evoked activity distributions are similar	✓(Fig. 7)	✓

Table S2. Related to Figs. 3–7. Generalization of the results with a GSM to a hierarchical inference model (see details in Section 7). Figure numbers in parentheses refer to the main text.

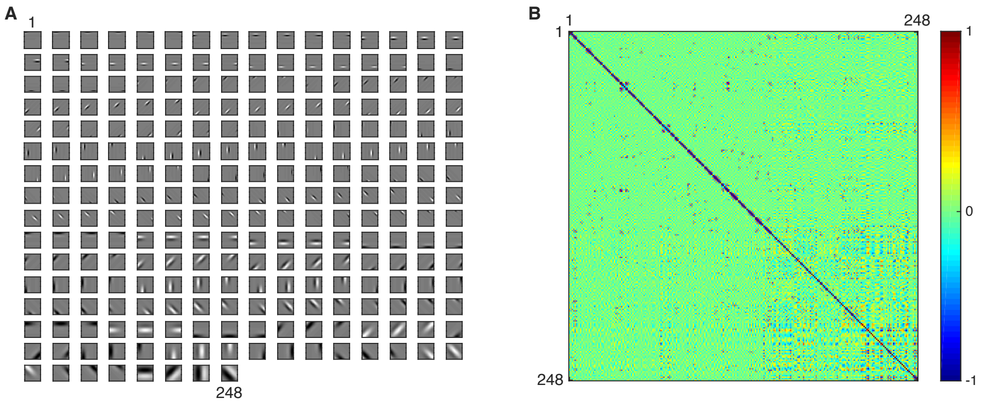

Figure S1. Related to Fig. 1. Model parameters. (**A**) Basis functions (columns of **A**) in the model. Each panel shows one of the 248 columns of **A** rearranged as a 16×16 image, revealing oriented, band-pass, localized filters that were synthetized using one of four orientations and four spatial frequencies. (**B**) Correlations of the prior covariance matrix of the model, obtained by training the model on natural images.

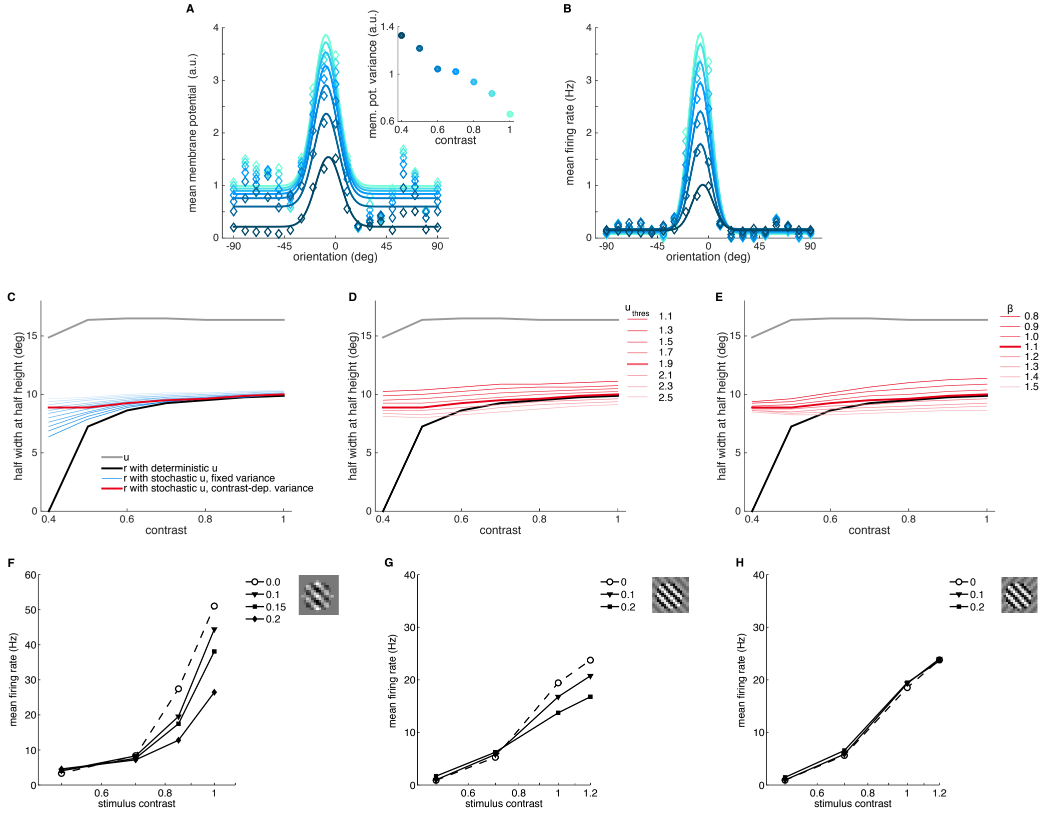

Figure S2 ***(continued on following page)*.**

Figure S2 ***(preceding page).*** **Related to Fig. 2.** Contrast-invariance and non-classical receptive field effects in a representative model neuron. (**A**-**B**) Contrast-dependence of membrane potential (**A**) and firing rate tuning curves (**B**) for orientation (diamonds). Different colors represent different contrast levels (for reference, see inset). Solid lines show circular Gaussian fits with baseline offset, amplitude, center, and width parameters fitted individually for each contrast level. Inset: membrane potential variance at different contrast levels. Colors as in the main main panel. (**C**) Half-width at half-height of different (actual and hypothetical) tuning curves as a function of contrast. Gray line: actual membrane potential tuning curves (from panel **A**); black line: hypothetical firing rate tuning curves assuming no noise variance in membrane potentials around membrane potential tuning curves; blue lines: hypothetical firing rate tuning curves assuming a fixed amount of membrane potential noise variance across different levels of contrast (from dark to light blue: fixed variance increases from that of the highest contrast posterior to the prior variance of the neuron); red line: actual firing rate tuning curves (from panel **B**) based on membrane potential noise variance that decreases with contrast (as shown in panel **A**, inset). (**D, E**) As in **C**, but showing the effects of varying the parameters of the firing rate nonlinearity (**D**: threshold, u_{thresh}; **E**: power-law exponent, β; see also Eq. 7 in Experimental Procedures). Thick red lines correspond to parameter values used in all other figures. (**F**) Cross-orientation suppression of mean firing rate responses. A grating stimulus (inset) with a range of contrast levels (x-axis) was presented at the neuron's preferred orientation while an orthogonally oriented grating stimulus was presented at a different contrast level (symbols). The orthogonal stimulus gradually suppressed (solid lines and filled symbols) the response given to the optimal stimulus alone (dashed line, open symbols). (**G, H**) Selective suppression of the mean firing rate response by non-classical receptive field stimulation. A grating stimulus (inset) with a range of contrast levels (x-axis) was presented at the neuron's preferred orientation in the classical receptive field while a parallel (**G**) or orthogonal grating stimulus (**H**) was presented in the surround non-classical receptive field at different contrast levels (symbols). The parallel surround stimulus gradually suppressed (solid lines and filled symbols, **G**) the response given to the center stimulus alone (dashed line, open symbols), while an orthogonally oriented surround stimulus left it unchanged (**H**).

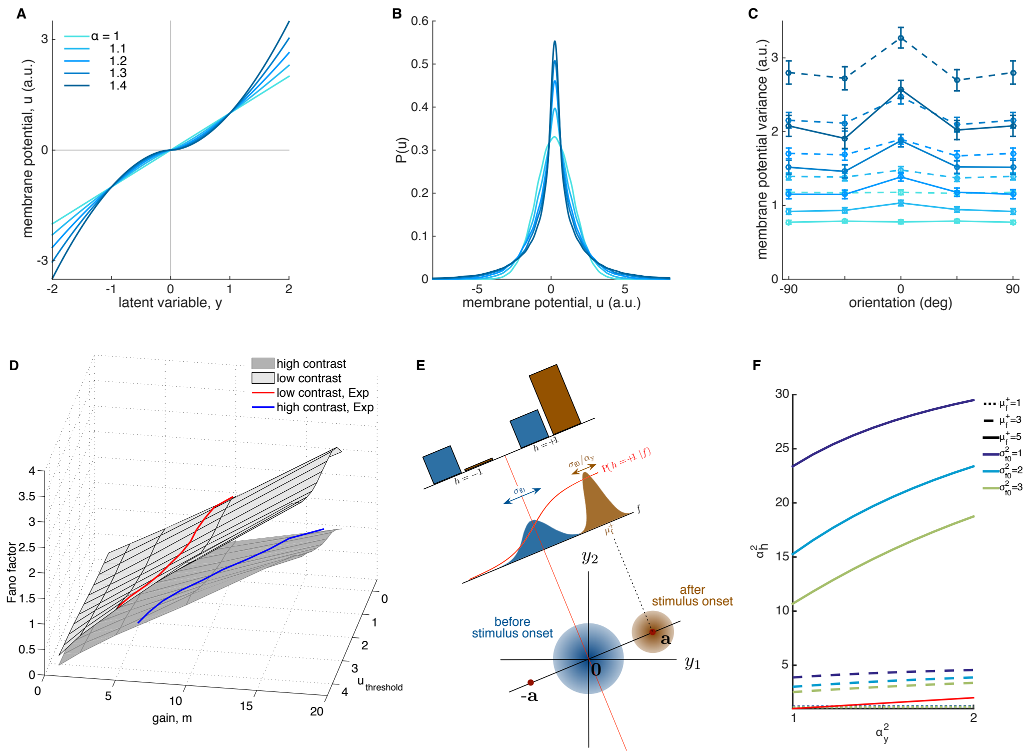

Figure S3 ***(continued on following page)*****.**

Figure S3 ***(preceding page).*** **Related to Figs. 3-4.** Response variability in the model: parameter dependence and implications for perceptual confidence. (**A-C**) Effect of membrane potential nonlinearity on response statistics. (**A**) Mapping from y to u for different values of the exponent of the nonlinearity, α (see also Eq. 6 of the main text). (**B**) Distribution of membrane potentials, u, at different values of α (colors as in **A**). Note sharper peak and larger tails for higher values of α. (**C**) Dependence of membrane potential variance on orientation as a function of α (colors as in **A**). Continuous lines represent the variance of high-contrast responses, dashed lines show the variance of low-contrast responses (cf. **Fig. 4C** of the main text). (**D**) Effect of firing rate nonlinearity on (mean matched) Fano factors. Two free parameters of the firing rate nonlinearity: threshold (u_{thresh}) and gain (m, Eq. 7 of the main text) are explored for spontaneous activity (light gray) and activity evoked by high contrast grating stimuli (dark gray). Red and blue lines show experimentally observed Fano factors (Churchland et al., 2010). (**E**) Schematic of inference under a hierarchical model. y_1, y_2: low (V1-)level variables, f: their summary statistic relevant for a perceptual decision, h: high-level (binary) perceptual decision variable. Blue and brown distributions respectively show posteriors before and after stimulus onset, red sigmoid curve and straight line show classification function and boundary relating $\mathbf{y}$ and f to h. Note that the large shift in h towards $+1$ at stimulus onset, and the consequent drastic reduction in its variance, is mainly driven by a shift of the *mean* of $\mathbf{y}$ away from the classification boundary, rather than the relatively modest reduction in the *variance* of $\mathbf{y}$. See text (Section 7) for further details. (**F**) Relationship between the reduction in low-level variance, α_{y}^2 (as for our simulated membrane potential variances), and the reduction in high-level variance, α_{h}^2 (as reflected in perceptual experience), at stimulus onset in a simple hierarchical model of perceptual decision making as parametrised by different levels of 'signal', μ_{f}^{+} (dotted, dashed, and solid lines), and 'noise', σ_{f0}^2 (dark blue, light blue, green). The red line shows the identity transformation for reference. See text (Section 7) for details.

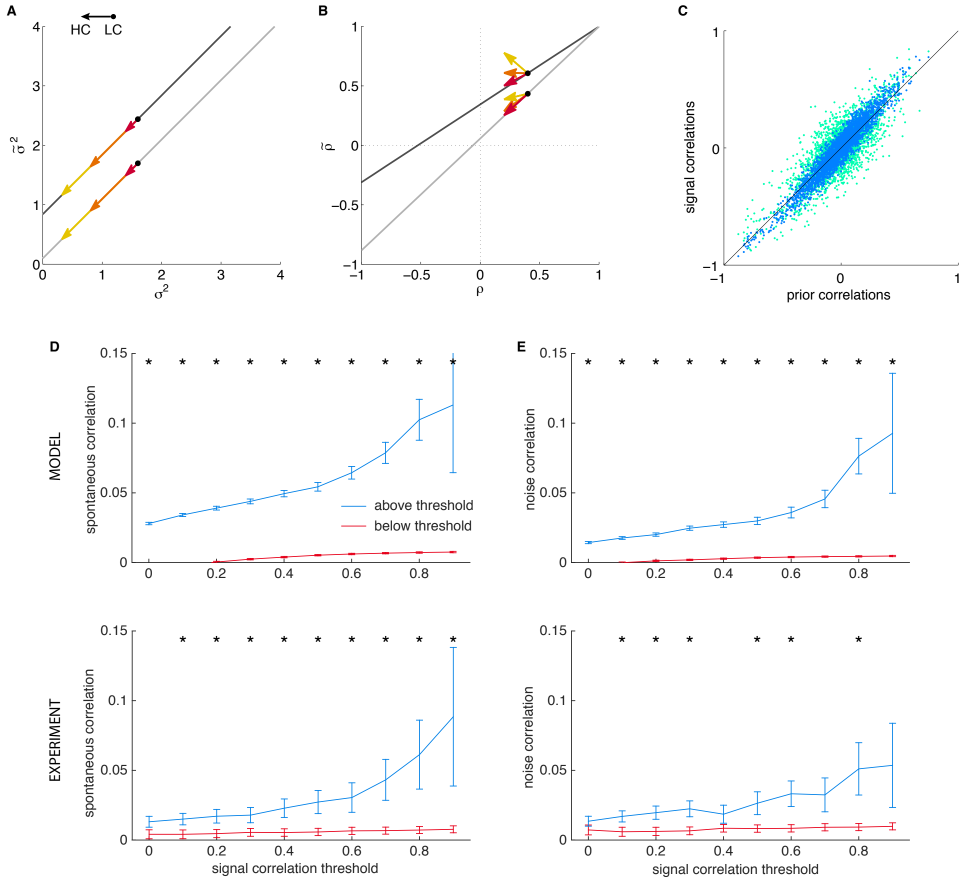

Figure S4 ***(continued on following page)***

Figure S4 *(preceding page).* Related to Figs. 4-6. Response correlations. (**A,B**) Effect of synchronized fluctuations on membrane potential variance and cross-correlation, respectively. A change from low contrast (LC) to high contrast (HC) reduces within-state membrane potential variance (σ^2) and correlation (ρ) which in turn cause changes in overall membrane potential variance ($\tilde{\sigma}^2$) and correlation ($\tilde{\rho}$). Arrows point from LC to HC values, different colors correspond to different magnitudes of within-state variance reduction (see below). Grey lines correspond to different state-variance magnitudes, $\omega^2 = 0.0975\,\mathrm{mV}^2$ (light gray) and $\omega^2 = 0.84\,\mathrm{mV}^2$ (dark gray). For illustration, based on Haider et al. (2013) reporting bimodal membrane potential distributions in anesthesia, a mixture of two Gaussians was used for modelling response distributions with the following parameters: fraction of low membrane potential state $p = 0.3$ (dark gray) or 0.025 (light gray), difference between high and low baseline membrane potential $\Delta_\mu = 2\,\mathrm{mV}$, $\sigma^2_{HC} = 1.6\,\mathrm{mV}^2$, $\rho_{HC} = 0.4$, $\sigma^2_{LC} = \sigma^2_{HC} \cdot [1.15, 2, 5]$ for red, orange, and yellow arrows, respectively, $\rho_{LC} = 0.2$. (**C**) Prior correlations in the model reflect signal correlations. Correlation between prior and signal membrane potential correlations across all pairs of neurons during natural image presentation was 0.95 (blue dots), and 0.79 when grating stimuli were used to compute signal correlations (green dots). (**D,E**) Robustness of the signal correlation-dependence of spontaneous (**D**) and noise correlations (**E**). Different thresholds for splitting signal correlations were tested (x-axis), lines show mean (±s.e.) spontaneous and noise correlations across cell pairs with above- (blue line) and below-threshold (red line) signal correlations, $*p < 0.05$. Cf. **Fig. 6** of the main text, showing analysis of same data (for both model and experiments) with a threshold of 0.5.

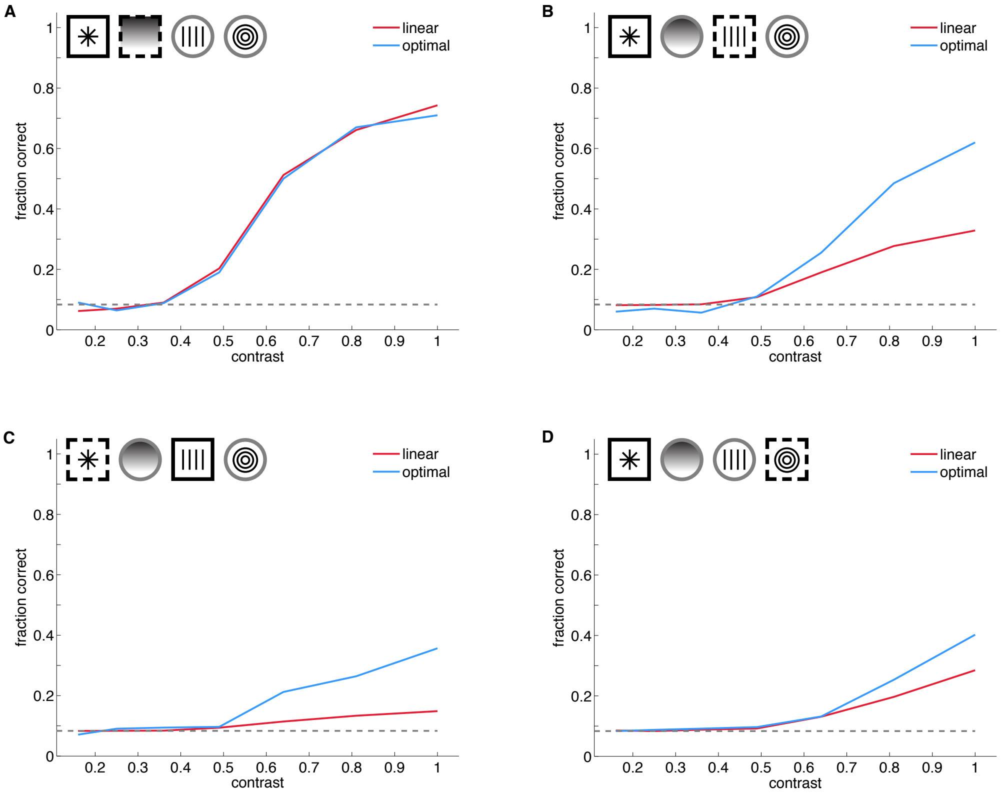

Figure S5. Related to Figs. 4-6. Decoding performance of a linear and an optimal decoder. Panels show four decoding tasks, differing in the role of four stimulus attributes (insets: orientation, contrast, phase, and aperture size of a sinusoidal grating stimulus). In each task, two attributes were kept fixed (icons in gray circles), and two varied (icons in black squares), out of which one had to be decoded (solid contour), and one ignored (nuisance parameter, dashed contour). (**A**) Decoder performance when the stimulus attribute to be decoded was orientation, and contrast needed to be ignored. Stimulus phase and aperture size were fixed. Dashed line denotes chance level. The linear decoder was trained using grating images of which the contrast was uniformly distributed among seven discrete levels, and test performance is shown at each contrast level separately. The optimal decoder (blue) was tested on the same stimuli as the linear decoder (red). (**B-D**) Similar to **B**, but with different stimulus attributes as the decoded and nuisance parameters.

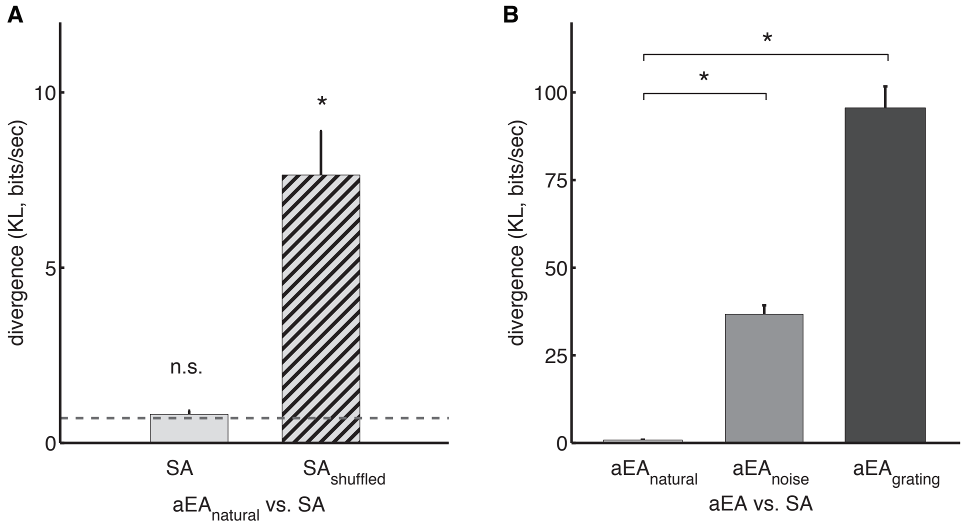

Figure S6. Related to Fig. 7. Match between spontaneous (SA) and average evoked activity (aEA) distributions in the model depends on correlations. (**A**) and the stimulus ensemble used (**B**). Well isolated units were simulated in which the spike count of each model neuron was separately recorded (c.f. **Fig. 7** of the main text, in which multiunit activity was simulated by combining the spike counts of several model neurons in each unit). (**A**) Kullback-Leibler (KL) between aEA for natural image patches ($aEA_{natural}$) and SA (light grey bar), and between $aEA_{natural}$ and a shuffled version of SA, preserving individual firing rates but destroying all correlations across electrodes ($SA_{shuffled}$, hatched bar). For reference, baseline KL divergence between two halves of SA data is also shown (dashed line). (**B**) KL divergence of SA from aEA under three different stimulus conditions: natural image patches ($aEA_{natural}$, light grey bar, same as in panel **A**), random block noise images (aEA_{noise}, dark grey bar), and grating stimuli with various phases, orientations, and frequencies ($aEA_{grating}$, black bar). In both panels, bars show averages across multiple simulations, error bars show s.e., $*p < 0.05$.
